# Supplementary material for: BNIP3L/NIX regulates both mitophagy and pexophagy
Source: EMBO J. 2022 Oct 10;41(24):e111115. doi: 10.15252/embj.2022111115 (PMC9753467; doi:10.15252/embj.2022111115)
Supplement: Supplementary file 1 — Expanded View Figures PDF [file EMBJ-41-e111115-s006.pdf]

Expanded View Figures

Figure EV1. Analysis of pexophagy upon iron chelation and hypoxia.

A Representative immunoblots of the indicated proteins in lysates of ARPE-19 cells treated 48 h with 1 mM DFP.

B, C Representative immunoblots of the indicated proteins in lysates of HeLa and SH-SY5Y cells (B) or ARPE-19 cells (C), treated with 1 mM DFP or 50 μM Phenentroline (Phen) for the indicated time (h).

D Representative immunoblots of the indicated proteins in lysates of ARPE-19 cells stimulated with hypoxia (0.8%) for 3 days.

E Representative confocal images of ARPE-19 cells stably expressing the *pexo*-QC reporter stimulated with hypoxia (0.8%) for 3 days and at right, quantification of total red-only punctate per cell (pexolysosomes). Enlarged images of the area outlined in white are shown in the lower corners.

F, G Representative confocal images of ARPE-19 cells stably expressing the *pexo*-QC reporter treated with 1 mM DFP for 48 h and immunostained with peroxisomal markers (F, Catalase and PMP70), or lysosomal marker (G, LAMP1). Enlarged images of the area outlined in white are shown on the right or below.

H, I ARPE-19 cells expressing the *pexo*-QC reporter were transfected with 50 pmol of non-targeting siRNA (siNT) or 50 pmol of siRNA targeting ATG13 or ULK1. 48 h post transfection, cells were treated with 1 mM DFP for an additional 48 h and analysed by immunoblot (H) or by flow cytometry (I). Representative dot plots are shown after analysing GFP and mCherry signals. The percentage of cell underdoing pexophagy (purple population) is indicated in bold purple on each dot plot.

Data information: Overall data are mean ± s.d.; $n = 3$ biological replicates. *$P \leq 0.05$; ****$P \leq 0.0001$; ns, not significant (two-way ANOVA, Sidak's multiple comparisons test). Nuclei were stained in blue (Hoechst) and scale bars: 10 μm.
Source data are available online for this figure.

© 2022 The Authors

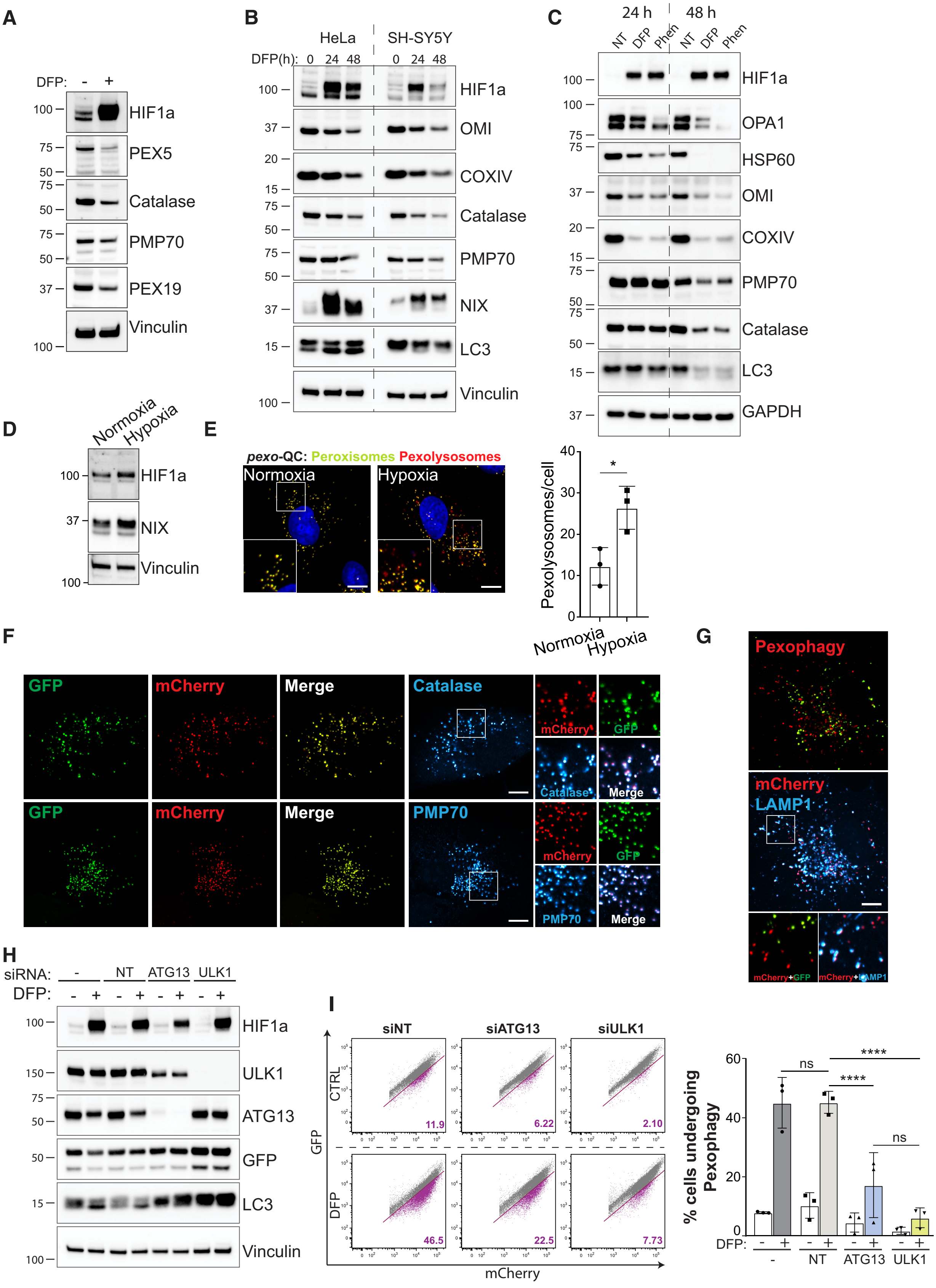
A
DFP: - +
HIF1a
PEX5
Catalase
PMP70
PEX19
Vinculin
B
HeLa
SH-SY5Y
DFP(h): 0 24 48 0 24 48
HIF1a
OMI
COXIV
Catalase
PMP70
NIX
LC3
Vinculin
C
24 h
48 h
NT DFP Phen NT DFP Phen
HIF1a
OPA1
HSP60
OMI
COXIV
PMP70
Catalase
LC3
GAPDH
D
Normoxia Hypoxia
HIF1a
NIX
Vinculin
E
pexo-QC: Peroxisomes Pexolysosomes
Normoxia
Hypoxia
Pexolysosomes/cell
Normoxia Hypoxia
F
GFP
mCherry
Merge
Catalase
PMP70
G
Pexophagy
mCherry LAMP1
mCherry+GFP
mCherry+LAMP1
H
siRNA: - NT ATG13 ULK1
DFP: - + - + - + - +
HIF1a
ULK1
ATG13
GFP
LC3
Vinculin
I
siNT
siATG13
siULK1
CTRL
DFP
GFP
mCherry
11.9 6.22 2.10
46.5 22.5 7.73
% cells undergoing Pexophagy
ns
****
****
ns
DFP: - + - + - + - +
- NT ATG13 ULK1

Figure EV1.

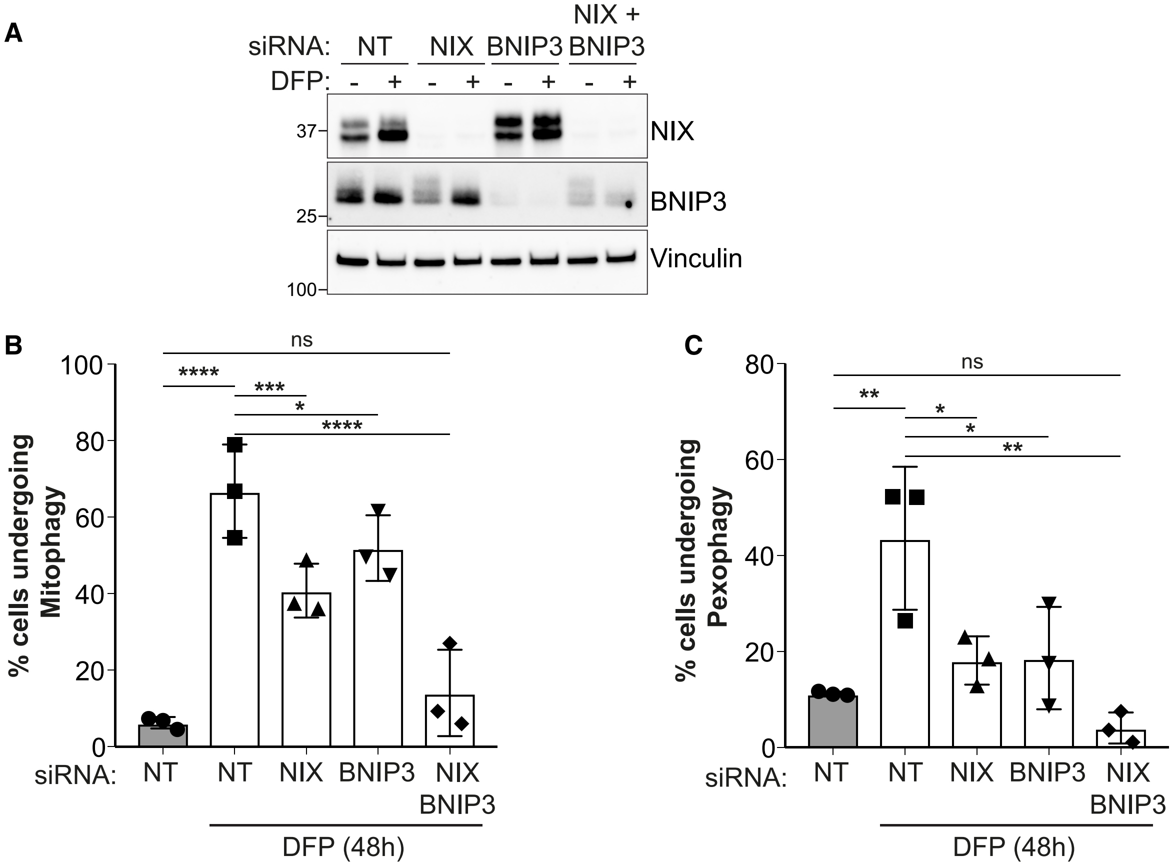

Figure EV2. BNIP3 is involved in mitophagy and pexophagy.

A Representative immunoblots of the indicated proteins in lysates of ARPE-19 cells transfected with 50 pmol of non-targeting siRNA (NT) or 50 pmol of siRNA targeting NIX or BNIP3. 48 h post transfection, cells were treated with 1 mM DFP for an additional 48 h.

B, C Quantification by flow cytometry of the mCherry/GFP ratio in ARPE-19 cells expressing the *mito*-QC reporter (B) or the *pexo*-QC reporter (C) and treated as in (A).

Data information: Overall data are mean ± s.d.; $n = 3$ biological replicates. *$P \leq 0.05$; **$P \leq 0.01$; ***$P \leq 0.001$; ****$P \leq 0.0001$; ns, not significant. One-way ANOVA, Tukey's multiple comparisons test.
Source data are available online for this figure.

Figure EV3. Analysis of NIX localisation.

A Representative confocal images of ARPE-19 cells immunostained with an anti-NIX antibody (green) and anti-catalase antibody (red) after 1 mM DFP for the indicated time (h). Peroxisomes enriched for NIX or having minimal signal for NIX are depicted with yellow or white arrowheads, respectively.

B Representative immunoblots and quantifications (below) of the indicated proteins in lysates of cells treated as (A). Data are mean ± s.d.; $n = 4$ biological replicates. *$P \leq 0.05$; **$P \leq 0.01$; ns, not significant (one-way ANOVA, Tukey's multiple comparisons test).

C Representative confocal images of ARPE-19 cells WT (right panel) or stably expressing GFP-NIX (left panel) and stimulated with hypoxia for 3 days. Peroxisomes were stained with anti-catalase antibody (red) and endogenous NIX were stained with anti-NIX antibody (green, right panel).

D Representative confocal images of untreated (CTRL) and DFP-treated ARPE-19 cells (1 mM, 48 h), stably expressing GFP-BNIP3 (green) and stained with anti-catalase antibody (red). At right, quantification of Pearson correlation coefficients between BNIP3 and the peroxisomal marker catalase after DFP treatment for 48 h.

E Schematic representation of the NIX mutants. Right panel, confocal images of ARPE-19 cells stably expressing GFP-NIX mutants and immunostained with a mitochondrial marker (ATPB, red).

Data information: Enlarged images of the area outlined in white are shown on the right. Nuclei were stained in blue (Hoechst) and scale bars: 10 µm.
Source data are available online for this figure.

 © 2022 The Authors

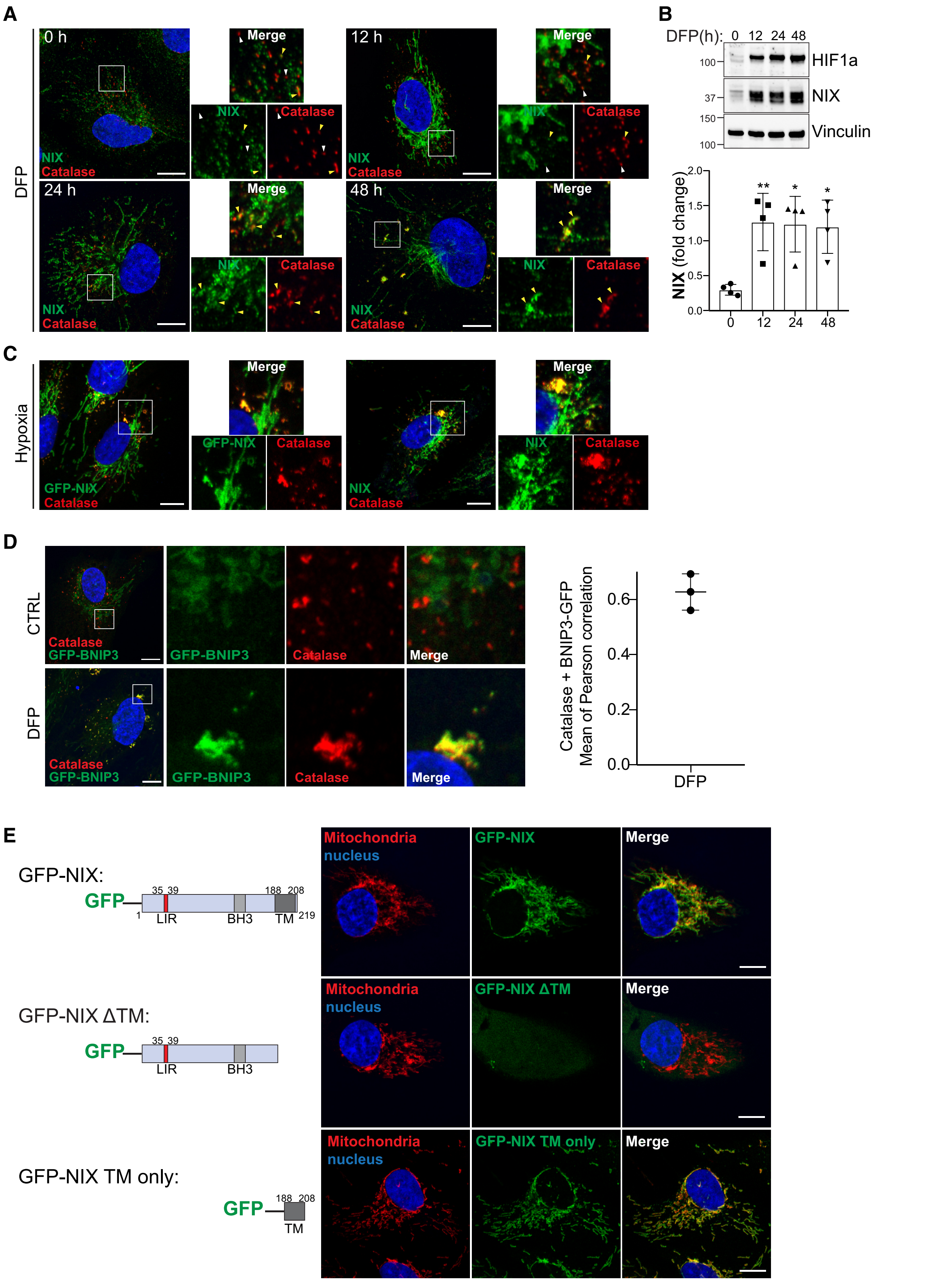
A
0 h
12 h
24 h
48 h
DFP
Merge
NIX
Catalase
B
DFP(h): 0 12 24 48
HIF1a
NIX
Vinculin
100
37
150
100
NIX (fold change)
2.0
1.5
1.0
0.5
0.0
**
*
*
0
12
24
48
C
Hypoxia
GFP-NIX
Catalase
Merge
NIX
D
CTRL
DFP
Catalase
GFP-BNIP3
Merge
Catalase + BNIP3-GFP
Mean of Pearson correlation
0.6
0.4
0.2
0.0
DFP
E
GFP-NIX:
GFP
35 39
188 208
1
LIR
BH3
TM
219
GFP-NIX ΔTM:
GFP-NIX TM only:
Mitochondria
nucleus
GFP-NIX
GFP-NIX ΔTM
GFP-NIX TM only
Merge

Figure EV3.

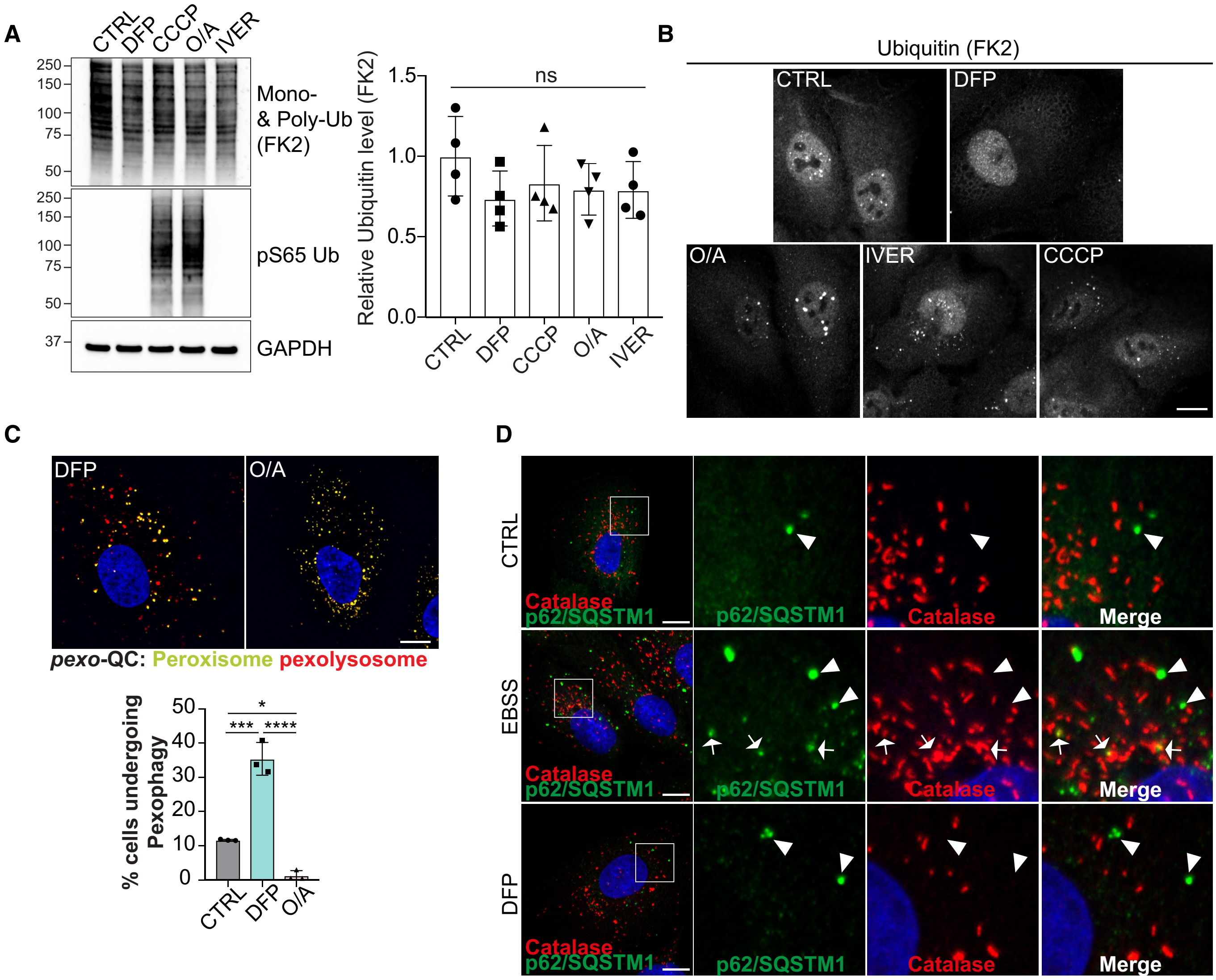

Figure EV4. Ubiquitylation status upon DFP treatment.

A Representative immunoblots and quantification of the indicated proteins in lysates of ARPE-19 cells treated with 1 mM DFP, 20 μM CCCP, 10 μM Antimycin and 5 μM Oligomycin A (O/A), 15 μM Ivermectin for 24 h.

B Representative confocal images of ARPE-19 cells treated as in A and stained with anti-Ubiquitin antibody (FK2).

C Representative confocal images of ARPE-19 cells stably expressing the *pexo*-QC reporter and treated with 1 mM DFP or 10 μM Antimycin and 5 μM Oligomycin A (O/A) for 48 h. Below, flow cytometry analysis of the mCherry/GFP ratio.

D Representative confocal images of ARPE-19 cells treated with EBSS for 24 h or 1 mM DFP for 48 h and immunostained with anti-catalase (red) and anti-p62 (green) antibodies. Autophagosomes containing peroxisomes are depicted with white arrows while autophagosomes not colocalizing with peroxisome marker are pointed with white arrowheads. Enlarged images of the area outlined in white are shown on the right.

Data information: Data are mean ± s.d.; Statistical significance is displayed as *$P \leq 0.05$; ***$P \leq 0.001$; ****$P \leq 0.0001$; ns, not significant. (A) $n = 4$ biological replicates, (C) $n = 3$ biological replicates; one-way ANOVA, Tukey's multiple comparisons test.

 © 2022 The Authors

Figure EV5. Involvement of NIX dimerisation in mitophagy and pexophagy.

A Representative immunoblots of the indicated proteins in lysates of NIX KO ARPE-19 cells (Cl 31) stably expressing a pBabe Flag vector (∅), a pBabe Flag-NIX vector (NIX), a pBabe Flag-NIX G204A vector (G204A), and treated with 1 mM DFP for 48 h prior lysis.

B Representative confocal images of cells as in (A), stably expressing the *mito*-QC reporter or the *pexo*-QC reporter and treated with 1 mM DFP for 48 h (left panel) and flow cytometry analysis of the mCherry/GFP ratio (right panel). Enlarged images of the area outlined in white are shown in the lower corners.

C, D Representative confocal images of NIX KO ARPE-19 cells (Cl 31) stably expressing a pBabe Flag vector (∅), a pBabe Flag-NIX vector (NIX), a pBabe Flag-NIX W36A \L39A vector (W36A\L39A) or a pBabe Flag-NIX G204A vector (G204A), untreated (C) or treated with 1 mM DFP for 48 h (D) and immunostained with a mitochondrial marker (C, ATPB in red) or a peroxisomal marker (D, Catalase in red) and with an anti-NIX antibody (green).

Data information: Nuclei were stained in blue (Hoechst). Scale bars: 10 μm. Data are mean ± s.d.; Statistical significance is displayed as ***$P \leq 0.001$; ****$P \leq 0.0001$; ns, not significant. (B) $n = 3$ biological replicates; one-way ANOVA, Tukey's multiple comparisons test.
Source data are available online for this figure.

© 2022 The Authors

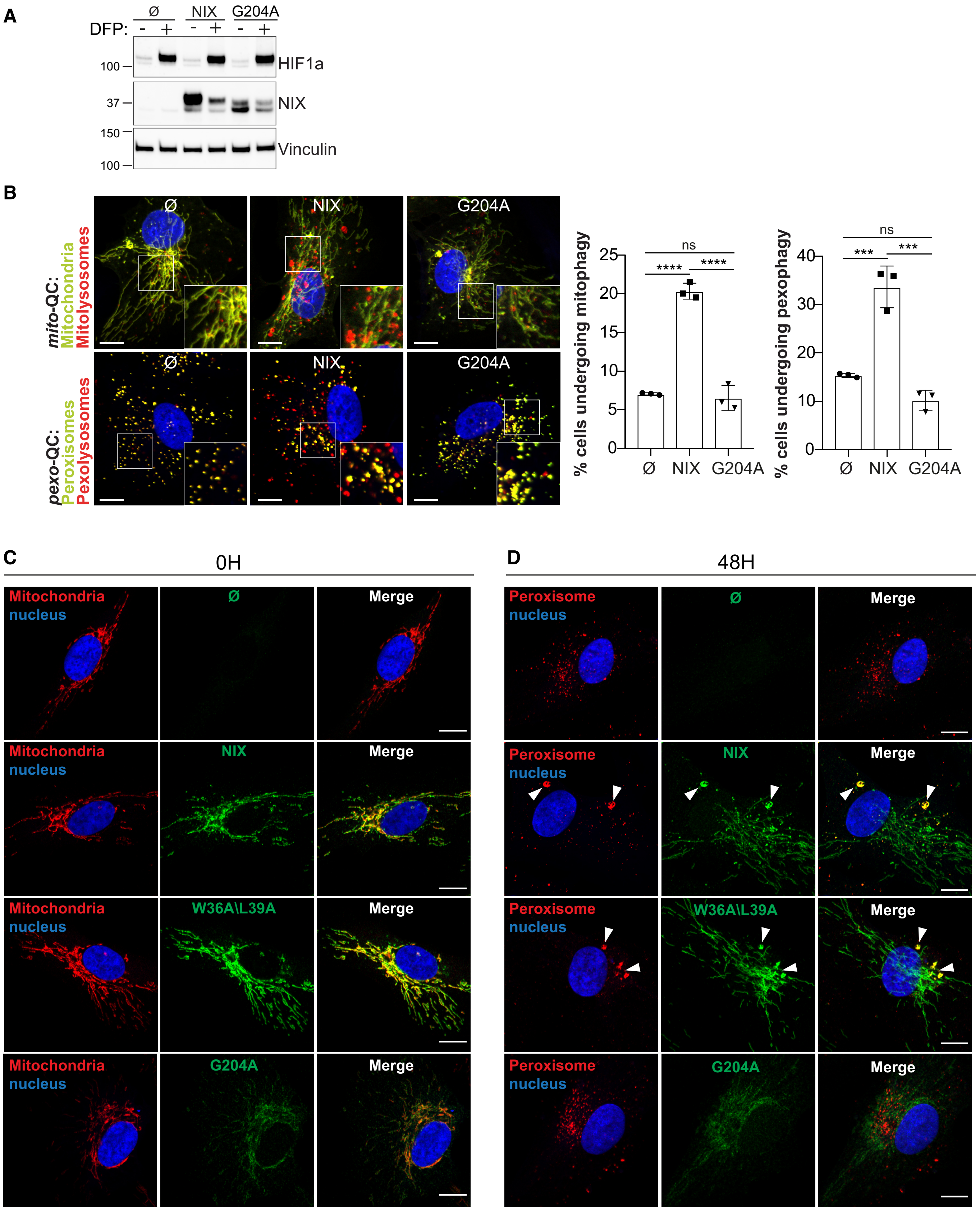

Figure EV5.

 © 2022 The Authors

Figure EV6. Pexophagy occurs during differentiation.

A Representative flow cytometry data of *in vitro* human erythroid cells cultured for 7, 10 and 13 days, as well as of reticulocytes obtained after 20 days in culture. The dot plots show unstained cells (black), cells stained with IgG control antibodies (grey) and cells stained with antibodies against alpha 4 integrin and Band 3 (coloured).

B Representative images of erythroblasts or reticulocytes as in (A), prepared by cytospin and stained with May-Grünwald's stain and Giemsa's stain. Scale bars: 10 μm.

C Immunoblots of the indicated proteins in lysates of erythroblasts or reticulocytes obtained from three different donors.

D Representative immunoblots of the indicated proteins in lysates of *mito*-QC- or *pexo*-QC-H9c2 cells during differentiation.

E Representative confocal images of *pexo*-QC reporter H9c2 cells before (D0) and after 8 days of differentiation (D8). Nuclei were stained in blue (Hoechst). Red-only puncta (pexolysosomes) are depicted with white arrowheads. At right, quantification of total number of pexolysosomes counted per cell analysed during cadiomyocyte differentiation. Overall data are mean ± s.d.; $n = 3$ biological replicates with > 85 cells per condition and replicate, unpaired *t*-test, two-tailed. $^{**}P \leq 0.01$.

F, G Representative confocal images of differentiated *pexo*-QC reporter H9c2 cells immunostained with anti-catalase (F) or anti-LAMP1 (G). White arrowheads indicate structures positive for both red-puncta and LAMP1. Enlarged images of the area outlined in white are shown below.

Data information: Scale bar: 10 μm.
Source data are available online for this figure.

© 2022 The Authors

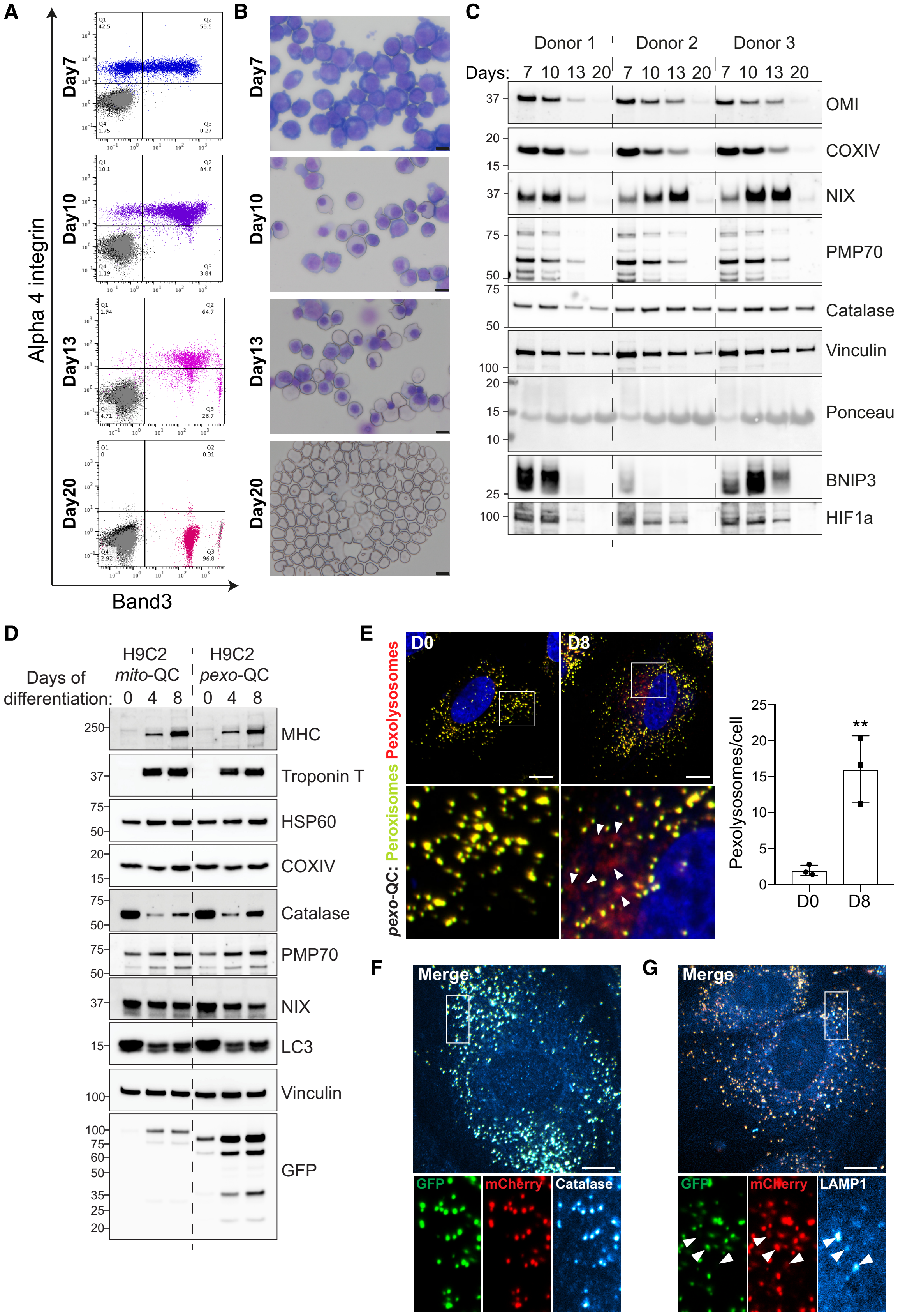
A
Alpha 4 integrin
Band3
Day7
Day10
Day13
Day20
B
Day7
Day10
Day13
Day20
C
Donor 1
Donor 2
Donor 3
Days: 7 10 13 20 7 10 13 20 7 10 13 20
OMI
COXIV
NIX
PMP70
Catalase
Vinculin
Ponceau
BNIP3
HIF1a
D
H9C2 mito-QC
H9C2 pexo-QC
Days of differentiation: 0 4 8 0 4 8
MHC
Troponin T
HSP60
COXIV
Catalase
PMP70
NIX
LC3
Vinculin
GFP
E
D0
D8
pexo-QC: Peroxisomes Pexolysosomes
Pexolysosomes/cell
D0
D8
**
F
Merge
GFP
mCherry
Catalase
G
Merge
GFP
mCherry
LAMP1

Figure EV6.
