# Supplementary figures and images for: BNIP3L/NIX regulates both mitophagy and pexophagy
Source: EMBO J. 2022 Oct 10;41(24):e111115. doi: 10.15252/embj.2022111115 (PMC9753467; doi:10.15252/embj.2022111115)

Figure EV1A

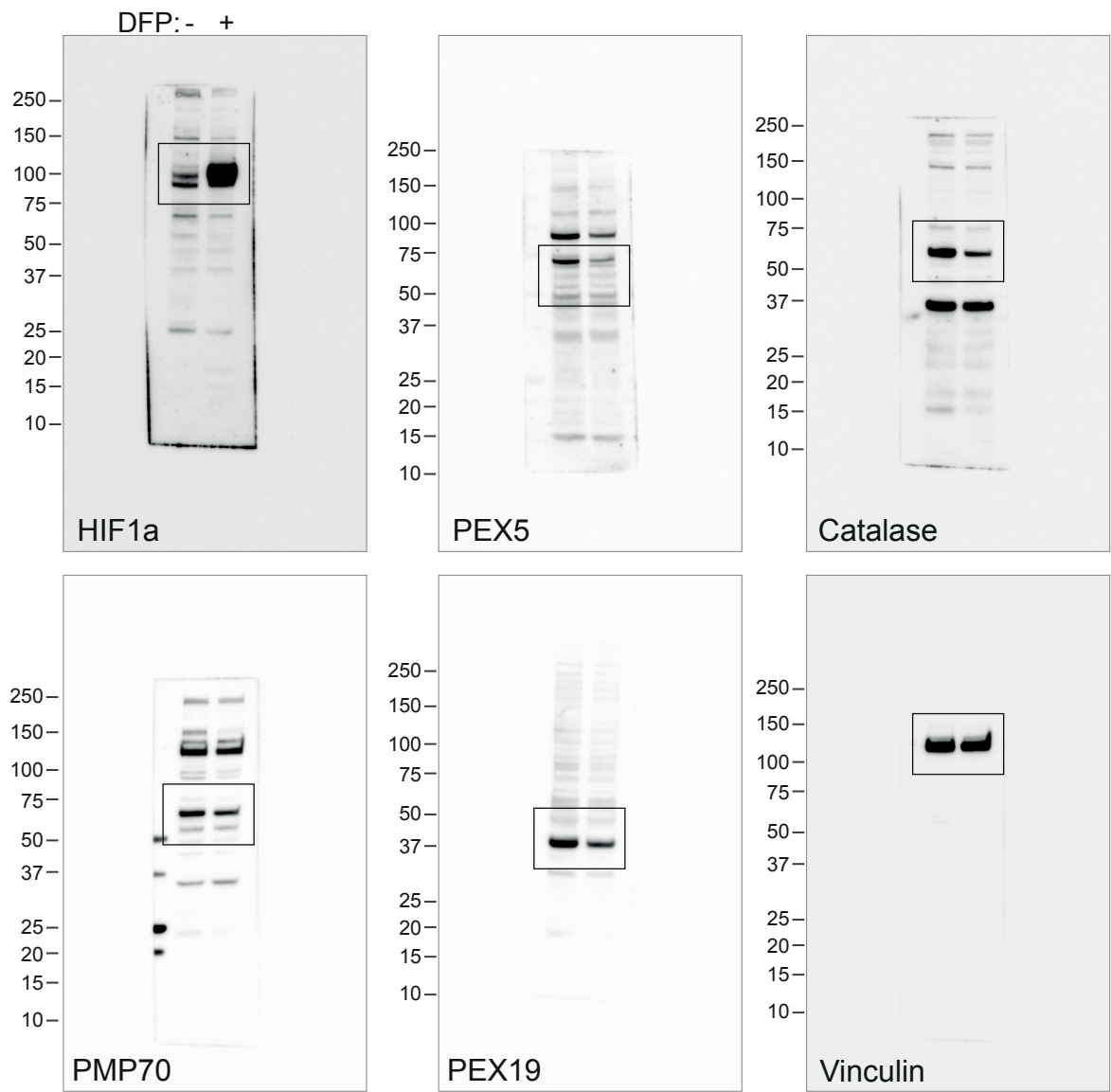

Figure EV1B

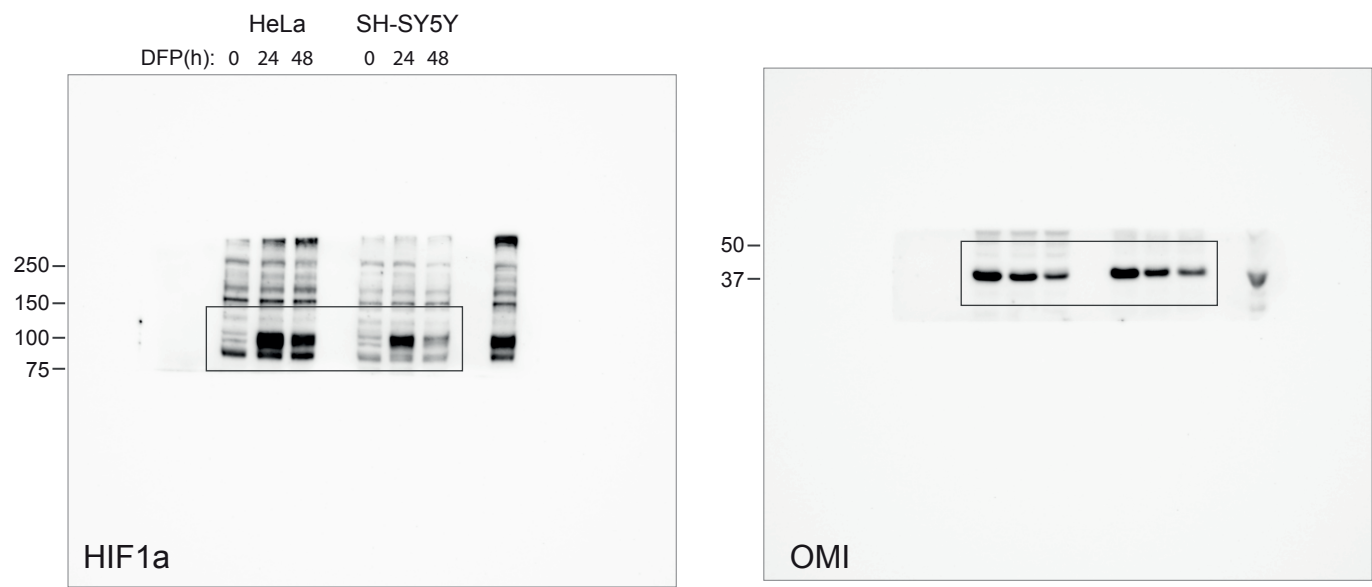

Figure EV1B

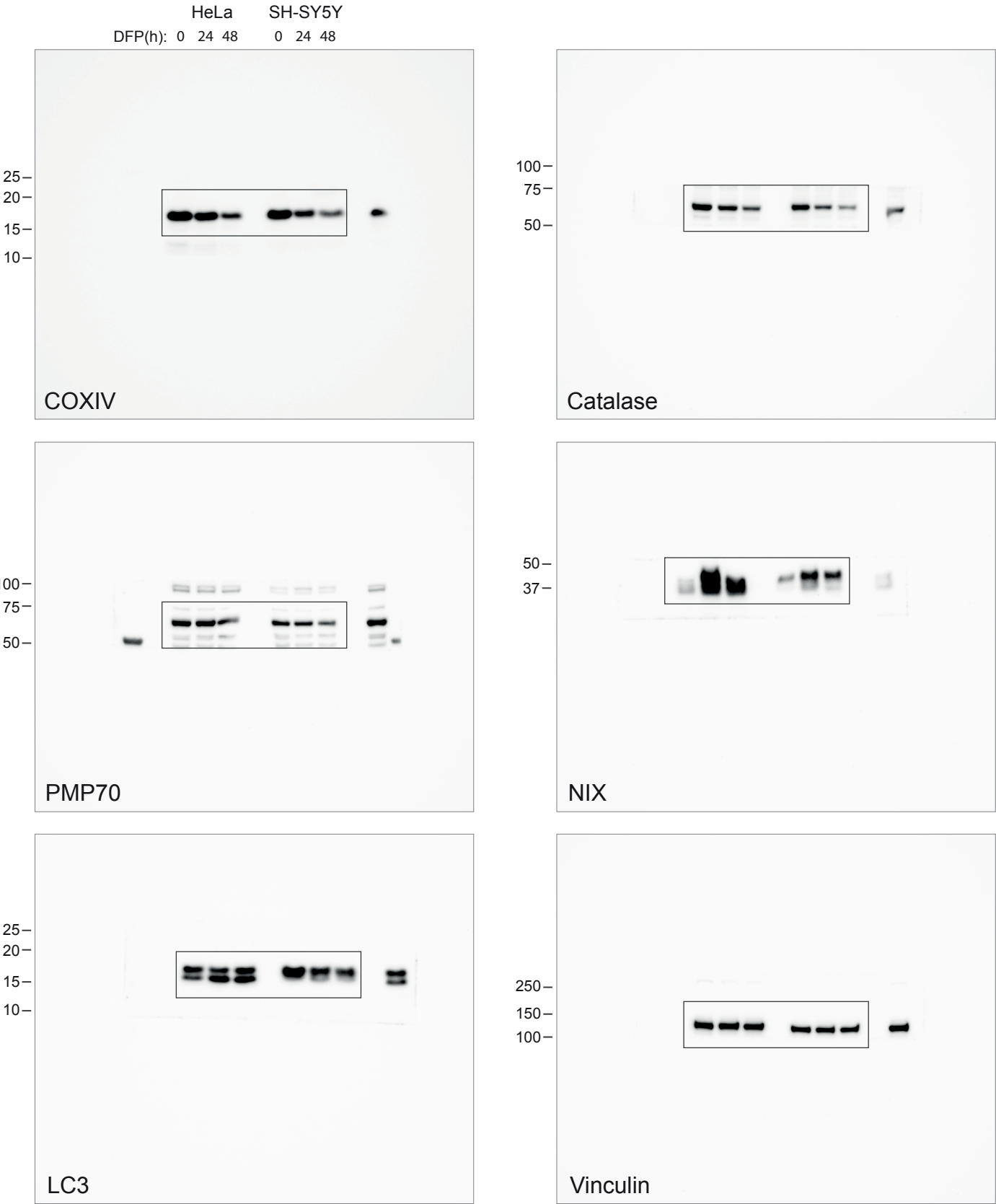

Figure EV1C

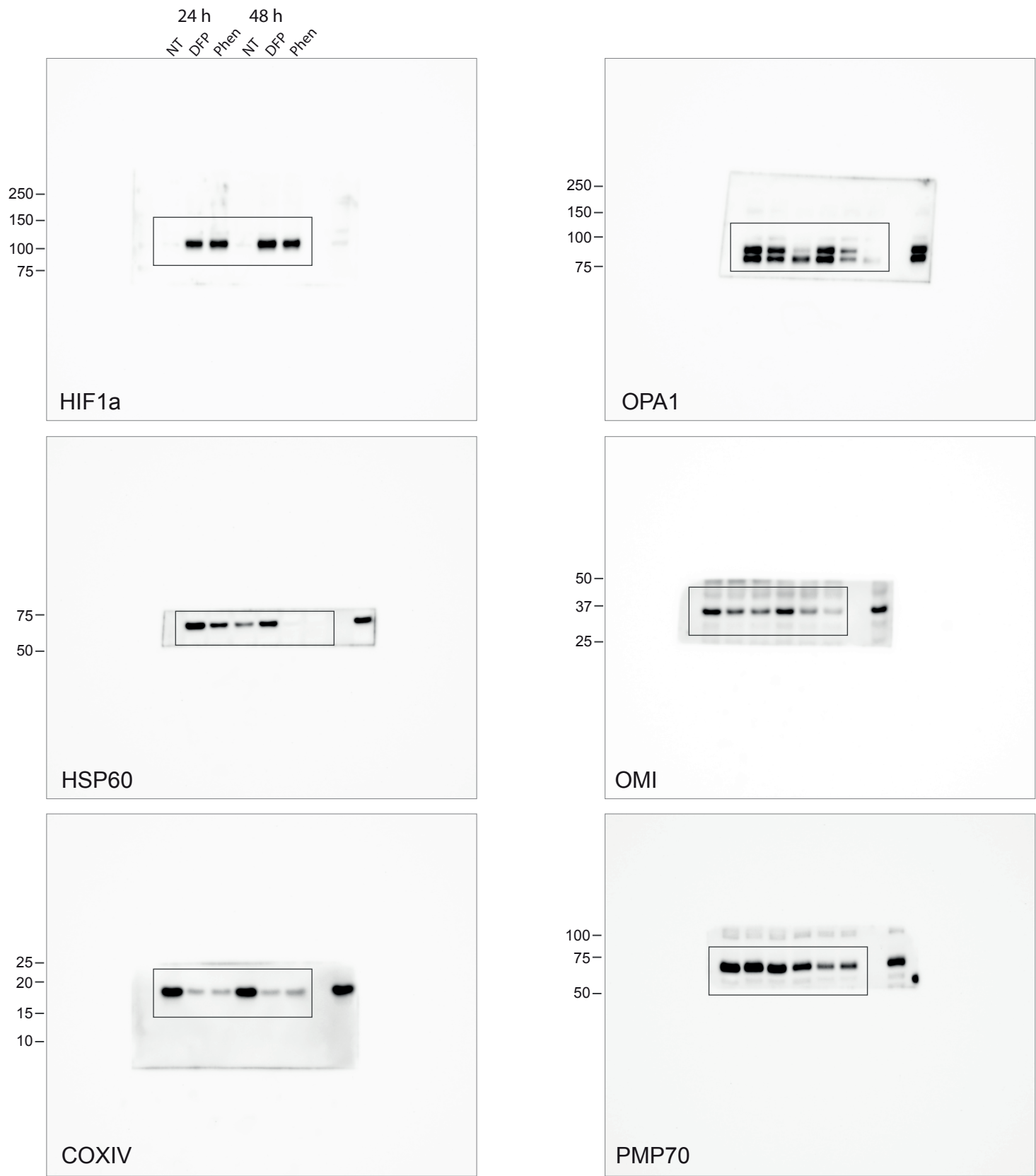

Figure EV1C

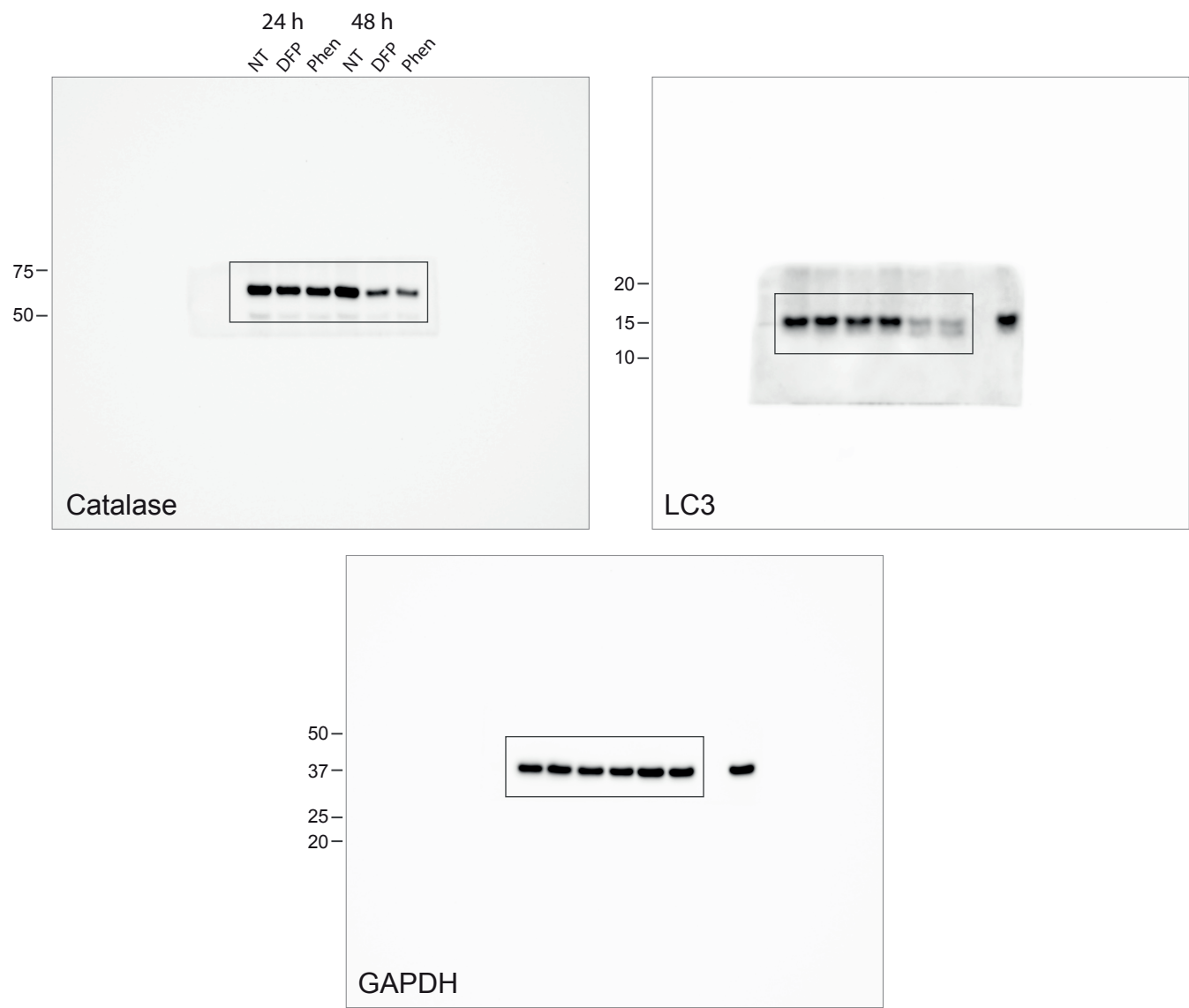

Figure EV1D

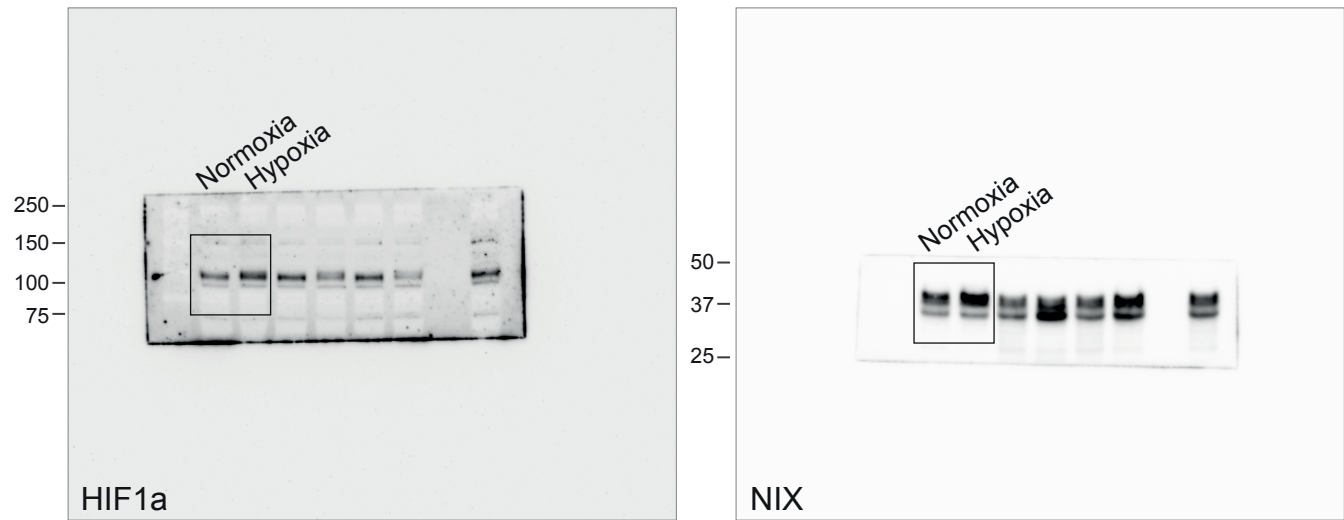

Figure EV1H

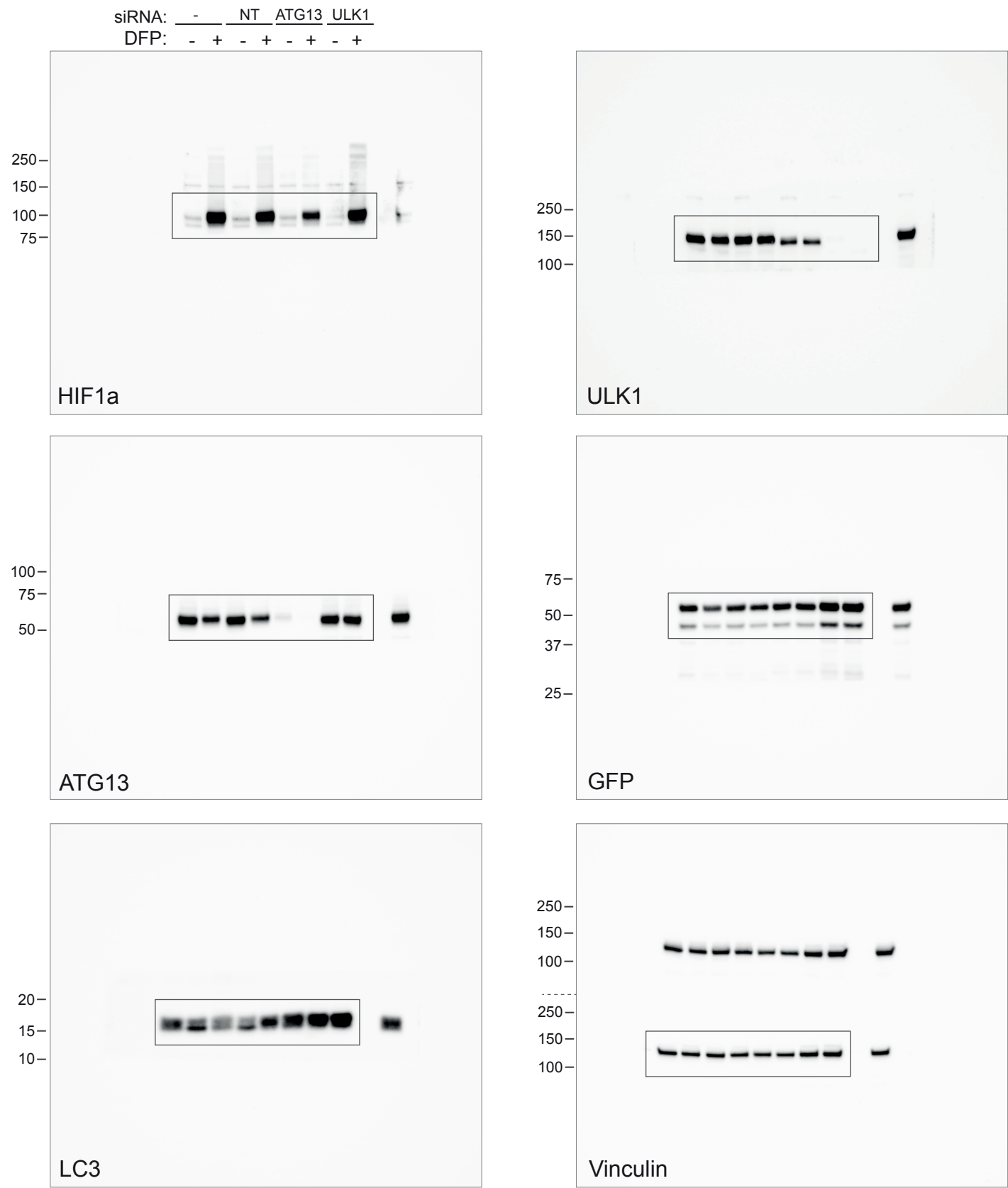

Supplement: Supplementary file 2 — Source Data for Expanded View [file EMBJ-41-e111115-s005.zip › EMBOJ-2022-111115R1-Figure_EV1_Source_Data-sd.pdf]

Figure EV2

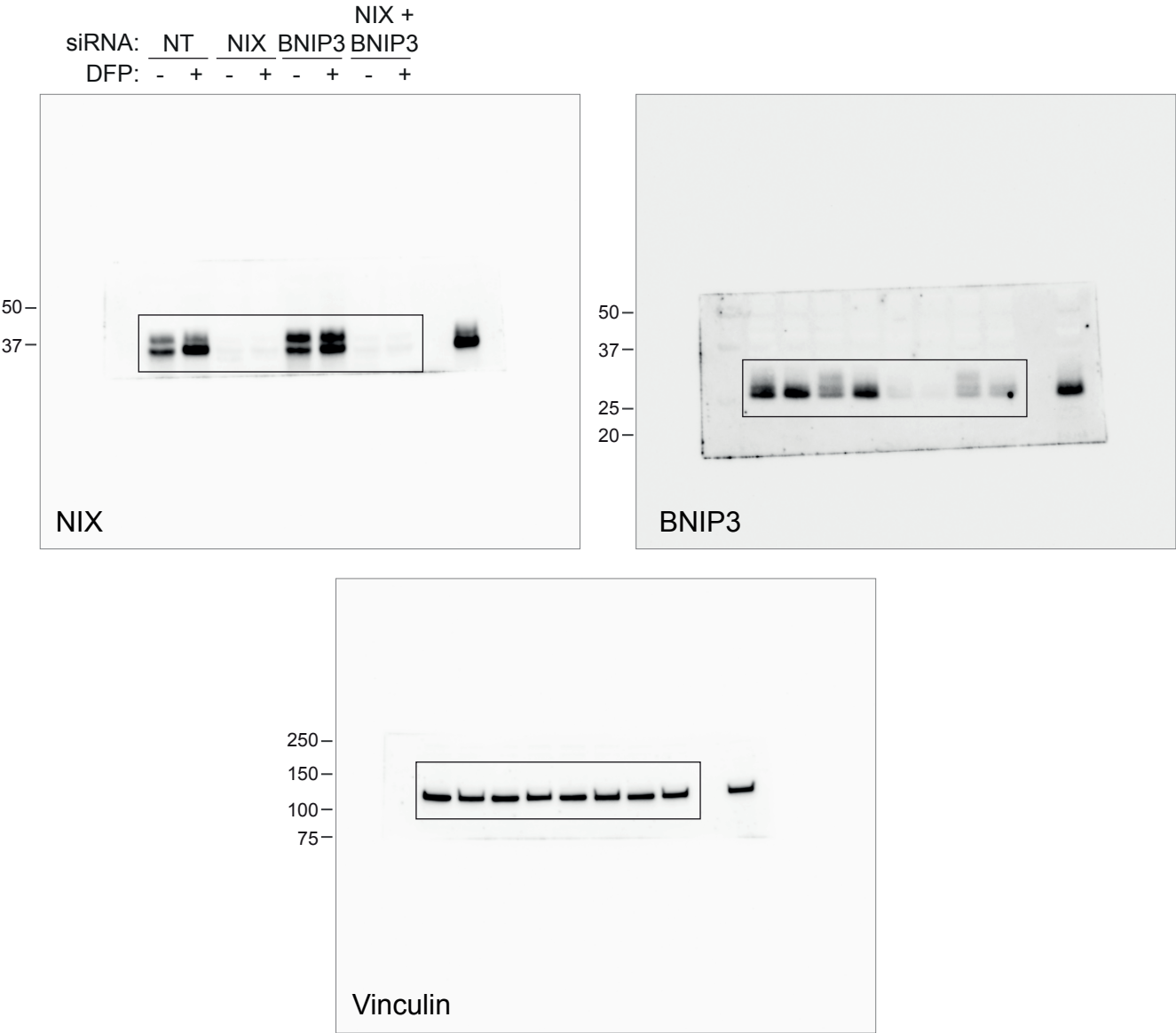

Supplement: Supplementary file 2 — Source Data for Expanded View [file EMBJ-41-e111115-s005.zip › EMBOJ-2022-111115R1-Figure_EV2_Source_Data-sd.pdf]

Figure EV3

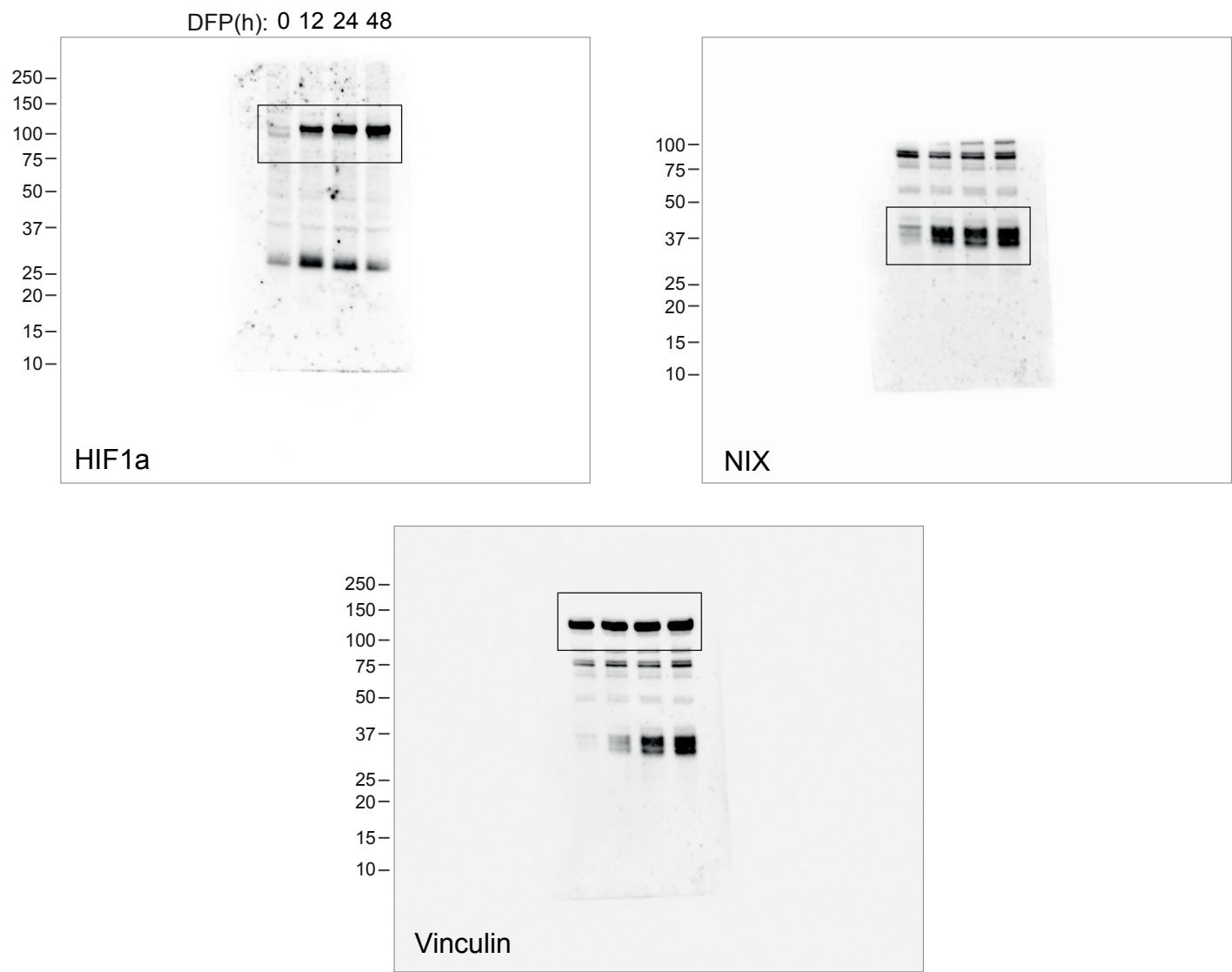

Supplement: Supplementary file 2 — Source Data for Expanded View [file EMBJ-41-e111115-s005.zip › EMBOJ-2022-111115R1-Figure_EV3_Source_Data-sd.pdf]

Figure EV5A

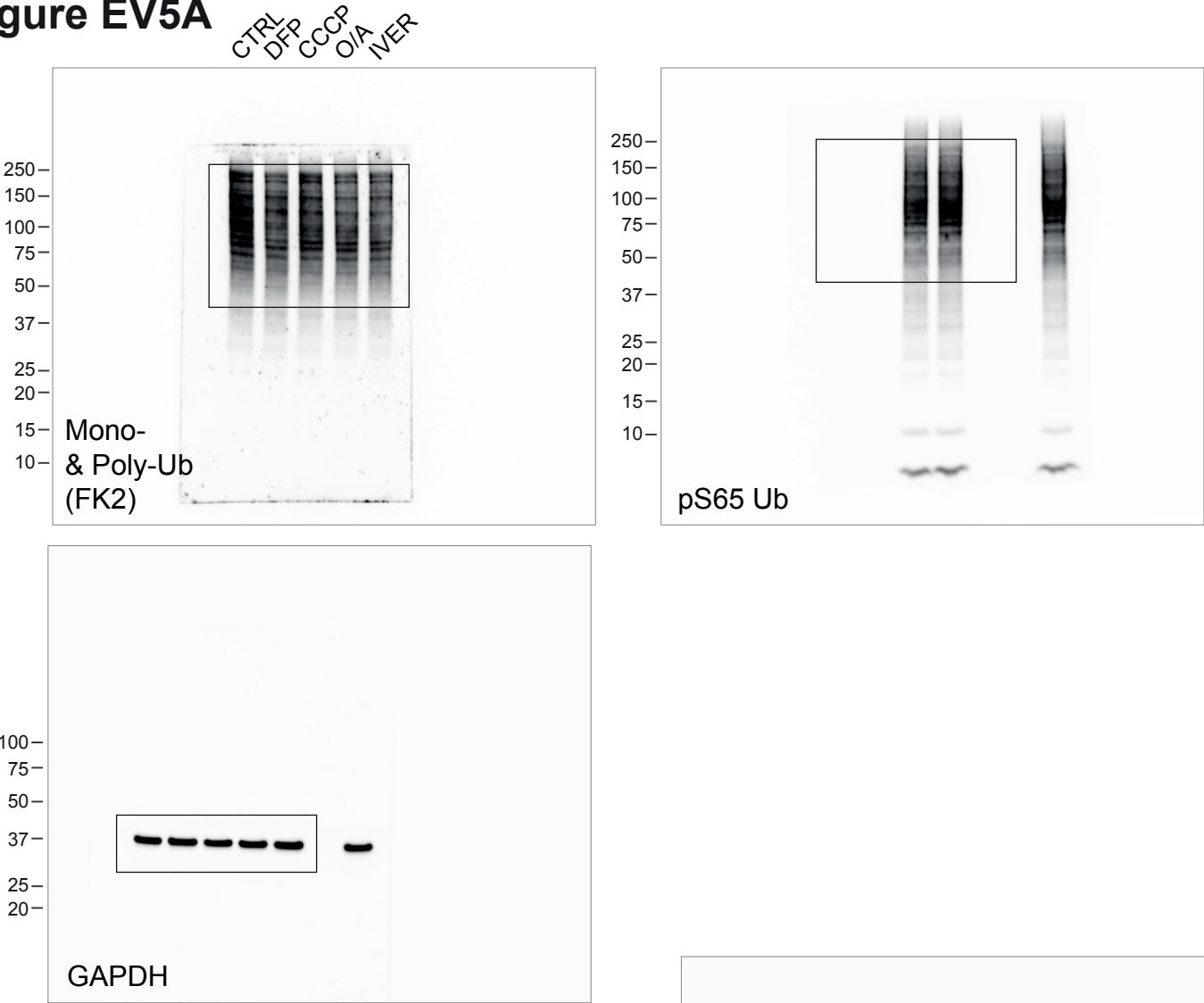

Figure EV5E

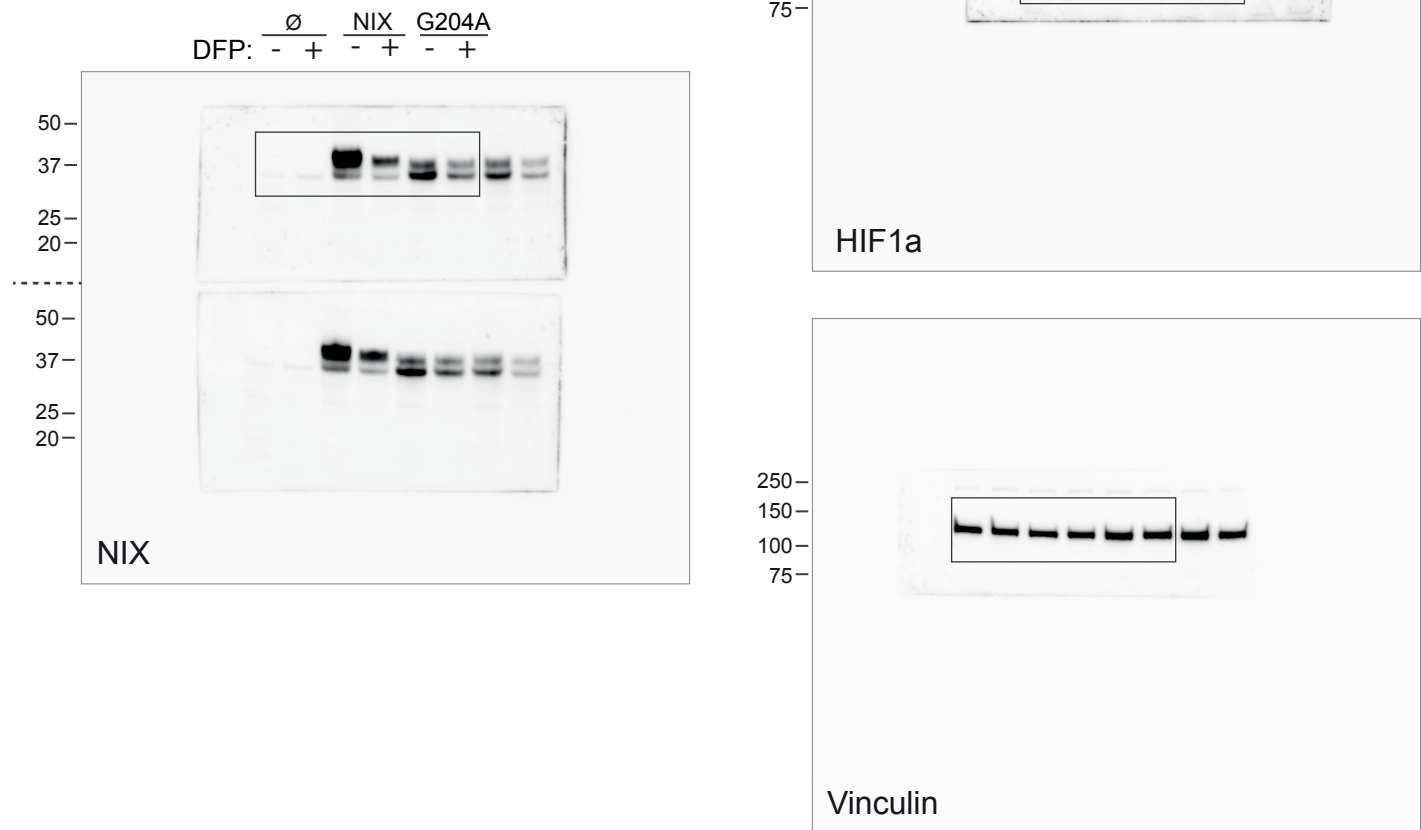

Supplement: Supplementary file 2 — Source Data for Expanded View [file EMBJ-41-e111115-s005.zip › EMBOJ-2022-111115R1-Figure_EV5_Source_Data-sd.pdf]

Figure EV6C

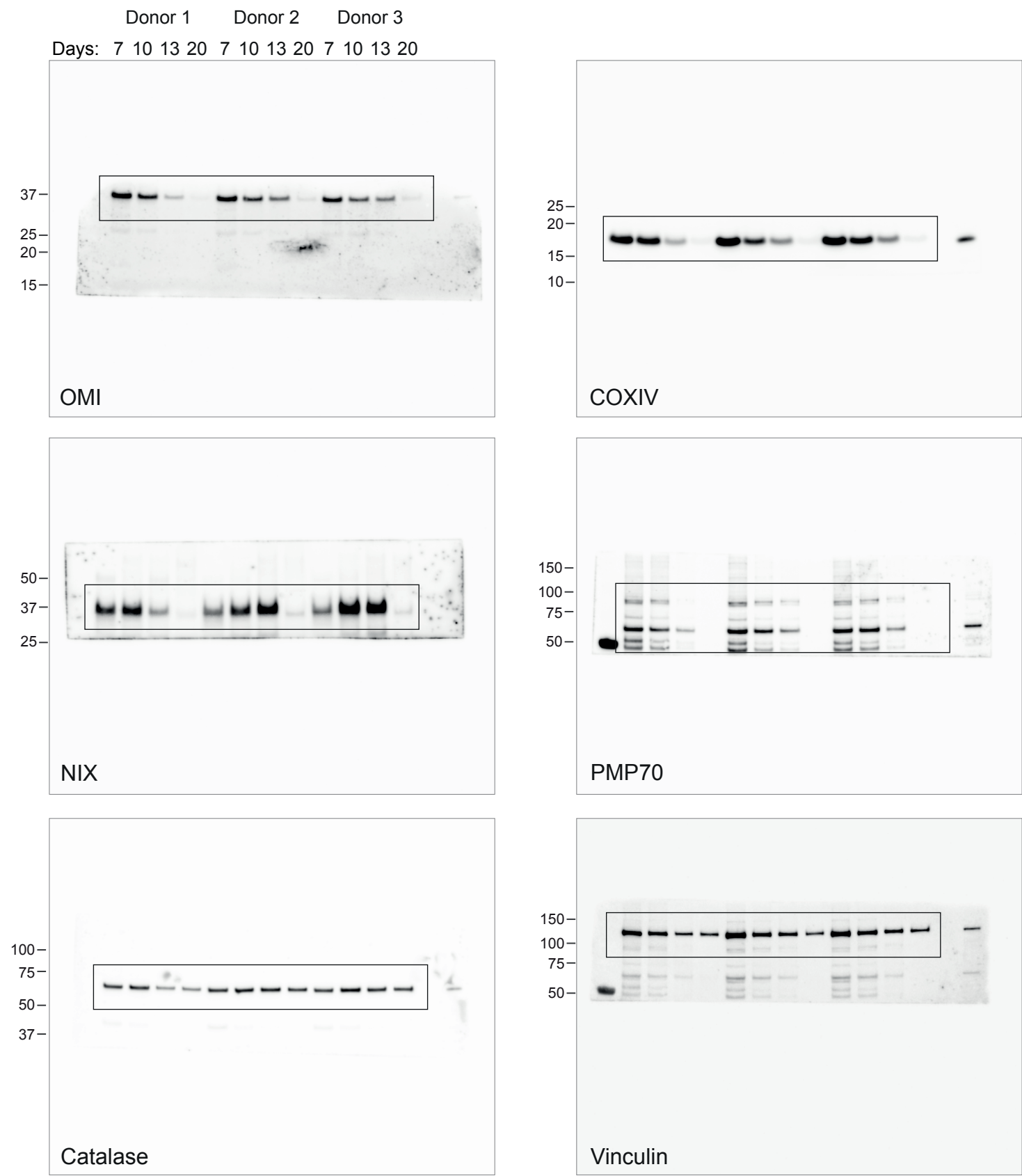

Figure EV6C

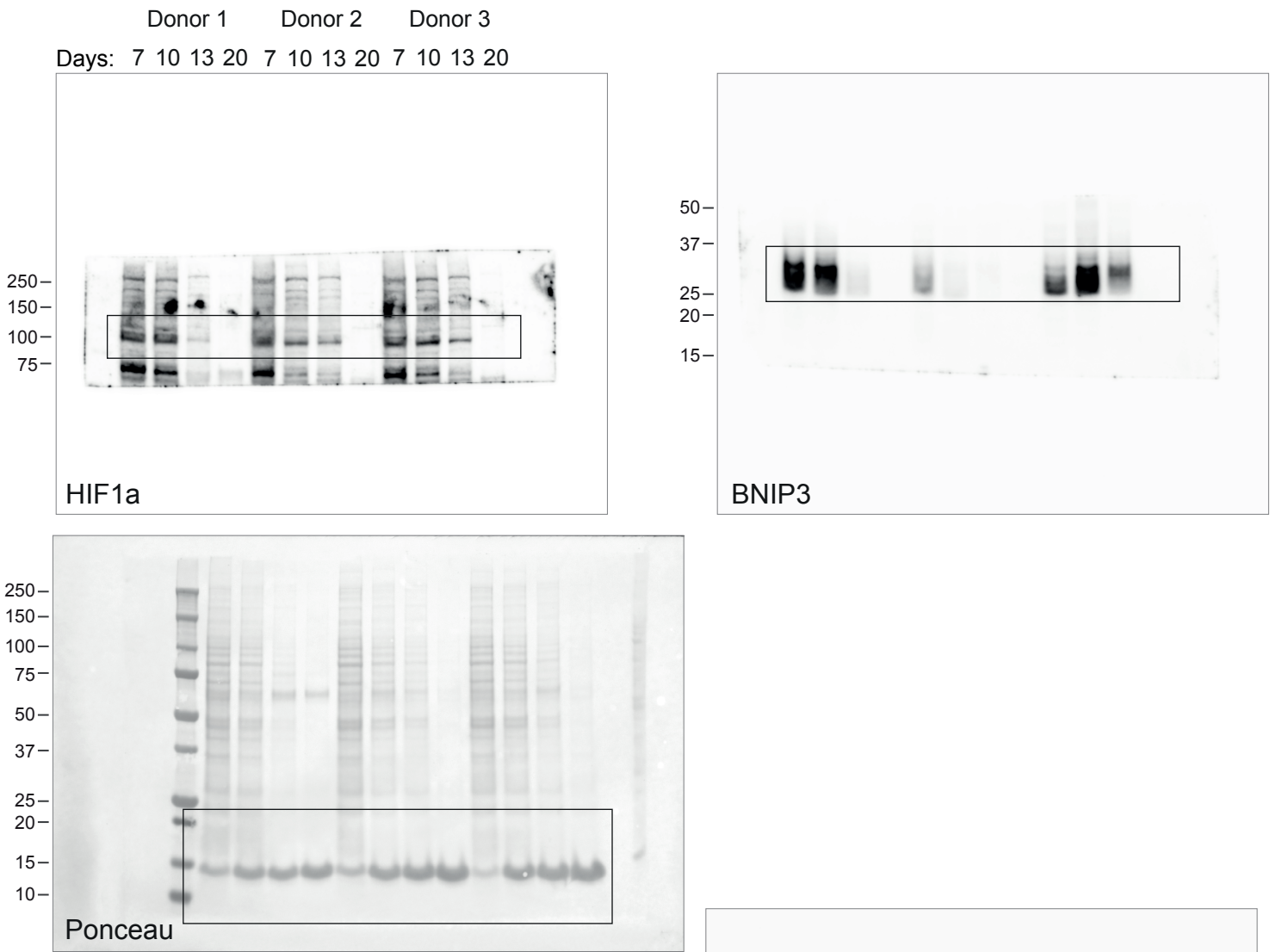

Figure EV6D

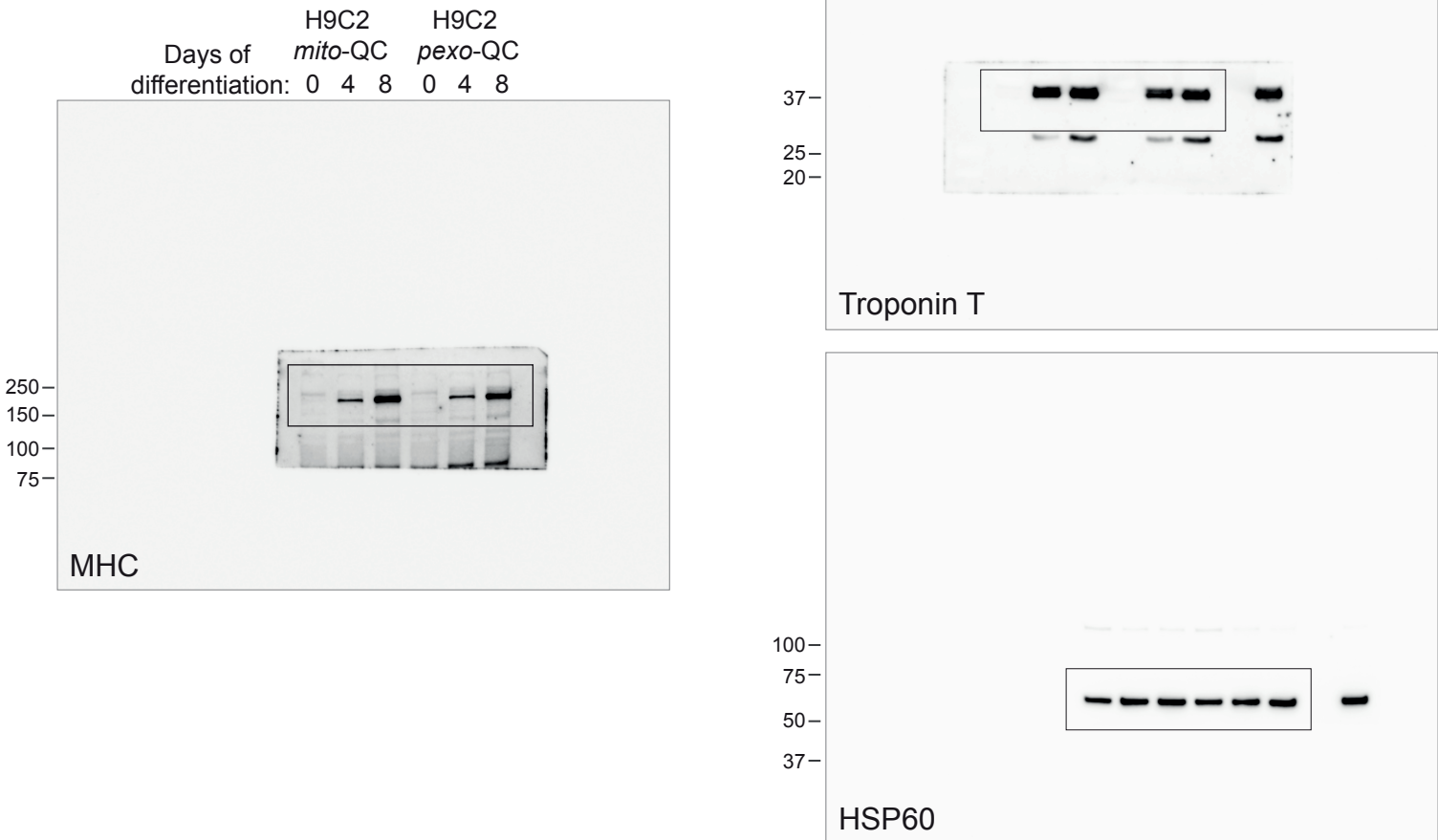

Figure EV6D

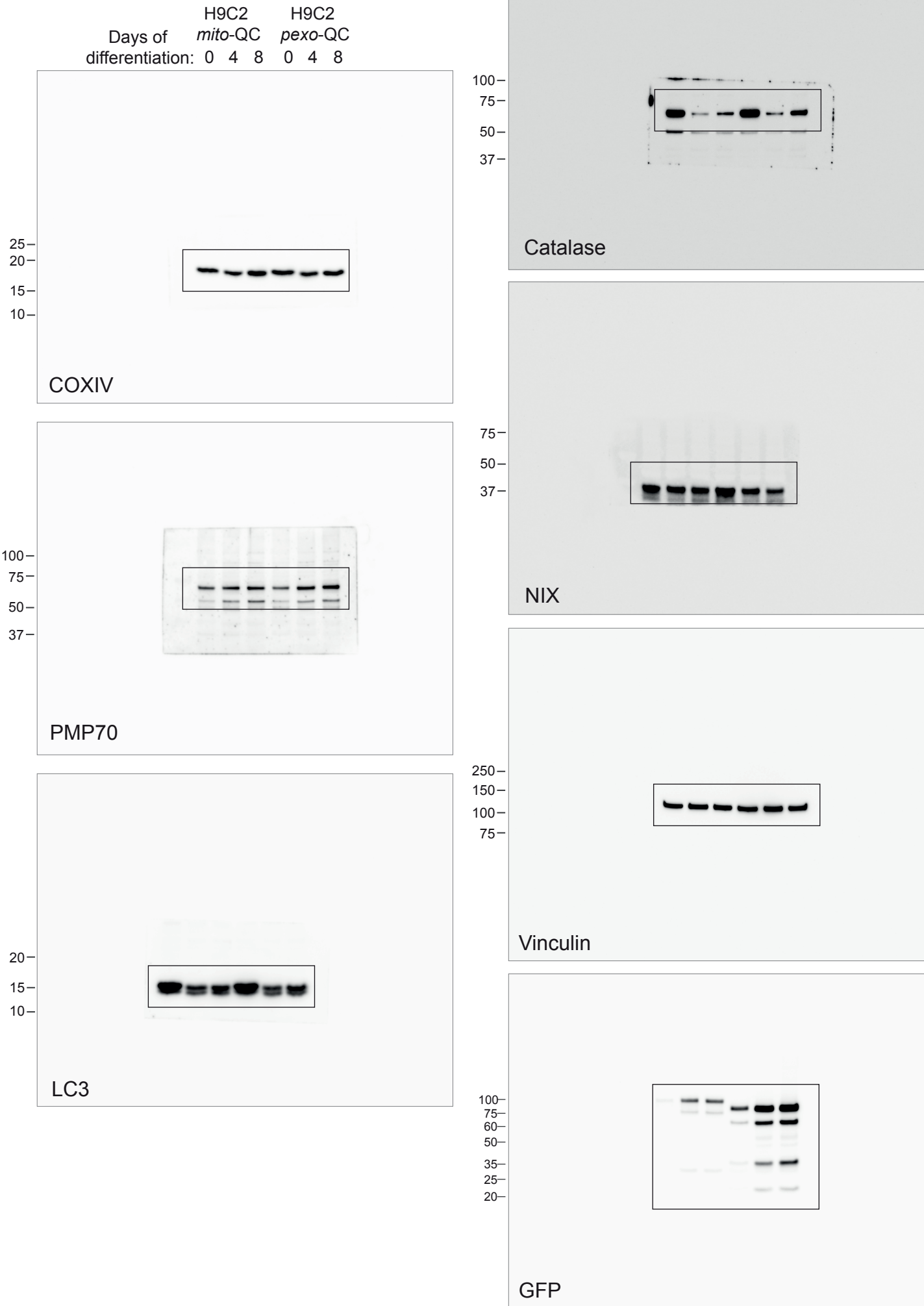

Supplement: Supplementary file 2 — Source Data for Expanded View [file EMBJ-41-e111115-s005.zip › EMBOJ-2022-111115R1-Figure_EV6_Source_Data-sd.pdf]

Figure 1A

Time of DFP (h)  
0 6 12 24 36 48 72

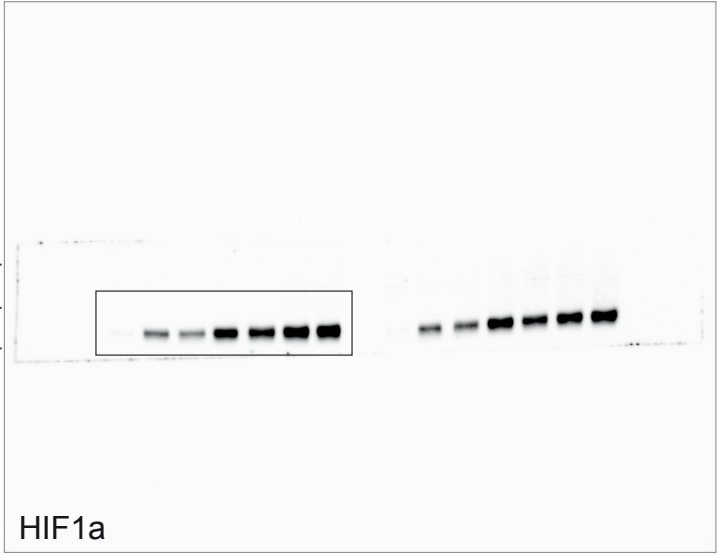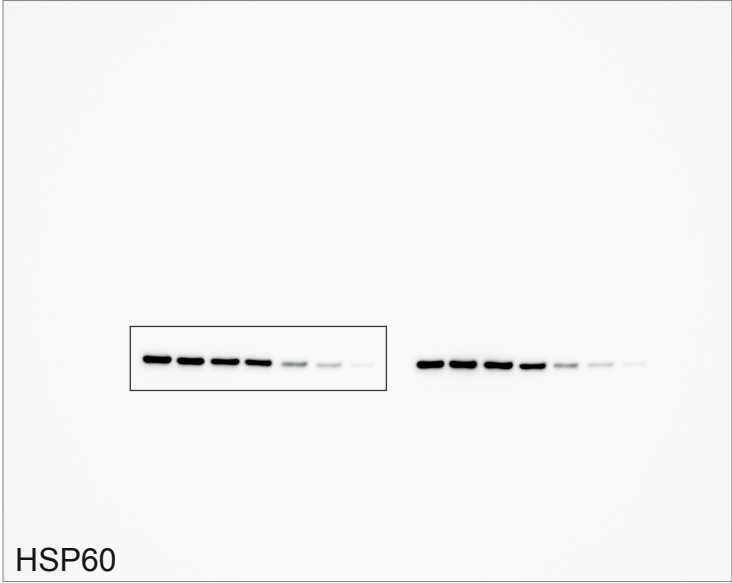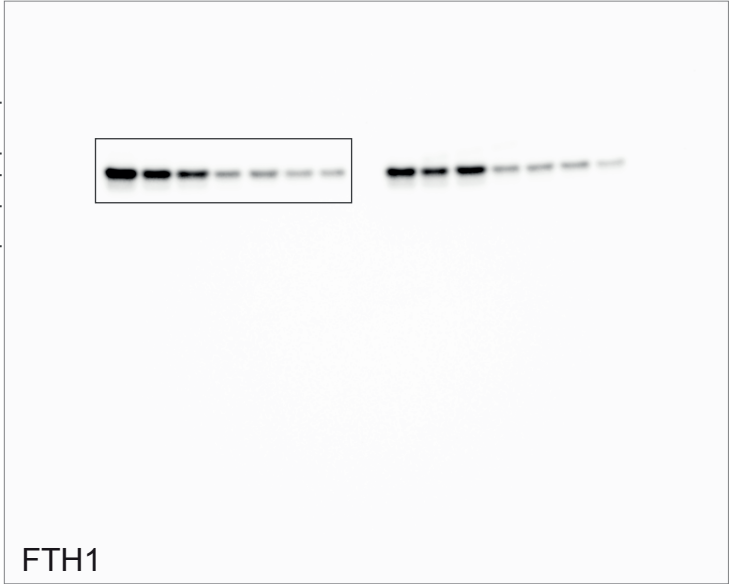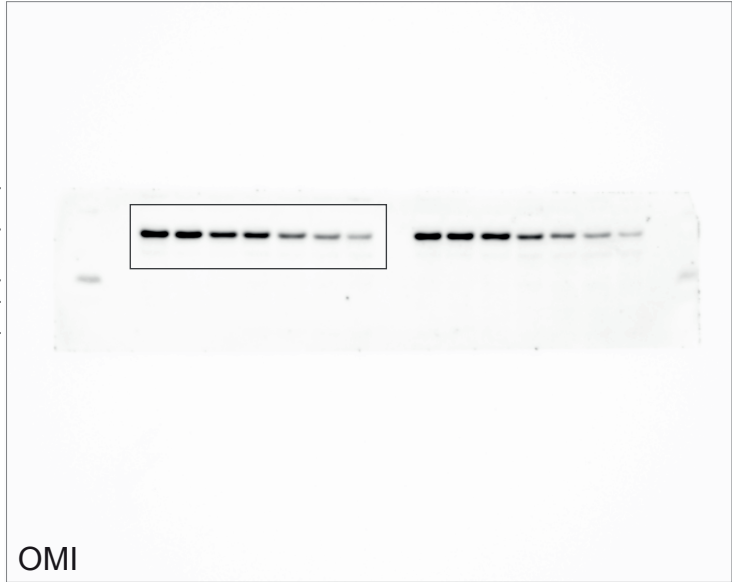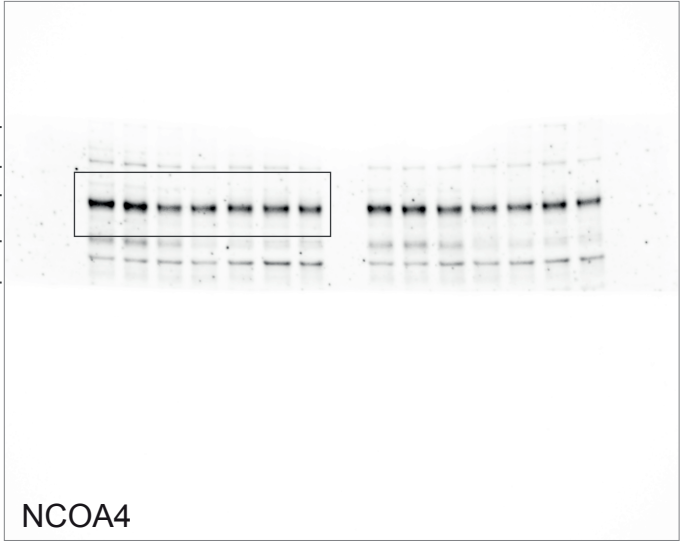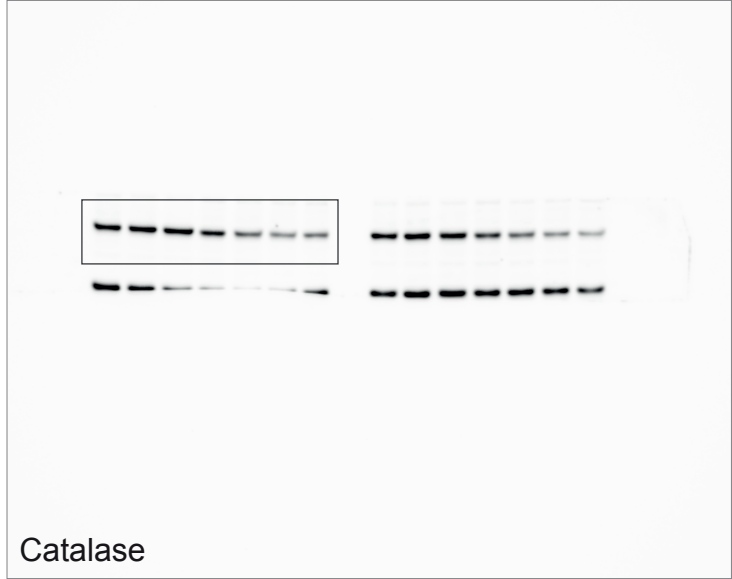

### Figure 1A

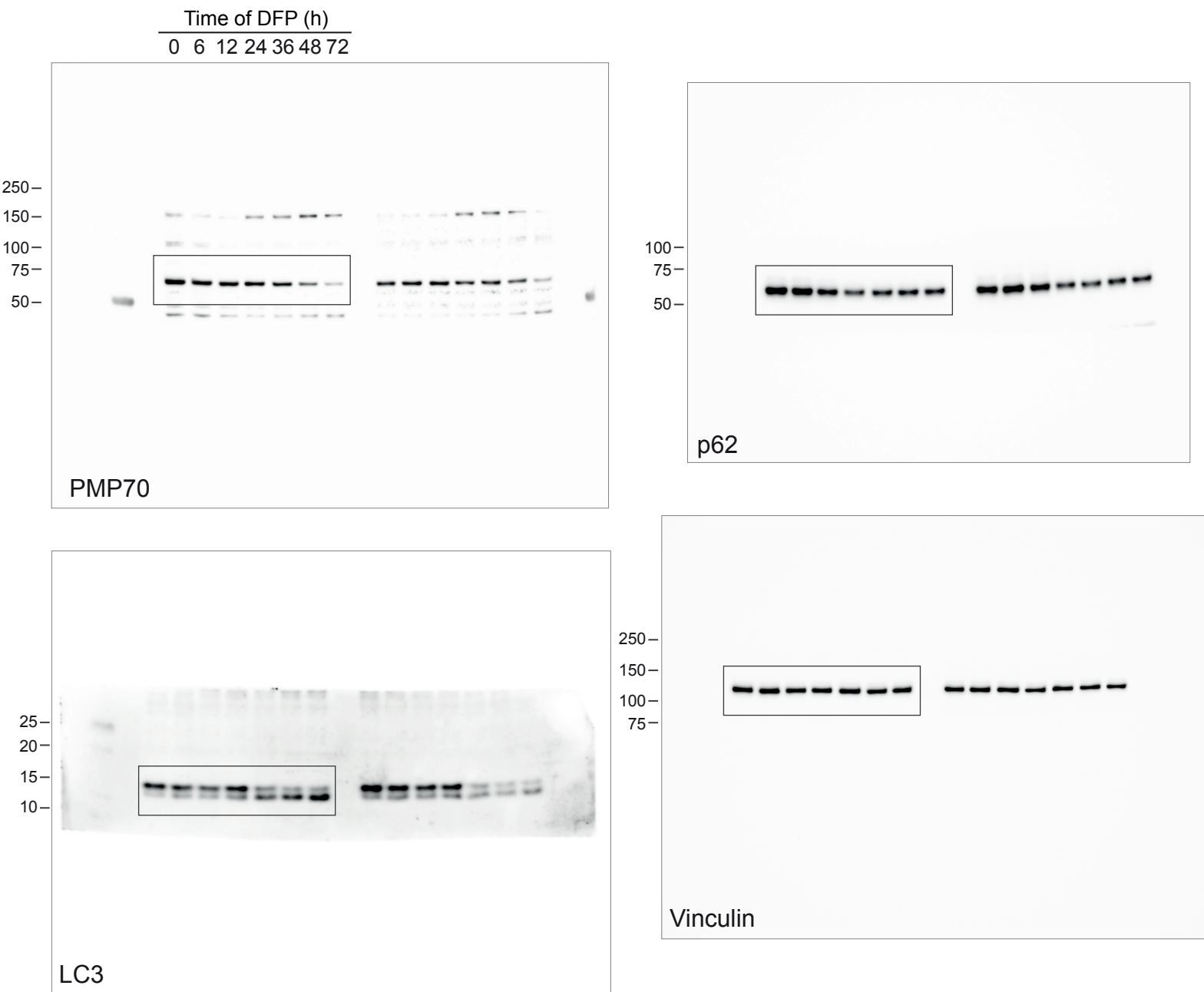

Figure 1D

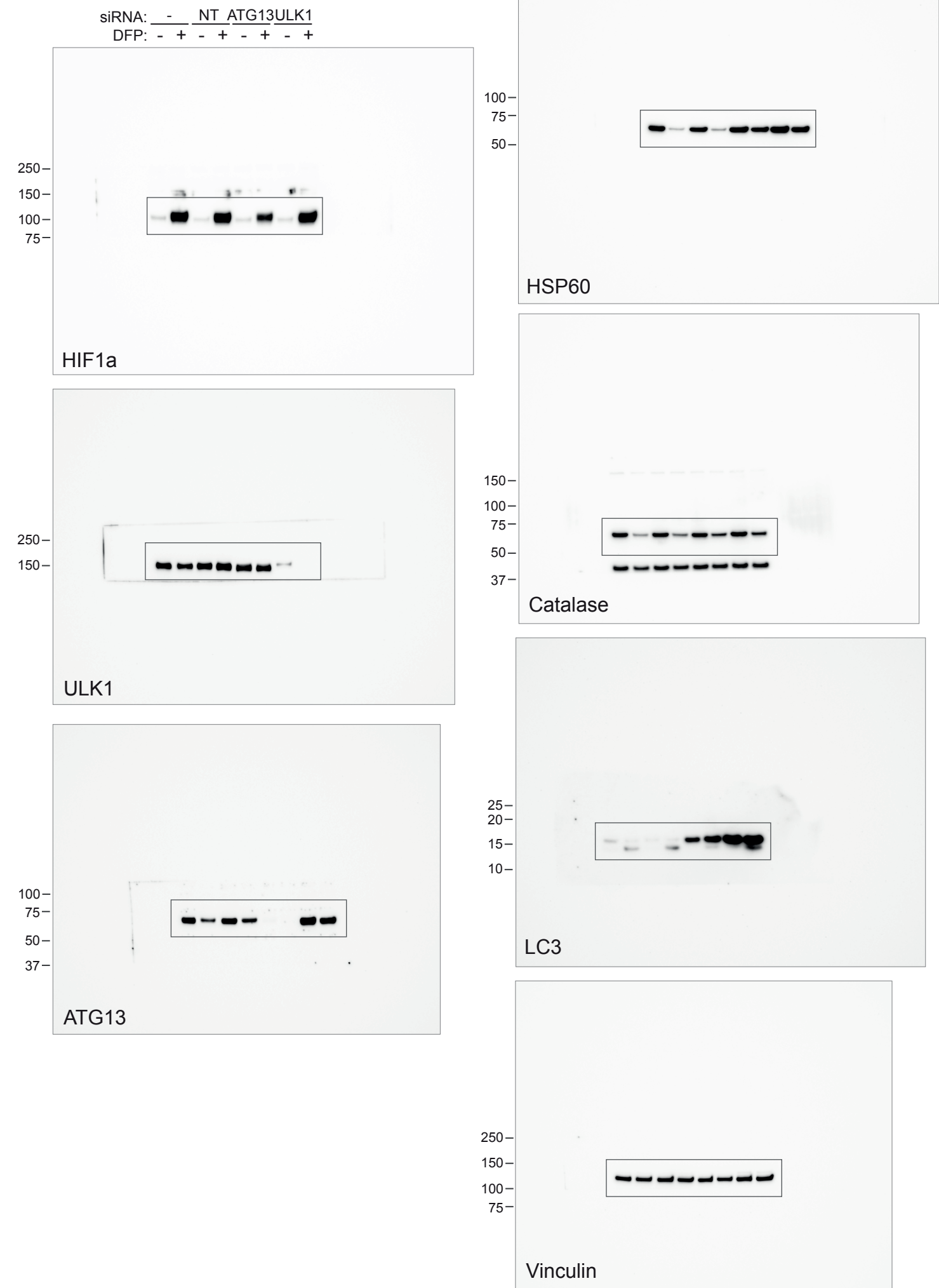

Supplement: Supplementary file 4 — Source Data for Figure 1 [file EMBJ-41-e111115-s004.pdf]

Figure 2A

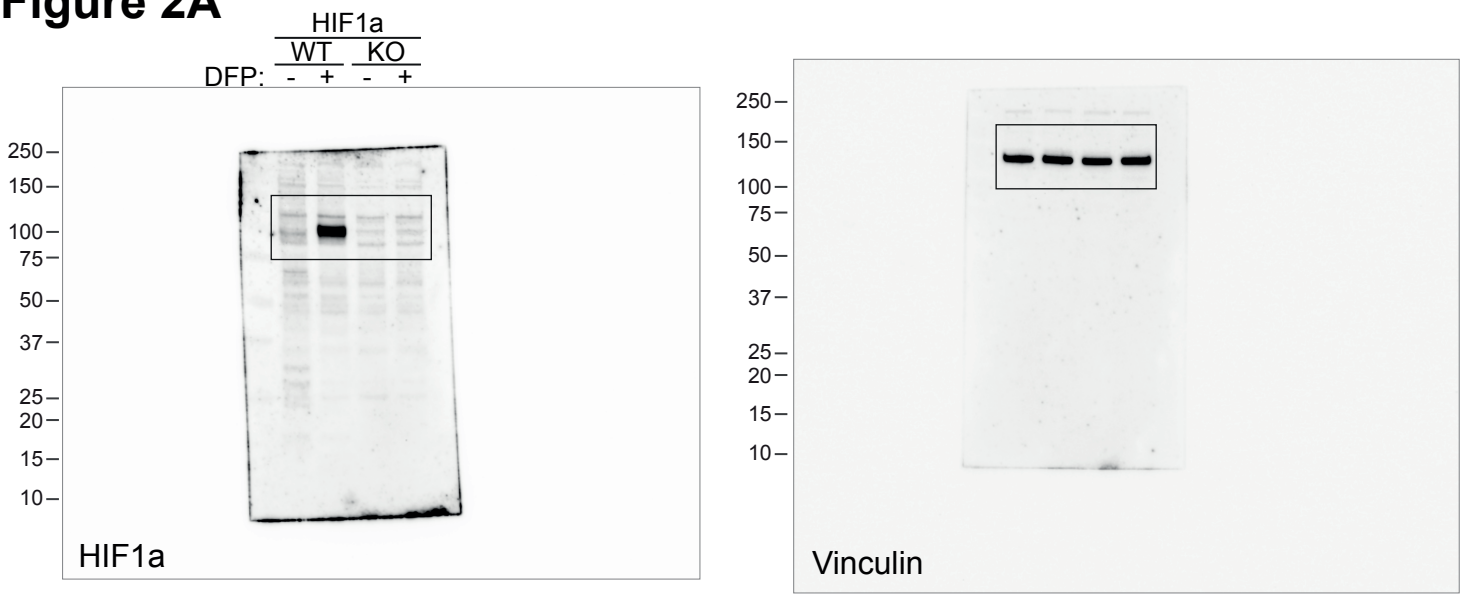

Figure 2C

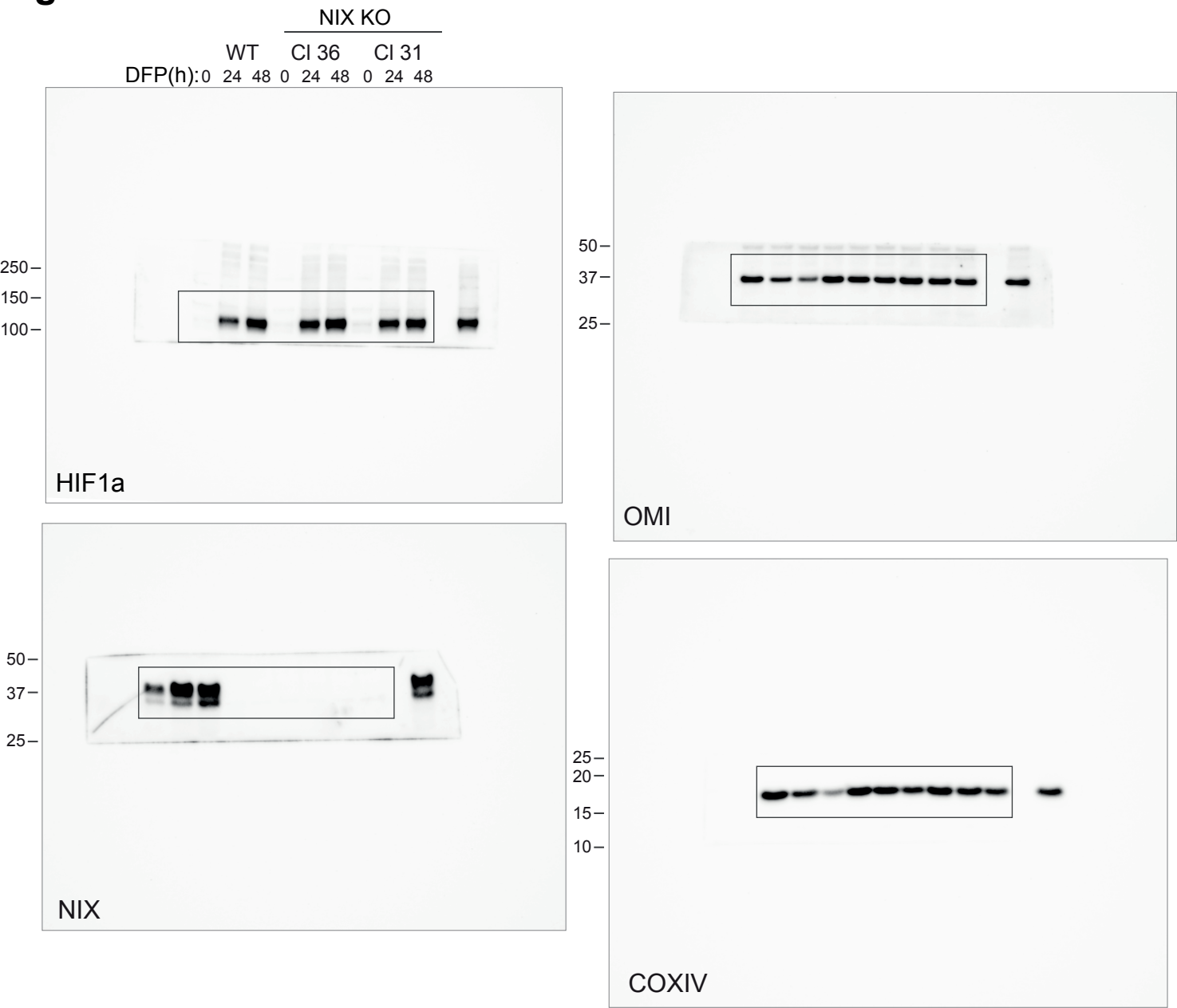

Figure 2C

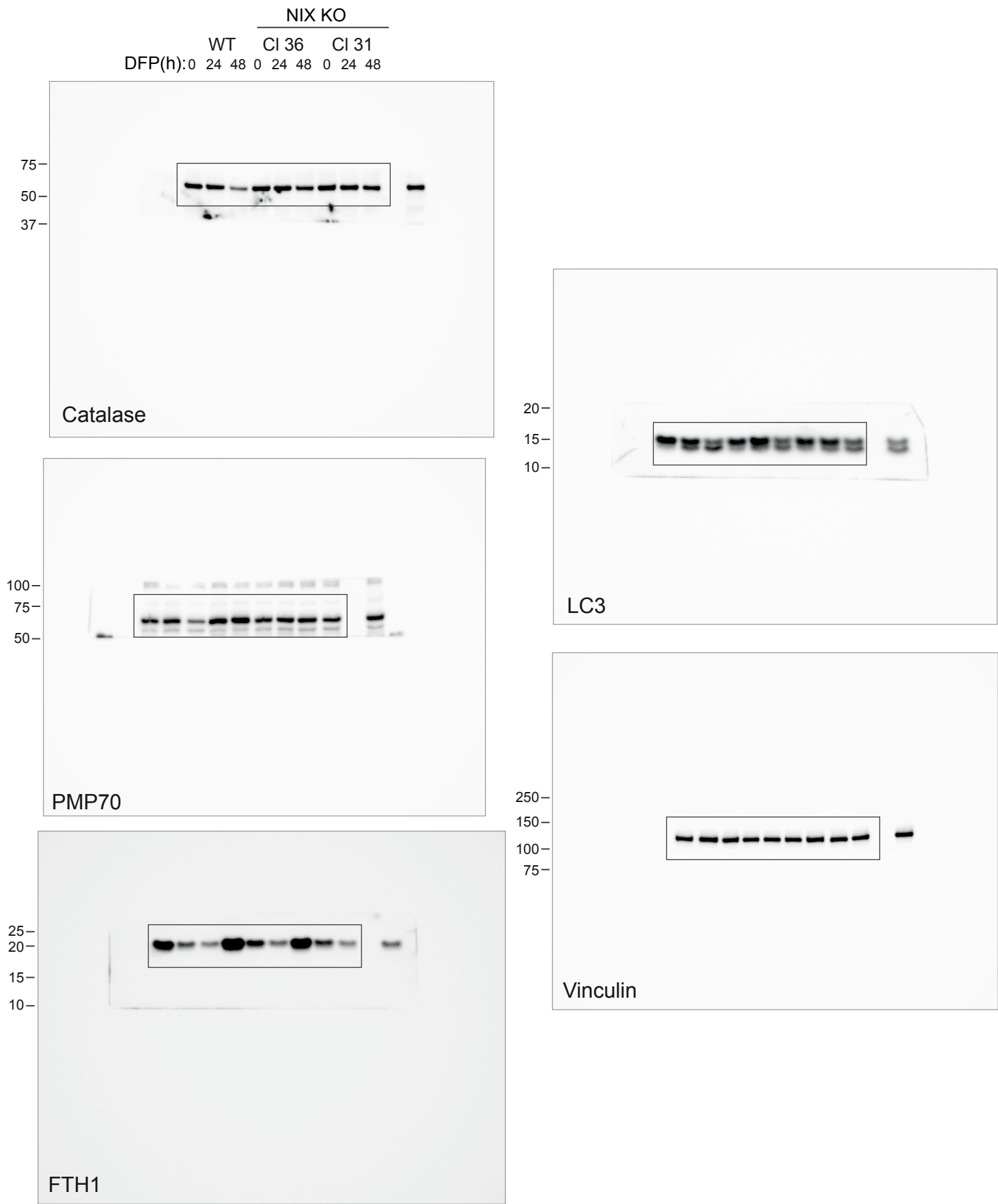

Figure 2F

WT KO NIX KO NIX  
+pBabe Ø +pBabe NIX  
DFP: - + - + - +

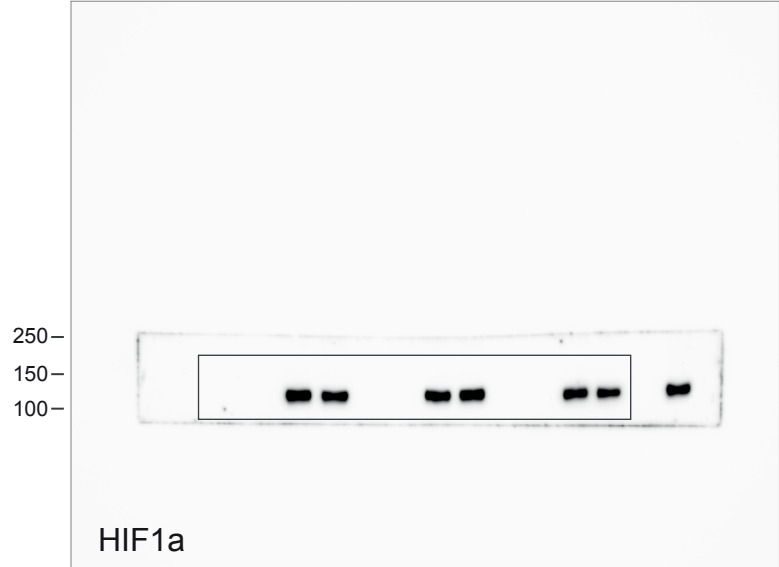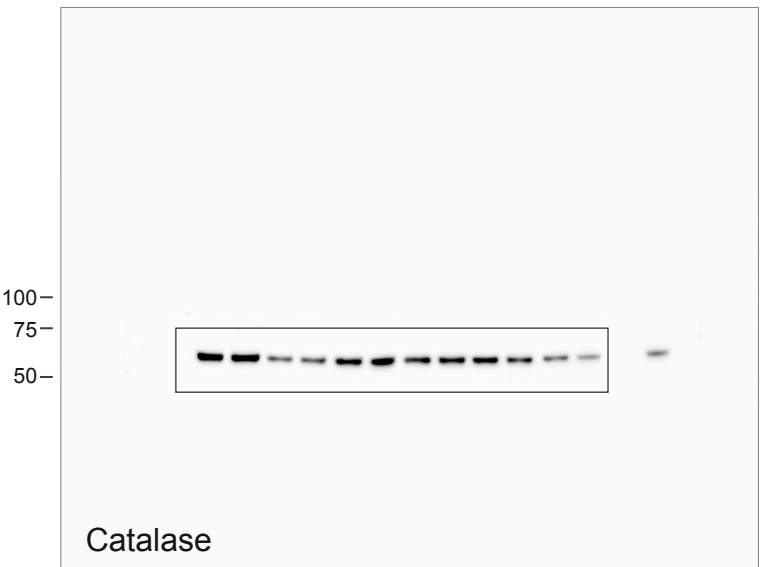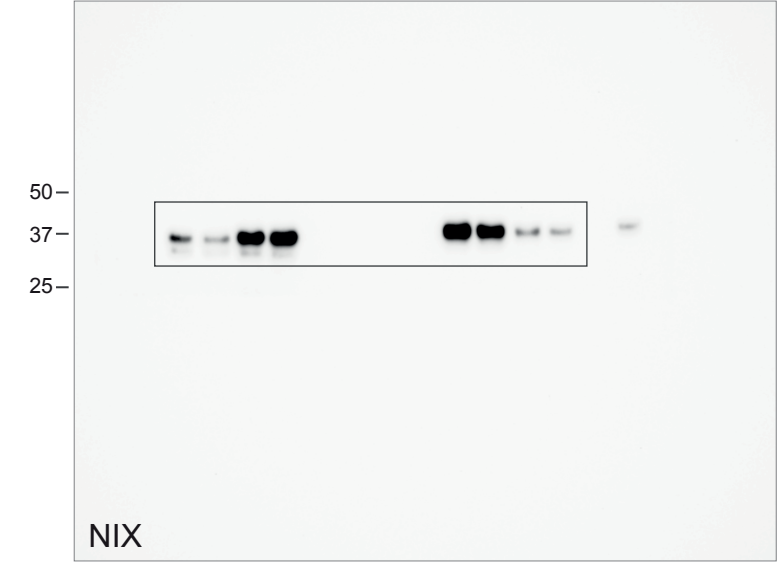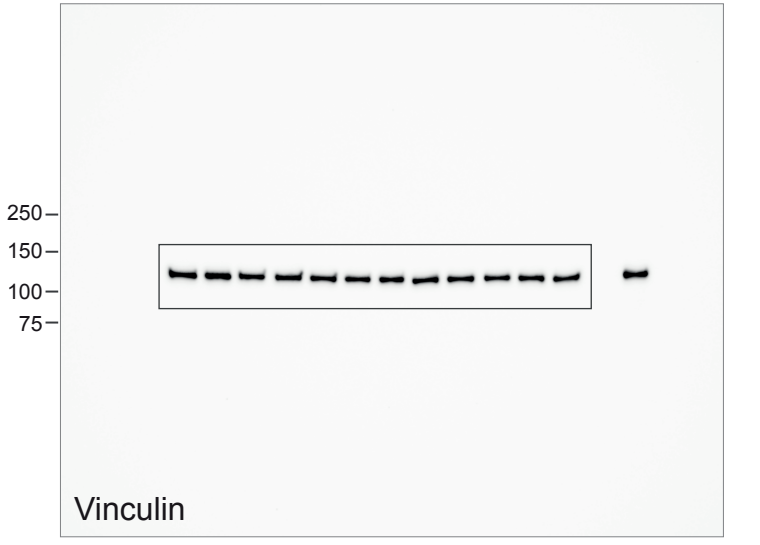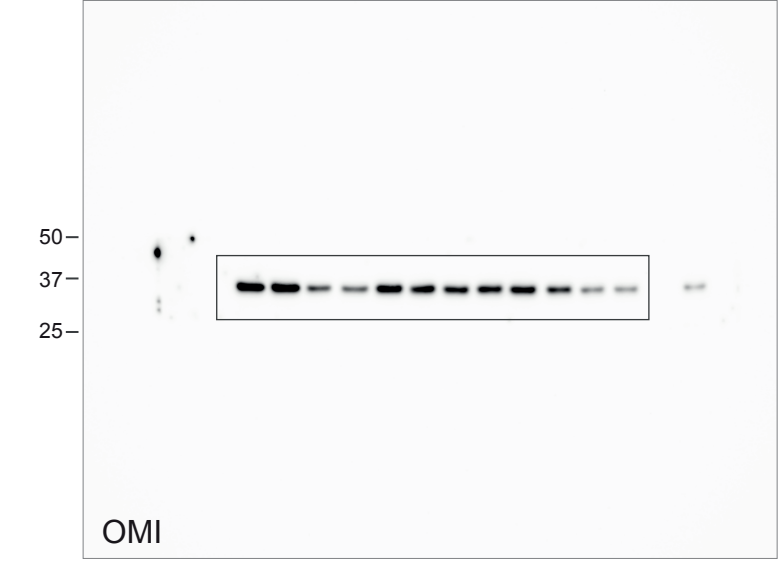

Supplement: Supplementary file 5 — Source Data for Figure 2 [file EMBJ-41-e111115-s002.pdf]

Figure 4B

|      |   | CCCP |      |
|------|---|------|------|
| DFP: |   | WT   | PRKN |
| -    | + | -    | +    |
| -    | - | -    | -    |
| -    | - | +    | +    |
| -    | - | +    | -    |
| -    | - | -    | +    |

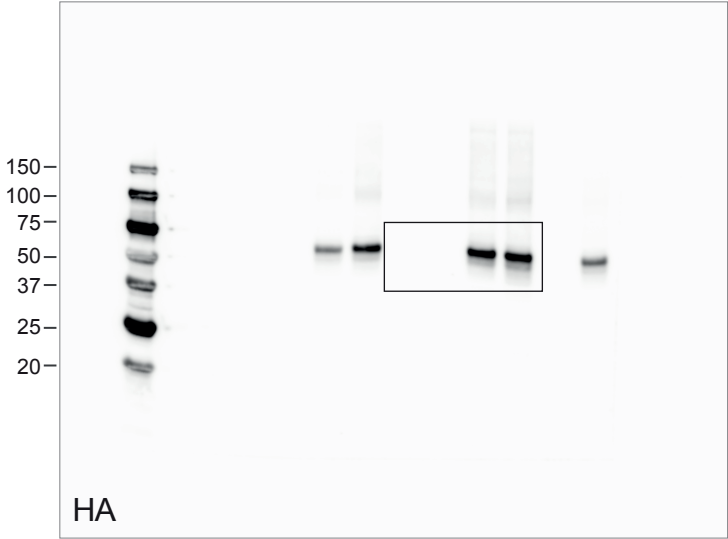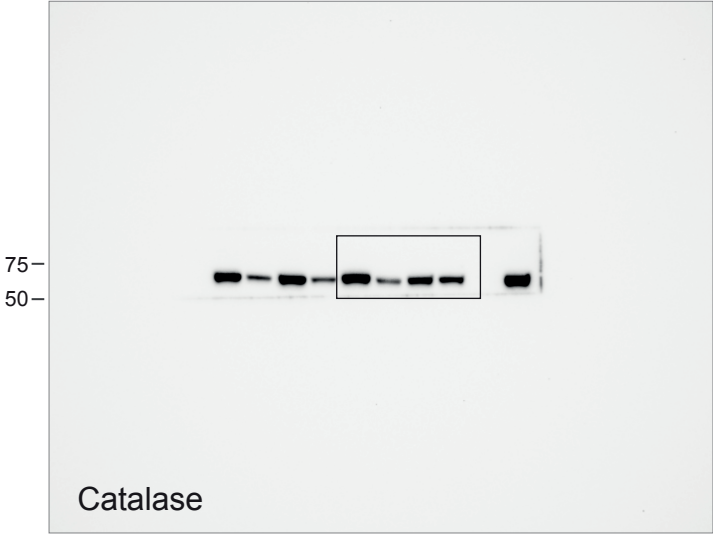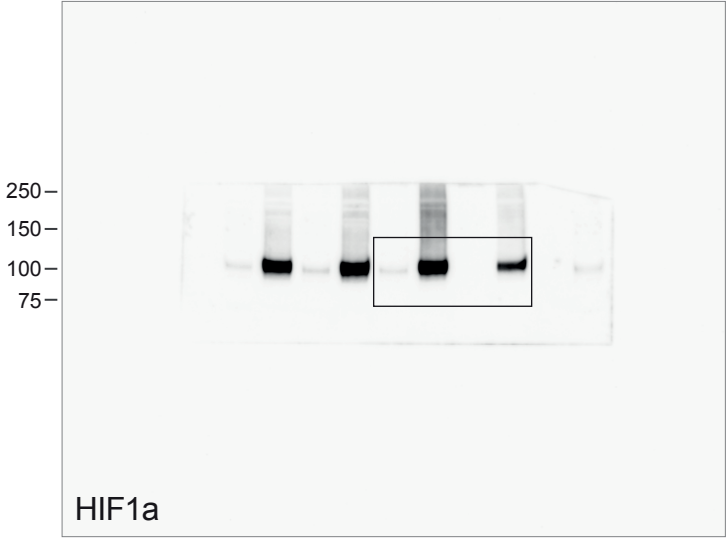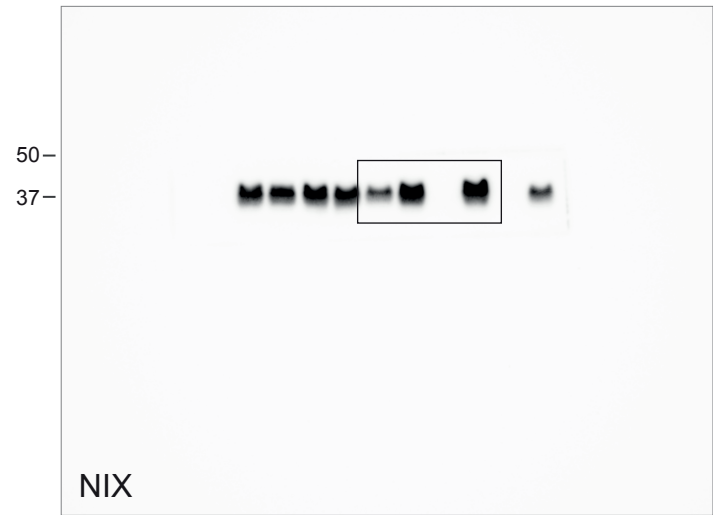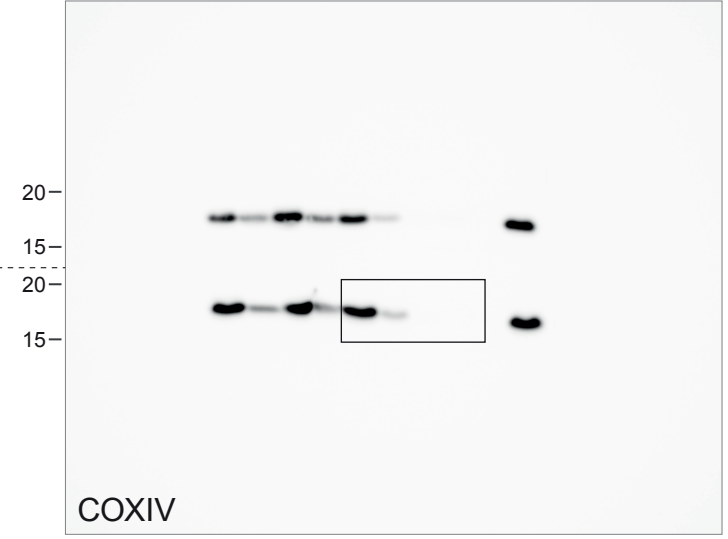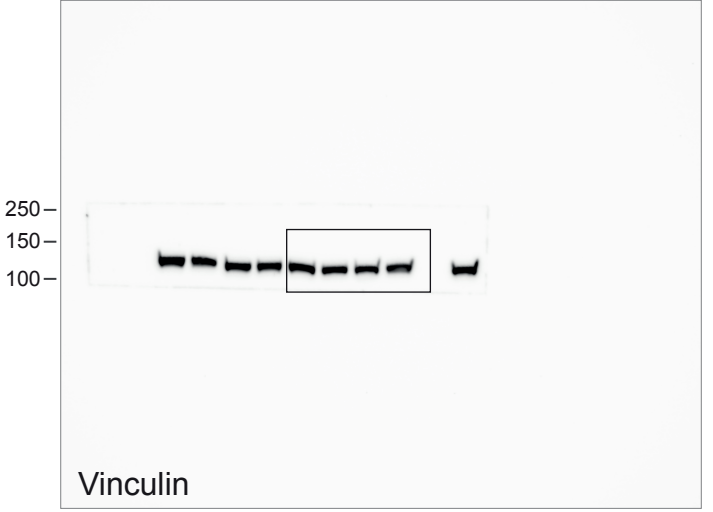

Supplement: Supplementary file 6 — Source Data for Figure 4 [file EMBJ-41-e111115-s008.pdf]

Figure 5B

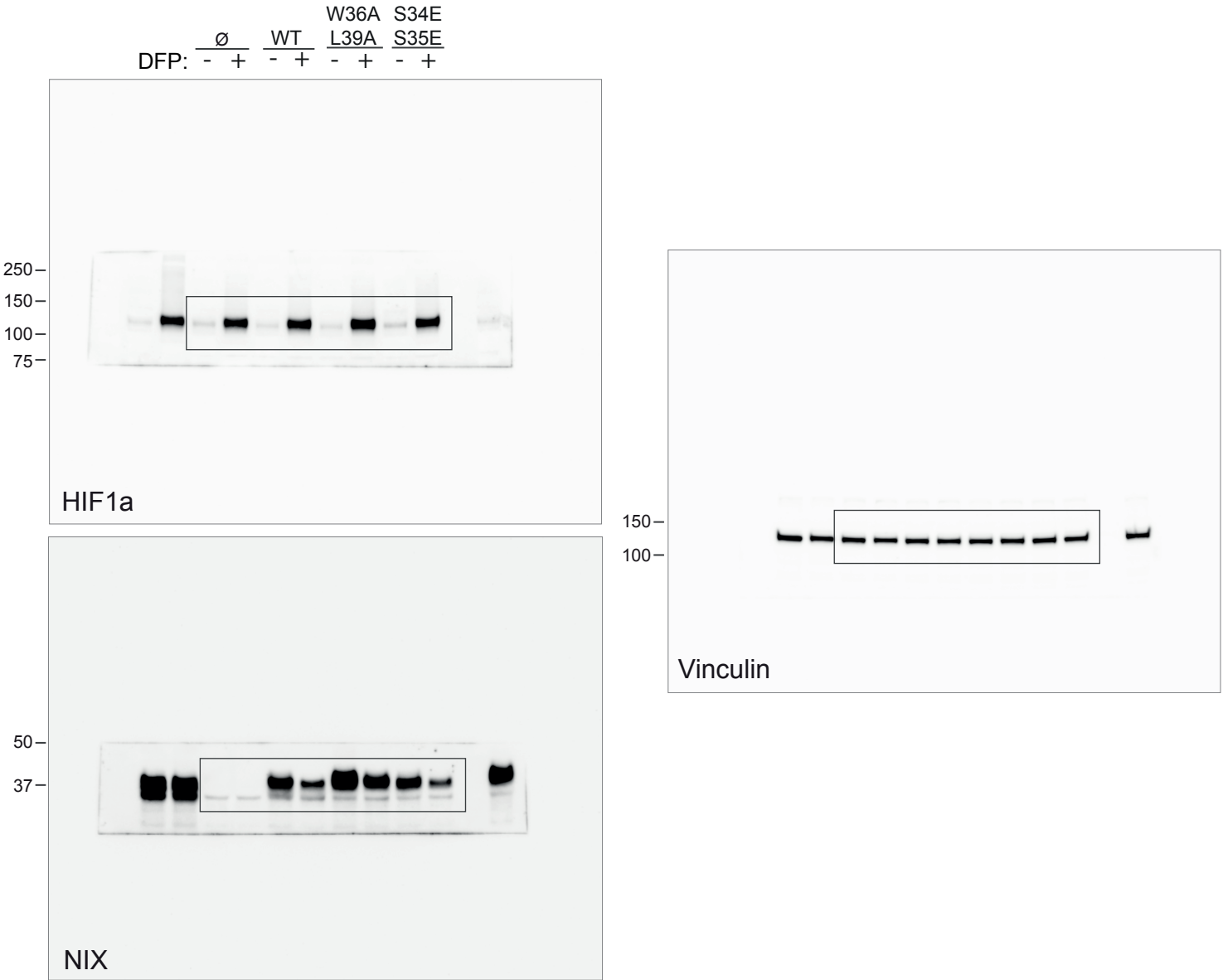

Supplement: Supplementary file 7 — Source Data for Figure 5 [file EMBJ-41-e111115-s007.pdf]
