# Supplementary material for: BNIP3L/NIX regulates both mitophagy and pexophagy
Source: EMBO J. 2022 Oct 10;41(24):e111115. doi: 10.15252/embj.2022111115 (PMC9753467; doi:10.15252/embj.2022111115)
Supplement: Supplementary file 3 — PDF+ [file EMBJ-41-e111115-s003.pdf]

Article

SOURCE DATA TRANSPARENT PROCESS OPEN ACCESS

THE EMBO JOURNAL

BNIP3L/NIX regulates both mitophagy and pexophagy

Léa P Wilhelm[1], Juan Zapata-Muñoz[2], Beatriz Villarejo-Zori[2], Stephanie Pellegrin[3,4], Catarina Martins Freire[3], Ashley M Toye[3,4], Patricia Boya[2] & Ian G Ganley[1,*]

Abstract

Mitochondria and peroxisomes are closely related metabolic organelles, both in terms of origin and in terms of function. Mitochondria and peroxisomes can also be turned over by autophagy, in processes termed mitophagy and pexophagy, respectively. However, despite their close relationship, it is not known if both organelles are turned over under similar conditions, and if so, how this might be coordinated molecularly. Here, we find that multiple selective autophagy pathways are activated upon iron chelation and show that mitophagy and pexophagy occur in a BNIP3L/NIX-dependent manner. We reveal that the outer mitochondrial membrane-anchored NIX protein, previously described as a mitophagy receptor, also independently localises to peroxisomes and drives pexophagy. We show this process happens *in vivo*, with mouse tissue that lacks NIX having a higher peroxisomal content. We further show that pexophagy is stimulated under the same physiological conditions that activate mitophagy, including cardiomyocyte and erythrocyte differentiation. Taken together, our work uncovers a dual role for NIX, not only in mitophagy but also in pexophagy, thus illustrating the interconnection between selective autophagy pathways.

Keywords autophagy; mitochondria; mitophagy; peroxisomes; pexophagy
Subject Category Autophagy & Cell Death
DOI 10.15252/embj.2022111115 | Received 9 March 2022 | Revised 14 September 2022 | Accepted 16 September 2022 | Published online 10 October 2022
The EMBO Journal (2022) 41: e111115

Introduction

Macroautophagy, a degradative lysosomal pathway herein simplified to autophagy, was originally thought to be a nonselective process, resulting in bulk turnover of cytosolic components in a seemingly random manner (Mizushima, 2018). However, spurred on by the identification of autophagy receptor proteins, we now know that autophagy can be highly selective in terms of what can be incorporated into the forming autophagosome, be it protein aggregates, organelles or intracellular pathogens (Kirkin, 2020). It is less clear though how these specific autophagy pathways relate to each other. For example, are specific cargoes always turned over independently and in isolation, or does turnover of one specific cargo influence the autophagy of another? One of the most intensively studied specific autophagy pathways is mitophagy, or the autophagy of mitochondria. Multiple mitophagy pathways have now been identified that involve tagging the mitochondria with an "eat-me" signal (Rodger *et al*, 2018). This signal can involve ubiquitylation of outer mitochondrial membrane (OMM) proteins, which recruits ubiquitin-binding autophagy receptors, such as p62/SQSTM1, OPTN or NDP52. These can then bind directly to the autophagy machinery via their LC3 interacting regions (LIRs) or FIP200 binding motifs. Alternatively, certain OMM proteins can be upregulated and act directly as autophagy receptors, such as NIX/BNIP3L and FUNDC1, thus bypassing the need for ubiquitylation (Montava-Garriga & Ganley, 2020; Teresak *et al*, 2022).

The need to undergo mitophagy is clear, as mitochondria are key metabolic hubs and their quality control is essential for normal cellular function: mitochondrial dysfunction is a feature of many diseases, including neurodegenerative disorders and cancer. While there are multiple mitochondrial quality control pathways, mitophagy is thought to play a key role, especially in limiting the production of damaging reactive oxygen species (ROS) that can arise from dysfunctional mitochondria (Ng *et al*, 2021). This is highlighted by the fact that certain genes mutated in forms of hereditary Parkinson's disease can regulate mitophagy (e.g. PINK1, Parkin and LRRK2; Narendra *et al*, 2008; Matsuda *et al*, 2010; Narendra *et al*, 2010b; Singh *et al*, 2021; Singh & Ganley, 2021).

Peroxisomes are also key metabolic organelles performing roles in fatty acid metabolism and ROS scavenging (Tabak *et al*, 1999; Smith & Aitchison, 2013; Mast *et al*, 2020). As with mitochondria, peroxisomal dysfunction can lead to debilitating diseases such as X-linked adrenoleukodystrophy and Zellweger syndrome (Braverman *et al*, 2016; Waterham *et al*, 2016; Wangler *et al*, 2018). Additionally, peroxisomes are closely connected to mitochondria; not only can they communicate directly with mitochondria through membrane contact sites (Schrader *et al*, 2020) and mitochondrial-derived vesicles (Neuspiel *et al*, 2008) but peroxisomes also require

1 MRC Protein Phosphorylation and Ubiquitylation Unit, University of Dundee, Dundee, UK
2 Department of Cellular and Molecular Biology, Margarita Salas Center for Biological Research, CSIC, Madrid, Spain
3 School of Biochemistry, Biomedical Sciences Building, University Walk, Bristol, UK
4 National Institute for Health Research (NIHR) Blood and Transplant Research Unit in Red Blood Cell Products, University of Bristol, Bristol, UK
*Corresponding author. Tel: +44(0)1382 388905; E-mail: i.ganley@dundee.ac.uk

© 2022 The Authors. Published under the terms of the CC BY 4.0 license.

mitochondria for biogenesis (Sugiura *et al*, 2017). Peroxisomes can also be targeted by autophagy, termed pexophagy, but in mammalian cells at least, this process is less well-understood compared with mitophagy. As both mitochondria and peroxisomes can release ROS when damaged, conceivably under the same conditions, it is possible that both these organelles could contribute to disease pathology at the same time. Given this, and that they are closely related in origins and function, we explored whether their autophagy could also be linked. Using iron chelation, a potent inducer of mitophagy (Allen *et al*, 2013), as well as hypoxia, we show that mitochondria and peroxisomes are targeted by autophagy and that both these processes require the autophagy receptor NIX.

Results

Iron chelation induces sequential autophagy of organelles

We previously identified multiple inducers of mitophagy that operate through distinct signalling pathways. The most potent mitophagy inducers were iron chelators, in particular deferiprone (DFP), which mimic hypoxic conditions through stabilisation of the oxygen-sensitive transcription factor HIF1α (Allen *et al*, 2013; Zhao *et al*, 2020). Interestingly, other studies have shown that pexophagy and the selective autophagy of ferritin (ferritinophagy) can also be induced by iron depletion (Asano *et al*, 2011; Mancias *et al*, 2014; Jo *et al*, 2015; Jin *et al*, 2016). However, it is not clear how these selective autophagy pathways are orchestrated and whether there is a direct mechanistic link between them. To explore this, we used ARPE-19 cells, a human-derived retinal pigment epithelial line that we have previously shown is very amenable to mitophagy induction (Montava-Garriga *et al*, 2020; Rosignol *et al*, 2020). Cells were treated with DFP for the indicated time, and the levels of ferritin, mitochondrial or peroxisomal markers were monitored by immunoblot analysis, with a decrease in levels indicative of increased ferritinophagy, mitophagy and pexophagy, respectively (Fig 1A). The first proteins showing increased degradation upon DFP treatment were ferritin heavy chain (FTH1) and NCOA4, a cargo receptor mediating ferritinophagy (Mancias *et al*, 2014). A significant decrease in FTH1 level was observed as early as 6 h after DFP treatment indicating that ferritinophagy is the first selective form of autophagy to be activated. This possibly allows rapid release of the stored pool of iron in an attempt to restore iron homeostasis. The second set of proteins to decrease were the mitochondrially localised HSP60 and OMI, demonstrating that mitophagy follows ferritinophagy with significantly decreased levels occurring after 36 h of DFP. Finally, pexophagy appeared to be significantly induced following 48 h of DFP treatment, as shown by the reduced level of the peroxisomal proteins catalase, PMP70, PEX5 and PEX19 (Figs 1A and EV1A). These results were also confirmed in two other cell lines, HeLa and SH-SY5Y, and with another iron chelator, phenanthroline (Phen; Mauro-Lizcano *et al*, 2015), signifying that this phenomenon is not specific to ARPE-19 cells or DFP (Fig EV1B and C). Additionally, given that hypoxia can stimulate a mitophagy pathway similar to that of DFP (Zhao *et al*, 2020), we next analysed pexophagy under low oxygen conditions (Fig EV1D and E). As with mitophagy, hypoxia conditions also stimulate pexophagy.

To confirm mitochondrial autophagy, we monitored mitophagy after DFP treatment using our previously established and well-characterised *mito*-QC assay (Allen *et al*, 2013; Montava-Garriga *et al*, 2020). This assay consists of cells expressing a tandem mCherry-GFP tag fused to the outer mitochondrial membrane localisation peptide of FIS1 protein (residues 101–152). GFP is quenched by low pH; therefore, mitochondria that are delivered to lysosomes (referred as mitolysosomes) appear as "red-only" puncta that can be easily quantified. As expected, ARPE-19 *mito*-QC cells showed a robust increase in DFP-stimulated mitolysosomes in a time-dependent manner (Fig 1B).

To confirm that pexophagy is also induced by DFP, we generated a pexophagy reporter (referred to as *pexo*-QC here for consistency) that operates under the same principles as *mito*-QC. Here, we attached the tandem mCherry-GFP tag to the peroxisomal matrix targeting sequence (SKL), which is based on previously generated reporter systems (Deosaran *et al*, 2013; Marcassa *et al*, 2018). As expected, the *pexo*-QC reporter completely colocalized with catalase and PMP70, demonstrating that it is efficiently targeted to peroxisomes (Fig EV1F). Moreover, the delivery of peroxisomes to lysosomes (to form pexolysosomes) is highlighted by the colocalisation of the lysosomal marker LAMP1 with the "red-only" puncta (Fig EV1G). As with the Western blot data, ARPE-19 pexophagy reporter cells (ARPE-19 *pexo*-QC) significantly increased the number of pexolysosomes after 48 h of DFP treatment (Fig 1C).

These results confirmed that DFP induces both mitophagy and pexophagy, with mitophagy preceding pexophagy. Unfortunately, we were not successful in generating a similar ferritinophagy reporter, probably due to the small size of the ferritin protein. We thus focussed our work on mitophagy and pexophagy going forward.

Activation of the ULK1 complex, comprising ULK1 kinase, ATG13, FIP200 and ATG101, is considered a key early event in autophagy induction (Zachari & Ganley, 2017). As this complex has also been implicated in mitophagy (Egan *et al*, 2011; Wu *et al*, 2014; Laker *et al*, 2017; Murakawa *et al*, 2019), we next explored if the ULK1 complex is also required for pexophagy. ULK1 and ATG13 were first silenced by siRNA, and then cells were treated with DFP. We observed that the silencing of these two proteins significantly prevented DFP-induced loss of HSP60 and catalase, as monitored by immunoblot (Fig 1D). The block in pexophagy was further confirmed in siRNA-treated *pexo*-QC cells, by both immunoblot and FACS analysis (Fig EV1H and I). Interestingly, the levels of LC3-I were strongly upregulated in both the ATG13 and the ULK1 siRNA-treated cells, which is likely reflective of impaired autophagy in general (Fig 1D). Taken together, loss of ATG13 or ULK1 impairs the flux of mitochondrial and peroxisomal proteins, demonstrating that both DFP-induced mitophagy and pexophagy require the conventional autophagy machinery.

NIX is required for mitophagy and pexophagy

As mitophagy and pexophagy were stimulated upon iron chelation or hypoxia, and given that both conditions led to the stabilisation of the oxygen-sensitive transcription factor HIF1α, we next investigated the role of HIF1α in pexophagy. We previously found a significant block in DFP-induced mitophagy in U2OS HIF1α KO cells (Zhao *et al*, 2020); thus, we expressed the *pexo*-QC reporter in these

 © 2022 The Authors

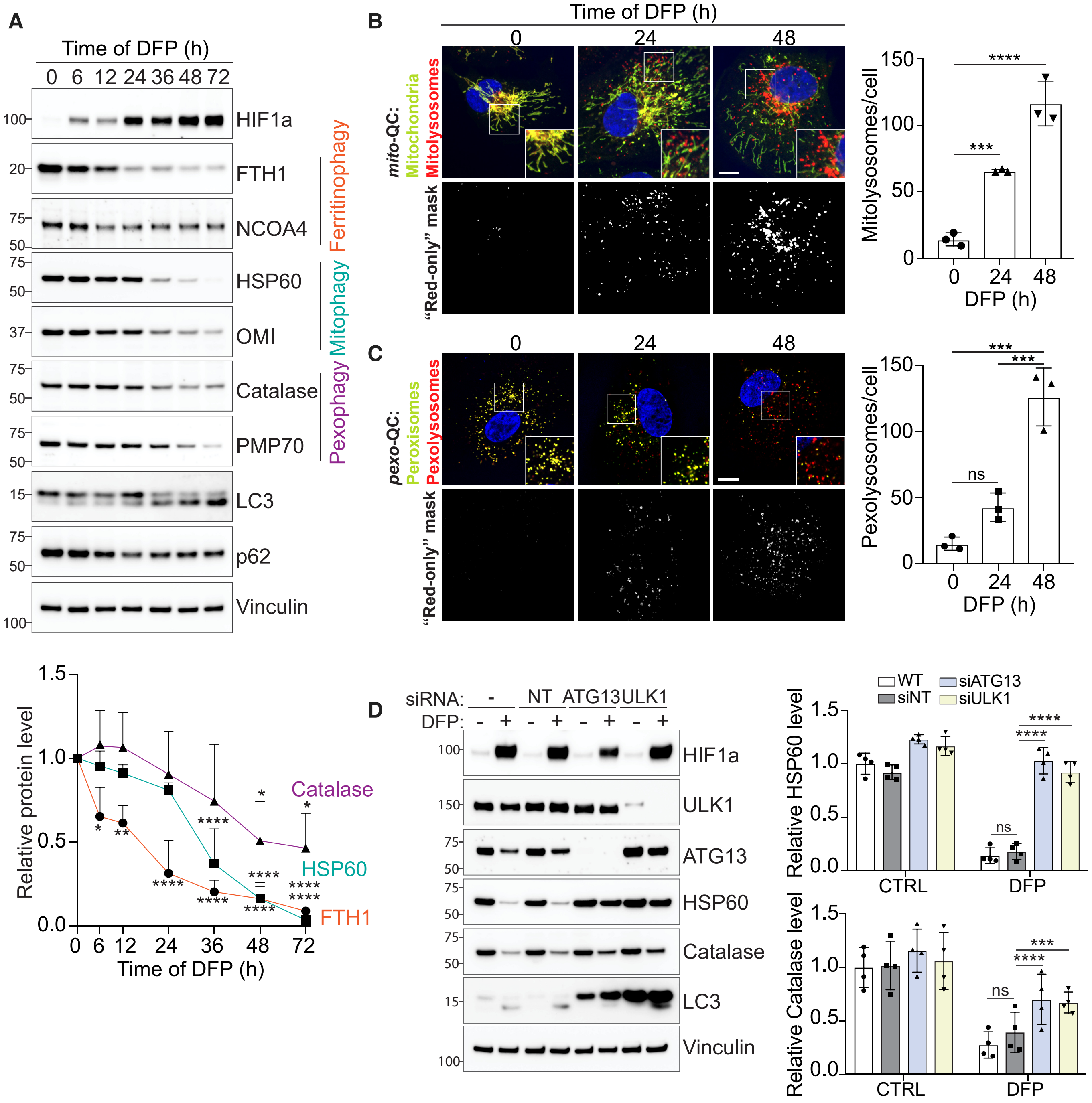

Figure 1. Iron chelation sequentially induces selective autophagy pathways.

A Representative immunoblots and quantifications (below) of the indicated proteins in lysates of ARPE-19 cells treated with 1 mM DFP for the indicated time (h, hours).

B, C Representative confocal images of ARPE-19 cells stably expressing the *mito*-QC reporter (B), or the *pexo*-QC reporter (C) treated with 1 mM DFP for the indicated time (h). Enlarged images of the area outlined in white are shown in the lower corners. The "red-only" mask is generated during images analysis and highlights red-only puncta (autolysosomes) based on the mCherry/GFP ratio and thresholding criteria. Nuclei were stained in blue (Hoechst). Scale bar: 10 μm. At right, quantification of total red-only punctate per cell (mitolysosomes or pexolysosomes).

D ARPE-19 cells were transfected with 50 pmol of non-targeting siRNA (siNT) or 50 pmol of siRNA targeting ATG13 or ULK1. 48 h post knockdown, cells were treated with 1 mM DFP for an additional 48 h. Cell lysates were subject to immunoblotting with the indicated antibodies. Quantification is shown on the right.

Data information: All data are mean ± s.d.; Statistical significance is displayed as *$P \leq 0.05$; **$P \leq 0.01$; ***$P \leq 0.001$; ****$P \leq 0.0001$; ns, not significant. (A) $n = 4$ biological replicates, one-way ANOVA, Dunnett's multiple comparisons test. (B, C) $n = 3$ biological replicates with > 40 cells per replicate, one-way ANOVA, Tukey's multiple comparisons test. (D) $n = 4$ biological replicates, two-way ANOVA, Sidak's multiple comparisons test.
Source data are available online for this figure.

cells. As observed for mitophagy, pexophagy was strongly impaired in HIF1α-depleted cells (Fig 2A and B). Thus, HIF1α is essential for iron chelation-induced mitophagy and pexophagy.

Two HIF1α-regulated OMM-anchored proteins, NIX and closely related BNIP3, have previously been shown to trigger mitophagy via a direct interaction with the autophagy machinery, thus bypassing the need for ubiquitin-binding receptors (Zhang & Ney, 2009; Novak *et al*, 2010; Hanna *et al*, 2012). Of relevance here is that we have previously shown, in SH-SY5Y cells, that NIX and BNIP3 are essential for DFP-induced mitophagy (Zhao *et al*, 2020). We thus hypothesised that loss of NIX in ARPE-19 cells would block DFP-induced mitophagy and allow us to test the effects of this on pexophagy pathway. We therefore used the CRISPR system to generate NIX knockout (KO) ARPE-19 cells.

Using two different CRISPR-generated KO clones (Cl31 and Cl36), we confirmed loss of NIX by Western blot (Fig 2C) and by genomic sequencing (see Materials and Methods). Loss of NIX did not appear to alter upstream HIF1 signalling, as evidenced by the stabilisation of HIF1α. However, the DFP-induced loss of multiple mitochondrial proteins was significantly impaired in both KO clones, indicating that mitophagy was blocked (Fig 2C). Interestingly, we observed that peroxisomal proteins, such as catalase and PMP70, were also not cleared after DFP treatment, suggesting that DFP-induced pexophagy is also impaired in NIX-depleted cells (Fig 2C). Conversely, the loss of FTH1, whose degradation takes place before mitophagy and pexophagy (Fig 1A), was not affected by NIX levels (Fig 2C). This indicates that NIX is not required for DFP-induced ferritinophagy and that loss of NIX does not cause a global block in general autophagy.

We also examined DFP-induced mitophagy and pexophagy in our reporter cell lines. As previously found by immunoblot and microscopy, FACS-based *mito*-QC analysis showed that DFP treatment induced an increase of the number of cells undergoing mitophagy in a time-dependent manner (Fig 2D). By contrast, in NIX KO cells, DFP-induced mitophagy was abolished at 24 h and strongly impaired at 48 h (Fig 2D). Confocal microscopy analysis confirmed that NIX-depleted cells form much fewer mitolysosomes upon DFP treatment compared with WT cells (Fig 2D). A similar scenario was observed with pexophagy: 48 h of DFP treatment induced a significant increase in the number of cells undergoing pexophagy, but only in NIX-expressing cells (Fig 2E). Consistently, confocal microscopy analysis showed that NIX-depleted cells had significantly fewer pexolysosomes. Therefore, by both endogenous Western blot and exogenous autophagy reporters, loss of NIX impairs the turnover of both mitochondria and peroxisomes.

To confirm that the results observed in NIX-depleted cells are not due to an off-target effect of the CRISPR system, we performed rescue experiments in NIX-depleted cells (Fig 2F). As expected, NIX-depleted cells expressing an empty vector are defective in the clearance of mitochondrial (OMI) and peroxisomal (catalase) proteins. By contrast, when NIX was re-expressed in KO cells, DFP-induced mitophagy and pexophagy were restored to WT levels, corroborating that NIX is required for both mitophagy and pexophagy. We found that overexpression of NIX alone in the KO cells, in the absence of DFP, was insufficient to restore mitophagy and pexophagy even though levels were comparable with WT cells treated with DFP. This suggests that although NIX is essential for mitophagy and pexophagy, it is not sufficient for a robust response by itself in these cells. We are currently exploring what these additional HIF1-dependent inputs might be. It is also important to note that the endogenous and exogenous protein levels of NIX differ in response to DFP. This is likely because endogenous NIX has the HIF1-responsive elements within its promoter and is strongly induced upon DFP treatment, to a level that exceeds the rate of mitophagy/pexophagy, hence it accumulates. By contrast, exogenous NIX has no such promoter elements and is expressed constitutively at a rate that does not exceed that of its turnover via mitophagy/pexophagy; hence, its levels decrease with DFP treatment.

We did notice, especially in the more sensitive reporter-based cell assays, that a small degree of pexophagy still occurred in the absence of NIX. Given our previous data showing a degree of redundancy between NIX and BNIP3, a close homologue of NIX, for DFP-induced mitophagy (Zhao *et al*, 2020 and Fig EV2A–B), we reasoned that this residual pexophagy may be due to BNIP3 involvement. To test this, we employed siRNA of both NIX and BNIP3 (Fig EV2). In the *pexo*-QC reporter cells, NIX or BNIP3 were first silenced by siRNA, and then cells were treated with DFP to induce pexophagy (Fig EV2A). We observed that the silencing of not only NIX, but also BNIP3, impaired DFP-induced pexophagy (Fig EV2C). In addition, the silencing of these two proteins together completely abolished DFP-induced pexophagy suggesting that NIX and BNIP3 share similar functions in pexophagy pathway (Fig EV2C). Given the data showing a strong pexophagy block with the more complete loss of NIX in the CRISPR clones (as well as its rescue), we assume that in these cells, NIX plays the prime role in mediating pexophagy. However, these data suggest that BNIP3 can also regulate

Figure 2. Depletion of NIX restricts both mitophagy and pexophagy.

- A Representative immunoblots of the indicated proteins in lysates of WT or HIF1α KO U2OS cells, treated with 1 mM DFP for 48 h.
- B Representative confocal images of WT or HIF1α KO U2OS cells stably expressing the *pexo*-QC reporter and treated with 1 mM DFP for 48 h (left panel) and flow cytometry analysis of the mCherry/GFP ratio (right panel).
- C Representative immunoblots (left panel) and quantification (right panel) of the indicated proteins in lysates of WT or two NIX KO ARPE-19 clones (Cl), treated with 1 mM DFP for the indicated time (h).
- D, E Representative confocal images of WT or NIX KO ARPE-19 cells stably expressing the *mito*-QC reporter (D) or the *pexo*-QC reporter (E) treated with 1 mM DFP for 48 h (left panel) and flow cytometry analysis of the mCherry/GFP ratio (right panel).
- F Representative immunoblots (left panel) and quantification (right panel) of the indicated proteins. NIX KO ARPE-19 cells (Cl 31) stably expressing a pBabe empty vector (∅) or a pBabe NIX vector, were treated with 1 mM DFP for 48 h prior lysis. Two biological replicates are shown in the immunoblot.

Data information: Enlarged images of the area outlined in white are shown in the lower corners. Scale bar: 10 μm. Overall data are mean ± s.d.; Statistical significance is displayed as *$P \leq 0.05$; **$P \leq 0.01$; ***$P \leq 0.001$; ****$P \leq 0.0001$; ns, not significant. (B) $n = 3$ biological replicates; (C) $n = 3$ biological replicates, statistical comparisons were made within the same time point; (D, E) $n = 4$ biological replicates; (F) $n = 4$ biological replicates; two-way ANOVA, Tukey's or Sidak's multiple comparisons test. Source data are available online for this figure.

▶

 © 2022 The Authors

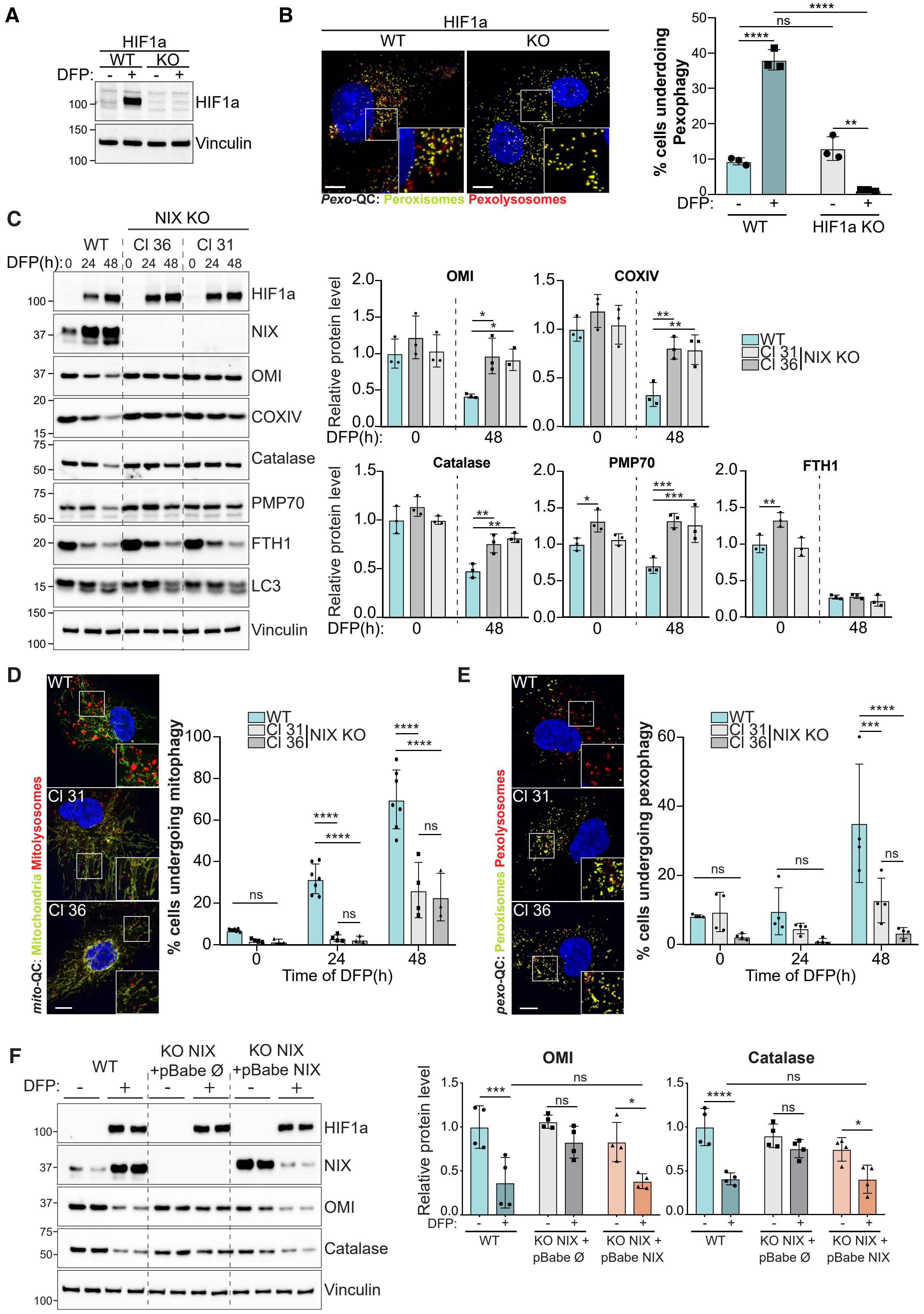
A
HIF1a
WT
KO
DFP:
HIF1a
Vinculin
B
HIF1a
WT
KO
Pexo-QC: Peroxisomes Pexolysosomes
% cells underdoing Pexophagy
DFP:
WT
HIF1a KO
C
NIX KO
WT
Cl 36
Cl 31
DFP(h):
HIF1a
NIX
OMI
COXIV
Catalase
PMP70
FTH1
LC3
Vinculin
OMI
COXIV
Catalase
PMP70
FTH1
Relative protein level
WT
Cl 31
Cl 36
NIX KO
D
WT
Cl 31
Cl 36
mito-QC: Mitochondria Mitolysosomes
% cells undergoing mitophagy
Time of DFP(h)
E
pexo-QC: Peroxisomes Pexolysosomes
% cells undergoing pexophagy
Time of DFP(h)
F
WT
KO NIX +pBabe Ø
KO NIX +pBabe NIX
DFP:
HIF1a
NIX
OMI
Catalase
Vinculin
OMI
Catalase
KO NIX + pBabe Ø
KO NIX + pBabe NIX

Figure 2.

pexophagy, and this may be more relevant in other cell types or signalling conditions. More work is needed to confirm this.

Altogether, these results reveal that the depletion of NIX significantly impairs DFP-induced pexophagy, in addition to restricting DFP-induced mitophagy. This suggests an interconnection between these two selective autophagy pathways.

NIX localises to peroxisomes upon iron chelation

How does NIX, a transmembrane mitochondrial protein, play a role in pexophagy? It is possible that effect is indirect, given that mitochondria and peroxisomes can be in close proximity: turnover of a mitochondrion may also lead to turnover of a peroxisome if it is in direct contact. Interestingly, it has been recently reported that USP30, an integral protein of the outer mitochondrial membrane, can also localise to peroxisomes to negatively regulate pexophagy (Marcassa *et al*, 2018). We therefore hypothesised that, as for USP30, NIX function in pexophagy may rely on its translocation from mitochondria to peroxisomes. To help rule this in or out, we first determined whether NIX could localise to peroxisomes. To easily visualise the localisation of NIX upon DFP treatment, we generated cells that stably express GFP-NIX (as discussed below, we also analysed endogenous NIX extensively). As expected, under basal conditions, GFP-NIX formed a tubular network into the cytoplasm of cells, which did not obviously colocalize with the peroxisomal proteins catalase and PMP70 (Fig 3A and B). By contrast, when cells were treated with DFP for a time sufficient to induce pexophagy (48 h), a significant proportion of GFP-NIX was now found colocalizing with peroxisomes (Fig 3A and B). This result was also confirmed with endogenous NIX (Fig EV3A and B). In untreated cells, endogenous NIX is expressed at low levels, and minimal colocalisation was observed in peroxisomes. However, upon DFP treatment, NIX expression was increased, and this signal was enriched on peroxisomes in a time-dependent manner. In addition, hypoxia also induced translocation of GFP-NIX or endogenous NIX to peroxisomes (Fig EV3C), and GFP-BNIP3 was also found enriched on peroxisomes upon DFP (Fig EV3D). Of note, GFP-NIX, endogenous NIX and GFP-BNIP3 marked larger peroxisomal structures, which could indicate clustering of peroxisomes (Figs 3 and EV3A–D). It is interesting to note that damaged mitochondria can aggregate and form perinuclear clusters, which has been proposed to aid in mitophagy (Vives-Bauza *et al*, 2010; Narendra *et al*, 2010a). Similarly, peroxisomal clustering/enlargement could be an important stage of DFP-induced pexophagy. Indeed, other studies have already reported that peroxisomes are larger following pexophagy induction and accumulate the autophagic receptors p62 and NBR1 (Deosaran *et al*, 2013; Jo *et al*, 2020). As enlarged peroxisomes are enriched for NIX, this phenotype could favour their lysosomal degradation. However, we cannot yet exclude that it could be a protective mechanism to avoid enforced depletion of peroxisomes by pexophagy. Further work is needed to determine the significance of this.

NIX is a multidomain protein, including a LIR motif on the N-terminal part exposed to the cytoplasm (Novak *et al*, 2010), which can be phosphorylated (Rogov *et al*, 2017); an atypical BH3-like domain; and a transmembrane domain (TM) on the C-terminal part. In addition to mitochondrial localisation, the transmembrane domain is important for NIX dimerisation and mitophagy activation (Marinković *et al*, 2021). We next generated a truncation mutant of NIX lacking the TM (GFP-NIX ΔTM) or we expressed only the TM of NIX fused to GFP (GFP-NIX TM only), as summarised in the left panel of Fig EV3E. As previously described, GFP-NIX ΔTM was mainly diffuse in the cytoplasm and in the nucleus, while the TM only localised at the mitochondria (Figs 3C and EV3E). Following DFP treatment, GFP-NIX ΔTM did not colocalize with peroxisomes as observed with WT NIX and NIX TM only (Fig 3C). These data indicate that NIX localisation to mitochondria and peroxisomes relies on the same targeting signal, present within the transmembrane domain, and suggests that NIX becomes an integral peroxisomal protein.

To determine whether NIX colocalisation with peroxisomes occurs at sites of mitochondrial contact, we stained GFP-NIX cells for both peroxisomal and mitochondrial markers (Fig 3D). Here, we were able to observe NIX colocalising with peroxisomal structures (catalase) that were clearly distinct and removed from mitochondrial structures (ATPB). Likewise, NIX-positive mitochondria were distinct from NIX-positive peroxisomes. This strongly implies that NIX does not colocalise with peroxisomes solely at mitochondrial contact sites (Fig 3D).

NIX is translocated to peroxisomes independently of mitochondria

As we found no evidence that NIX localised with peroxisomes at mitochondrial contact sites, we next explored the possibility that after insertion into mitochondria, NIX shuttles to peroxisomes via the MDV (mitochondrial-derived vesicles) pathway. Indeed, MDVs have been shown to transport cargo between mitochondria and peroxisomes, such as the ubiquitin E3 ligase MAPL/MUL1 (Neuspiel *et al*, 2008; Braschi *et al*, 2010). To test this hypothesis, mitochondria were depleted using an HA-Parkin E3 ligase overexpression

Figure 3. NIX localises to peroxisomes via its transmembrane domain.

A, B Representative confocal images of untreated (CTRL) and DFP-treated ARPE-19 cells (1 mM, 48 h), stably expressing GFP-WT NIX (green) and stained with anti-catalase antibody (A, red) or anti-PMP70 antibody (B, red). At right, quantification of Pearson correlation coefficients between NIX and the peroxisomal markers catalase and PMP70, with or without DFP treatment for 48 h. Peroxisomes enriched for GFP-NIX or having minimal signal for GFP-NIX are depicted with white arrowheads or white arrows, respectively.

C Representative confocal images of ARPE-19 cells stably expressing GFP-WT NIX, GFP-NIXΔTM or GFP-NIX TM only (green), treated with DFP (1 mM) for 48 h and immunostained with anti-catalase antibody (red). At right, quantification of Pearson correlation coefficients between the green and the red signal. Overall data are mean ± s.d.; Statistical significance is displayed as $**P \leq 0.01$; $***P \leq 0.001$; $****P \leq 0.0001$; ns, not significant. $n = 3$ biological replicates with > 15 cells per replicate, (A, B) unpaired *t*-test, two tailed; (C) one-way ANOVA, Tukey's multiple comparisons test.

D Representative confocal images of untreated (CTRL) and DFP-treated ARPE-19 cells (1 mM, 48 h), stably expressing GFP- WT NIX (green) and immunostained with a mitochondrial marker (ATPB, cyan) and peroxisomal marker (catalase, red).

Data information: Enlarged images of the area outlined in white are shown on the right. Nuclei were stained in blue (Hoechst) and scale bars: 10 μm.

 © 2022 The Authors

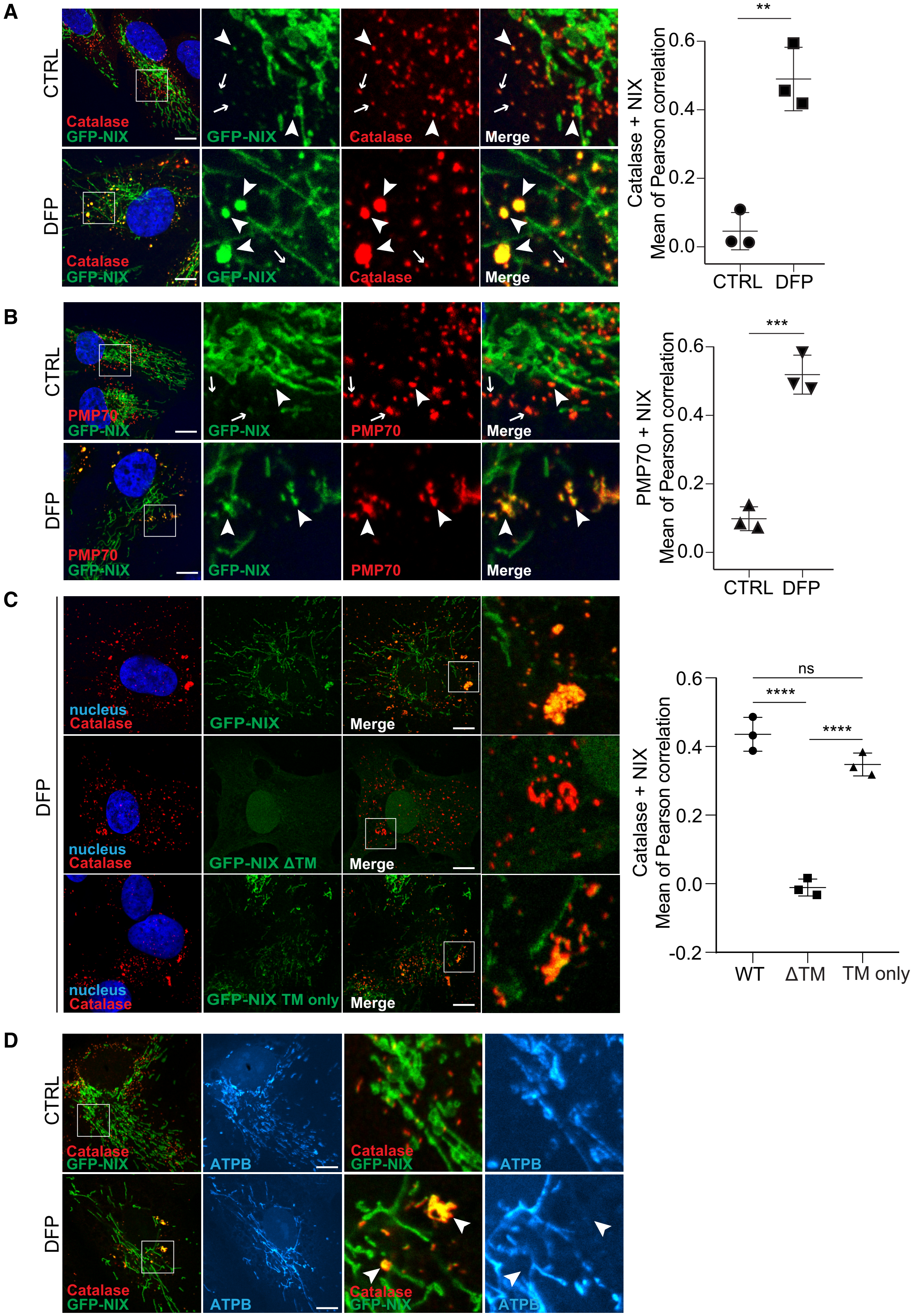
A
CTRL
DFP
Catalase
GFP-NIX
Merge
Catalase + NIX
Mean of Pearson correlation
**
0.6
0.4
0.2
0.0
CTRL
DFP
B
PMP70
PMP70 + NIX
***
C
nucleus
GFP-NIX ΔTM
GFP-NIX TM only
ns
****
-0.2
WT
ΔTM
TM only
D
ATPB

Figure 3.

approach, as previously described (Correia-Melo *et al*, 2017). In this system, prolonged CCCP treatment results in total clearance of mitochondria by enforced mitophagy. Of relevance here, such an approach was used to show that USP30 can localise to peroxisomes independently of mitochondria (Marcassa *et al*, 2018). Therefore, we treated Parkin overexpressing cells with CCCP to remove mitochondria. Following this, we removed CCCP and incubated with DFP to induce NIX expression and determined its localisation (as summarised in Fig 4A). When ARPE-19 cells, overexpressing HA-Parkin, were depolarised with CCCP for 24 h, this led to a complete loss of mitochondrial markers by immunoblot and by confocal microscopy, without affecting peroxisome abundancy (Fig 4B and C). This treatment also led to a drastic decrease of NIX, suggesting that the majority of NIX under these conditions is localised to mitochondria, in support of our previous data from control treated cells in Fig 3. Following DFP treatment and induction of the HIF1 transcriptional program, NIX levels were increased, in both the control and the HA-Parkin expressing cells. However, in the HA-Parkin

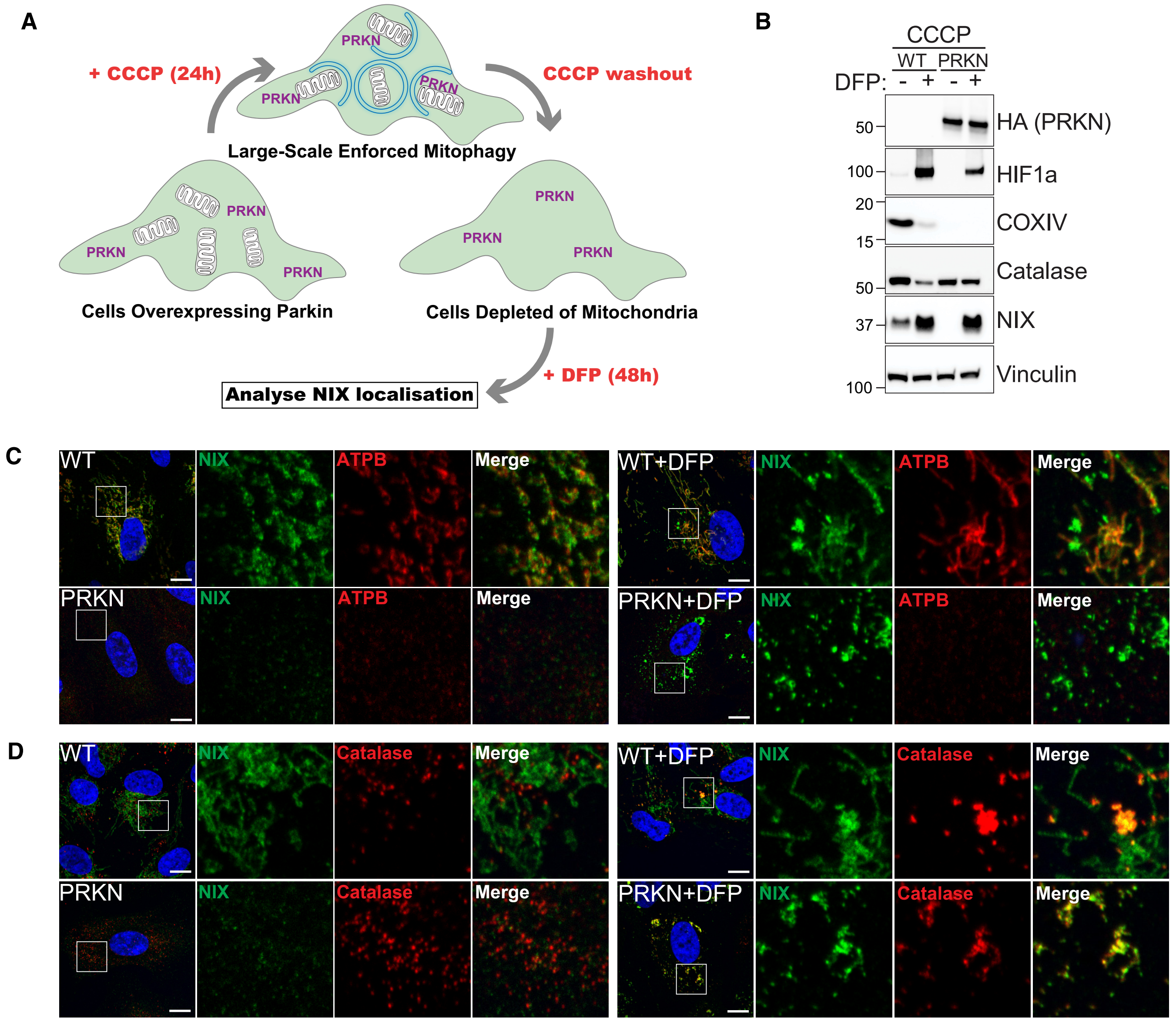

Figure 4. NIX is targeted to peroxisomes in the absence of mitochondria.

A Schematic illustration of mitochondria depletion in cells overexpressing Parkin (PRKN). Cells are pre-treated for 24 h with CCCP (20 μM), and after 1 day of recovery (CCCP washout), treated for 48 h with 1 mM DFP.

B Representative immunoblots of the indicated proteins in lysates of WT or HA-Parkin (PRKN)-ARPE-19 cells treated as in (A).

C, D Representative confocal images of HA-Parkin (PRKN) ARPE-19 cells treated as in (A) and stained with anti-NIX antibody (green) and anti-ATPB antibody (red, C) or anti-Catalase antibody (red, D). Enlarged images of the area outlined in white are shown on the right. Nuclei were stained in blue (Hoechst) and scale bars: 10 μm.

Source data are available online for this figure.

© 2022 The Authors

overexpressing cells that now lacked mitochondria, NIX was still localised to punctate structures that colocalise with the peroxisomal marker catalase (Fig 4C and D). Therefore, newly synthesised NIX was still able to reach peroxisomes in the absence of mitochondria, suggesting that a pool of NIX is localised to peroxisomes, independently of mitochondria and MDVs, and contributes to DFP-induced pexophagy.

Although Parkin in this instance was used to artificially remove mitochondria, it does highlight that ubiquitin can also play a major role in autophagic cargo selection. Parkin-dependent mitophagy can be stimulated in cells following mitochondrial depolarisation with reagents such as CCCP or Oligomycin A/Antimycin A (O/A) (Kondapalli *et al*, 2012; Allen *et al*, 2013). In addition, other E3 ligases can ubiquitylate mitochondria to stimulate mitophagy, as is the case with Ivermectin-induced mitophagy (Zachari *et al*, 2019). We therefore were interested to see whether we could detect ubiquitylation of peroxisomes following DFP treatment (Fig EV4A and B). We analysed the level of mono- and polyubiquitin in cells treated with DFP, or with known ubiquitin-dependent mitophagy inducers (CCCP, O/A, or Ivermectin) (Allen *et al*, 2013; Zachari *et al*, 2019). No global increase in the level of protein ubiquitylation was found under any treatment (Fig EV4A). However, the cellular PINK1/Parkin activators, CCCP and O/A, both induced a clear increase in PINK1-dependent phospho-ubiquitin. Given that CCCP, O/A and ivermectin are known to result in ubiquitylation of mitochondria, as visualised by immunofluorescence microscopy, we also stained cells with the FK2 antibody to detect ubiquitin aggregates (Fig EV4B). While untreated cells showed a diffuse ubiquitin signal, those treated with CCCP, O/A or Ivermectin displayed the expected ubiquitin puncta, which have been previously shown to localise to mitochondria (Zachari *et al*, 2019). By contrast, no ubiquitin puncta were observed in DFP-treated cells. This suggests that DFP does not stimulate mitophagy and pexophagy through a ubiquitin-like mechanism in the manner of O/A or ivermectin-induced mitophagy. In support of this, O/A treatment failed to induce pexophagy, as visualised in the *pexo*-QC reporter line (Fig EV4C). We do note that p62/SQSTM1, the archetypal ubiquitin-binding cargo receptor, is lost following DFP treatment (Fig 1A) suggesting it is being autophagocytosed. However, we observed no colocalisation of p62/SQSTM1 with peroxisomes following DFP treatment, which contrasts with amino acid starvation conditions that have been shown to stimulate pexophagy through the ubiquitin-cargo selection pathway (Fig EV4D; Deosaran *et al*, 2013). Ubiquitin is likely involved in DFP-induced pexophagy at multiple stages, but further work is needed to determine the exact involvement and its precise roles.

NIX LC3 interacting region and dimerisation regulate pexophagy

To confirm whether mitochondria and peroxisomes can be independently autophagocytosed, we used confocal microscopy to examine mitophagosomes and pexophagosomes formed upon iron chelation (Fig 5A). Both mitochondria and peroxisomes were found in LC3-positive structures, but rarely were all three colocalised together (see white arrowhead in Fig 5A). Therefore, these two organelles can be targeted independently by the autophagy machinery. However, given the small degree of mitochondrial-peroxisomal colocalisation, we cannot rule out that under some circumstances, both a mitochondrion and peroxisome can be engulfed together by the same autophagosome. We do note that these structures could also represent later compartments, such as autolysosomes that have fused with both mitophagosomes and pexophagosomes.

As we saw colocalisation between peroxisomes and LC3, we reasoned that the ability of NIX to interact with LC3 (and other ATG8s) may play a critical role. NIX has previously been shown to bind to autophagic ATG8-proteins through a conserved tetrapeptide LIR motif, and this is required for efficient NIX-dependent mitophagy (Novak *et al*, 2010). In addition, the phosphorylation of serine residues juxtaposed to the core NIX LIR appear to contribute to the interaction with LC3 proteins (Rogov *et al*, 2017). To test whether the NIX:LC3 interaction, via the LIR, is required for pexophagy, we generated two LIR mutants: (i) the conserved tryptophan in position 36 (W36) and the conserved leucine in position 39 (L39), were mutated to alanine (W36A/L39A) to abolish NIX binding to LC3 (Novak *et al*, 2010). (ii) the serine residues in position 34 and 35 were mutated to glutamic acid residues to mimic phosphorylation and increase interaction with LC3 (S34E/S35E) (Rogov *et al*, 2017). We expressed these NIX LIR mutants in NIX KO ARPE-19 cells expressing the *mito*-QC or *pexo*-QC reporter (Fig 5C and D). With respect to mitophagy, our results followed previous work: NIX-depleted cells re-expressing WT NIX showed a significant increase in the number of cells undergoing mitophagy, but in cells expressing the W36A/L39A LIR mutant, mitophagy was significantly impaired compared with WT-expressing cells. The same situation was observed for pexophagy. We also noted that the W36A/L39A LIR mutant was localised to mitochondria under control conditions (Fig EV5C) and was enriched on peroxisomes upon DFP (Fig EV5D), excluding the possibility that pexophagy defects are

Figure 5. NIX LIR domain is required for mitophagy and pexophagy.

A Representative confocal images of ARPE-19 cells stably expressing mCherry-SKL (peroxisomes, red), treated with 1 mM DFP for 48 h and 50 nM Bafilomycin A (BafA) for the last 6 h prior imaging. Mitochondria were stained with anti-COXIV antibody (green) and autophagosomes with anti-LC3 antibody (cyan). Green-only, red-only, and green and red positive structures are depicted with yellow, red and white arrowheads, respectively. Scale bars for (i) and (ii) is 2 µm. Enlarged images of the area outlined in white are shown on the right.

B Representative immunoblots of the indicated proteins in lysates of NIX KO ARPE-19 cells (Cl 31) stably expressing a pBabe Flag vector (∅), a pBabe Flag-NIX vector (NIX), a pBabe Flag-NIX W36A\L39A vector (W36A\L39A) or a pBabe Flag-NIX S34E\S35E vector (S34E\S35E), and treated with 1 mM DFP for 48 h prior lysis.

C, D Representative confocal images of cells as in (B), stably expressing the *mito*-QC reporter (C) or the *pexo*-QC reporter (D) and treated with 1 mM DFP for 24 h (C) or 48 h (D) (left panel) and flow cytometry analysis of the mCherry/GFP ratio (right panel). Enlarged images of the area outlined in white are shown in the lower corners. Nuclei were stained in blue (Hoechst) and scale bars: 10 µm.

Data information: Overall data are mean ± s.d.; Statistical significance is displayed as $^{*}P \leq 0.05$; $^{***}P \leq 0.001$; $^{****}P \leq 0.0001$; ns, not significant. (C) $n = 3$ biological replicates; (D) $n = 4$ biological replicates; one-way ANOVA, Tukey's multiple comparisons test.
Source data are available online for this figure.

© 2022 The Authors

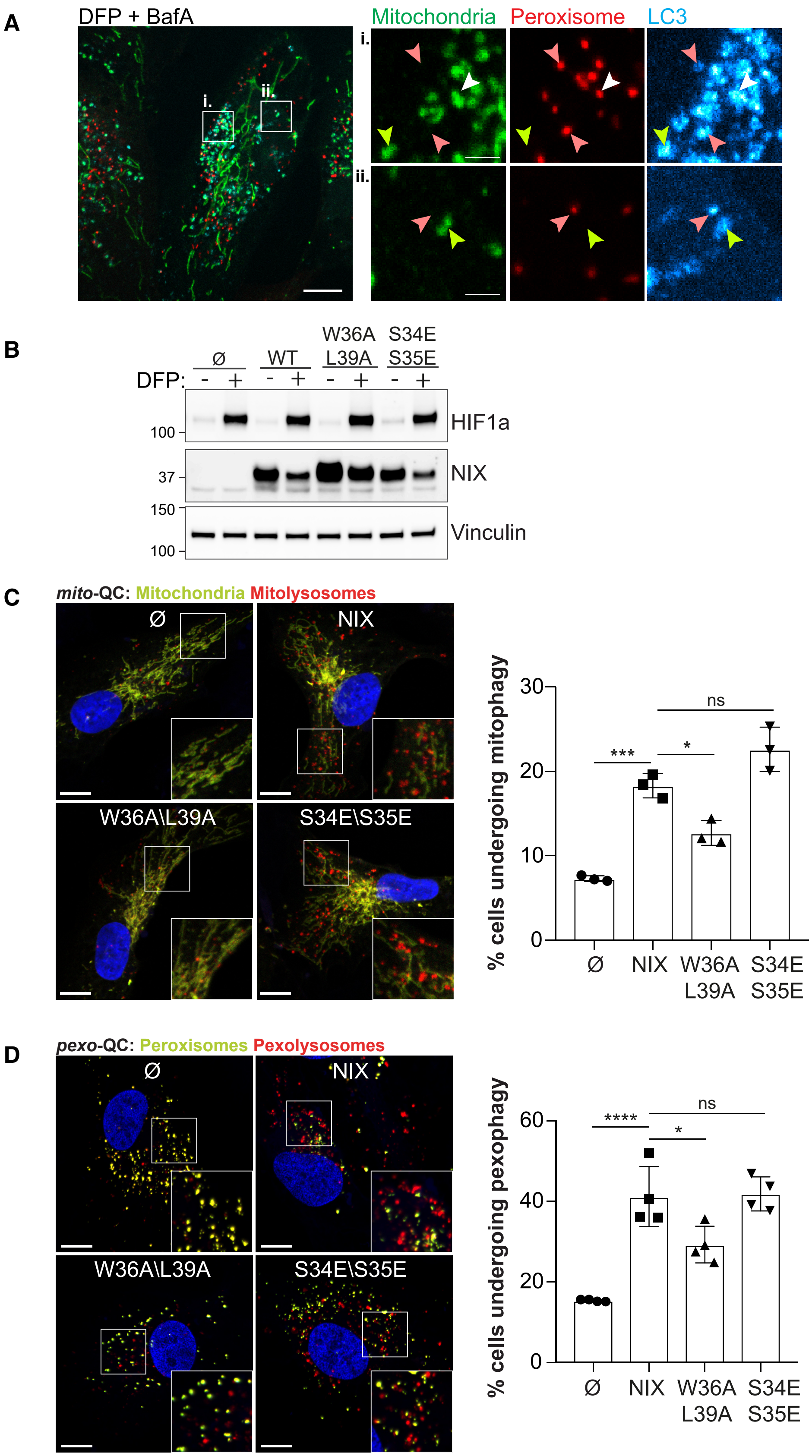

Figure 5.

 © 2022 The Authors

caused by loss of peroxisomal targeting. The phosphomimetic LIR mutant (S34E/S35E) cells could also induce mitophagy and pexophagy to a similar WT level (Fig 5C). Given that the nature of this LIR phosphorylation is currently unknown, as is the kinase responsible (Rogov *et al*, 2017), it is possible that a lack of stimulation over WT levels is due to already high phosphorylation levels. These results show that the LIR domain is required for both efficient mitophagy and pexophagy and indicate that LC3 (and possibly other ATG8s) are important for these processes (Fig 5B–D).

Dimerisation of NIX is also essential for its ability to stimulate mitophagy (Marinković *et al*, 2021). This also appears to be the case for pexophagy, as a mutation in the TM domain (G204A) that abolishes the ability of NIX to dimerise (Marinković *et al*, 2021), failed to rescue both mitophagy and pexophagy in NIX KO cells (Fig EV5B). In addition, we also found that G204A mutation impaired NIX expression and localisation. Indeed, G204A levels were lower than WT NIX (Fig EV5A), and the G204A dimer mutant was observed not only at the mitochondria but also diffuse in the cytoplasm and nucleus (Fig EV5C). Upon DFP, G204A dimer mutant was not dramatically enriched on peroxisomes (Fig EV5D), suggesting that the transmembrane region of NIX is required for NIX dimerisation, efficient localisation at mitochondria and peroxisomes and likely NIX stability. All together, these experiments indicate that NIX is a selective autophagy receptor that dimerizes and binds LC3 for mitochondria and peroxisome turnover.

NIX regulates peroxisomal levels in physiological contexts

We have shown that iron chelation, with DFP, robustly induces NIX-dependent pexophagy in multiple cell lines. However, the physiological significance of this is less clear. To gain insight into this, we next looked in mouse tissues that lacked NIX expression (whole body NIX KO mice). We decided to examine pexophagy in the mouse retina, based on the following rationale. First, we have previously shown that significant mitophagy occurs within defined areas of the mouse retina (McWilliams *et al*, 2018, 2019). Thus, we assumed given our newly discovered link between mitophagy and pexophagy, that this area could yield interesting results. Second, we have previously shown that DFP induces NIX-dependent mitophagy via hypoxia-related HIF1α-dependent transcription (Zhao *et al*, 2020) and the eye, with its limited vasculature, is a particularly hypoxic organ (Scott & Fruttiger, 2010). If pexophagy is blocked in the retina of NIX KO mice, then we would predict that they would contain increased peroxisomal content. To look at this, we stained retinas for the peroxisomal markers PMP70 and catalase (Fig 6). This revealed a significant increase in both markers in the inner nuclear layer (INL), which is enriched in bipolar, horizonal and amacrine cells. We also saw a significant increase in catalase staining in the outer nuclear layer (ONL), and a trending increase for PMP70 (Fig 6A and B). This is of interest as this region, where the soma of the photoreceptors are localised, also has a relatively high rate of mitophagy (McWilliams *et al*, 2019). This suggests a coordination of mitophagy and pexophagy *in vivo*, though further work, for example with tissue-specific knockout of core autophagy genes, would help to confirm this as well as rule out peroxisomal biogenesis defects. As a control, we also stained retinal sections for lysosomes using LAMP1 (Fig 6A and B). In contrast to peroxisomes, we detected no significant change in lysosomal content in both the INL and the ONL, implying that loss of NIX does not result in an increase of organelles in general.

NIX-dependent mitophagy has been shown to be essential during differentiation, in particular to regulate metabolic switches during retinal ganglion cell differentiation and macrophage activation (Esteban-Martinez *et al*, 2017). Thus, to further examine the role of mitophagy and pexophagy in more physiological contexts, we studied two more examples where NIX-dependent mitophagy has been seen to occur developmentally. A striking physiological instance of mitophagy occurs during erythrocyte maturation. Previous studies have shown that during erythropoiesis, mitochondria are eliminated by NIX-dependent mitophagy (Sandoval *et al*, 2008; Mortensen *et al*, 2010; Honda *et al*, 2014; Geng *et al*, 2021). To determine whether peroxisomes were also cleared under the same conditions, we differentiated $CD34^+$ derived primary human erythroid cells into reticulocytes (Fig EV6A–C). To achieve this, $CD34^+$ cells were isolated from human peripheral blood from three individual healthy donors and the stem cells proliferated and differentiated over of total of 20 days in culture (Griffiths *et al*, 2012; Kupzig *et al*, 2017). Cells at different stages of maturation were collected and differentiation status was confirmed by staining and flow cytometry of Band3, an anion exchanger and marker of mature erythrocytes, and α-4-integrin, a cell surface adhesion molecule that is lost during differentiation (Fig EV6A) (details previously described; Hu *et al*, 2013). This was accompanied by stained cytospin preparations to monitor erythroblast morphology and enucleation (Fig EV6B). During differentiation, as expected, mitochondrial content was progressively reduced, as observed by the decrease of OMI and COXIV proteins (Fig EV6C). We also observed that the peroxisomal marker PMP70 was progressively lost upon differentiation. By contrast, the expression of catalase was not appreciably affected after 20 day of culture (Fig EV6C). However, catalase is a highly active enzyme in erythrocytes, being required for ROS protection and is found free in the cytosol or associated with the plasma membrane (Aviram & Shaklai, 1981; Rocha *et al*, 2015). Thus, uniquely, catalase level is not a marker for peroxisome content in erythroid cells and hence a good control for peroxisome specificity in this experiment. Therefore, the PMP70 expression result infers that peroxisomes are cleared, in addition to mitochondria, suggesting autophagic coregulation of these two organelles during erythropoiesis. We also note that HIF1α and BNIP3 are expressed during differentiation, though their exact role in mitophagy and pexophagy in this instance requires more work. It appears, given the previously mentioned published work in reticulocytes, that the situation is similar to ARPE-19 cells in that NIX is the preferred mitophagy and pexophagy receptor.

Work from our laboratory and others showed that mitophagy was induced during cardiomyocyte differentiation (Gong *et al*, 2015; McWilliams *et al*, 2016; Lampert *et al*, 2019; Zhao *et al*, 2020). To examine pexophagy under these circumstances, we expressed our *mito*-QC and *pexo*-QC reporters in the H9c2 cardiomyoblast cell line and differentiated these using media containing low serum and retinoic acid. At Day 8 of differentiation, both mature cardiomyocyte markers, myosin heavy chain (MHC) and cardiac troponin T, were strongly expressed (Fig EV6D). As expected, mitochondrial biogenesis was evident in *mito*-QC cells as observed by the enhanced levels of mitochondrial HSP60 and COXIV, and GFP. Interestingly, the same was true for peroxisomes in the *pexo*-QC cells: increased peroxisomal marker PMP70 and GFP (Fig EV6D).

© 2022 The Authors

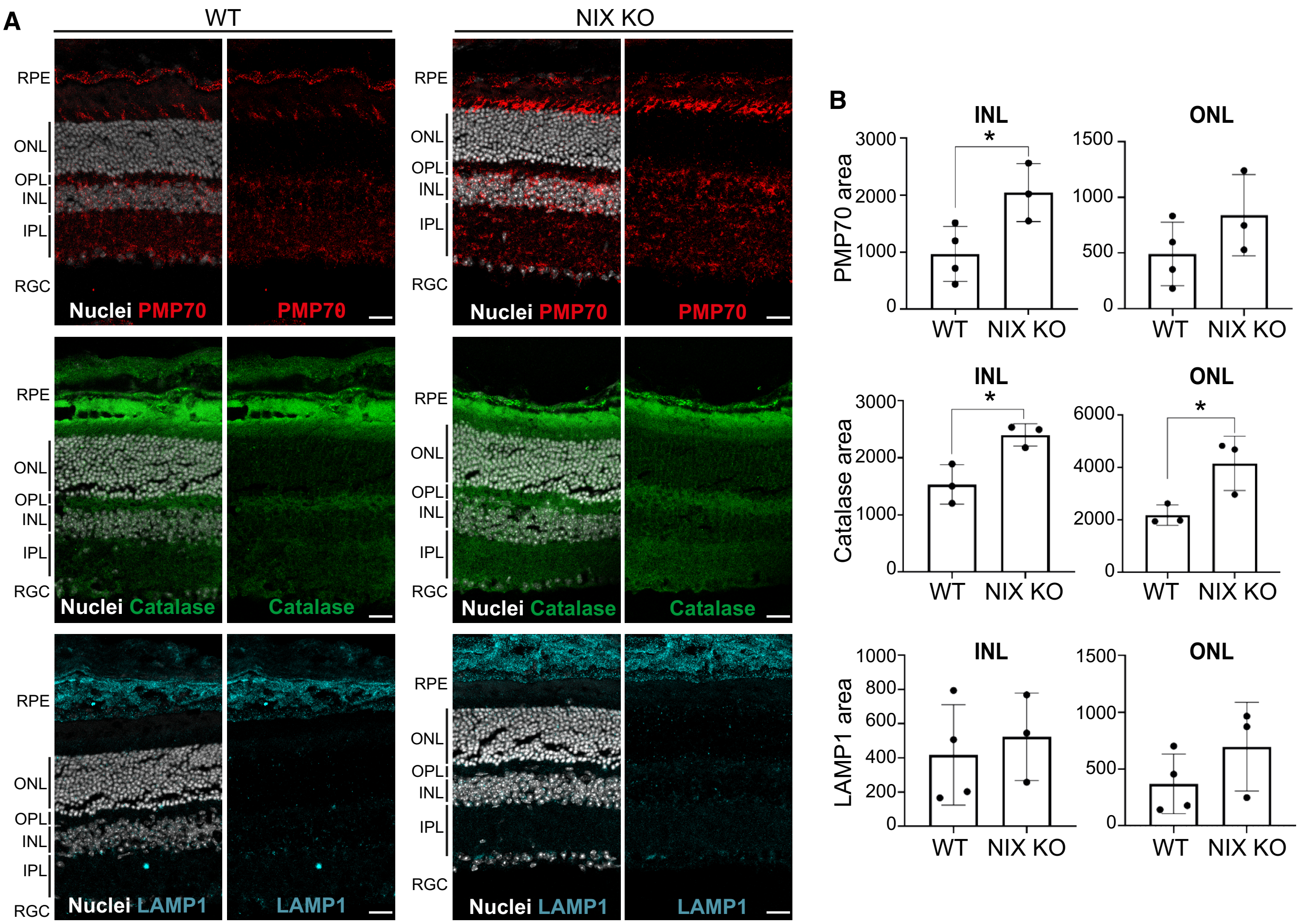

Figure 6. NIX KO mouse retinas are enriched in peroxisomes.

A Representative confocal images of optical section from WT and NIX KO mouse retina immunostained with peroxisomal markers (PMP70 and catalase) or a lysosomal marker LAMP1. Nuclei are coloured in white (DAPI). RPE, retinal pigment epithelium; ONL, outer nuclear layer; OPL, outer plexiform layer; INL; inner nuclear layer; IPL, inner plexiform layer; RGC, retinal ganglion cells. Scale bar: 20 mm.

B Quantification of signal area in the ONL and INL from images as (A). Each data point represents a single mouse (n = 3–4 per group).

Data information: Overall data are mean ± s.d.; Statistical significance is displayed as *$P \leq 0.05$; ns, not significant. Unpaired t-test, two tailed.

Given that our previous work demonstrated a significant increase in mitophagy during differentiation (Zhao *et al*, 2020), we focussed on pexophagy in the *pexo*-QC cells. Here, we observed that pexophagy was also significantly induced, with an increase in pexolysosomes at Day 8 (Fig EV6E). As expected, the *pexo*-QC reporter was localised to peroxisomes, given its complete colocalisation with catalase (Fig EV6F). Additionally, the red-only puncta colocalized with LAMP1, confirming that peroxisomes were indeed delivered to lysosomes for degradation during differentiation (Fig EV6G).

Taken together, these results show that pexophagy occurs under the same physiological conditions that stimulate mitophagy. This further highlights the coregulation of these two autophagic pathways.

Discussion

We have previously identified the iron chelator DFP as a strong PINK1/Parkin-independent inducer of mitophagy (Allen *et al*, 2013). Here, we revealed that multiple selective autophagy pathways can be successively activated following DFP, or hypoxia treatment. We found that DFP treatment led to a rapid loss of ferritin, which was followed by a reduction in mitochondria and then lastly peroxisomes. To gain insight into how these selective autophagy pathways are organised, we demonstrated that the OMM protein NIX, a known mitophagy receptor, is a key coordinator for both mitophagy and pexophagy. Indeed, mitochondrial and peroxisomal turnover were prevented in NIX-depleted cells. In addition, we identified that NIX is also a peroxisomal protein, whose targeting occurs independently of mitochondria and requires the NIX transmembrane domain. Finally, we show that NIX-dependent pexophagy is a physiological process. Not only do we find evidence for pexophagy during cardiomyocyte differentiation and erythropoiesis, both processes where NIX-dependent mitophagy occurs, but also in adult mice retina from NIX KO animals. Thus, our work identifies NIX not only as a key pexophagy receptor but also as a protein that controls the autophagy of the two major eukaryotic metabolic organelles.

 © 2022 The Authors

Our study demonstrates a strong relationship between mitophagy and pexophagy: they can happen simultaneously upon application of the same stimulus, and they rely on the same autophagic receptor, NIX. As has been previously shown, we also found that DFP treatment initially results in ferritinophagy; however, we found that this did not require NIX. Regardless, recent work has demonstrated a connection between iron chelation, ferritinophagy and mitophagy. Hara *et al* (2020) demonstrated that DFP increases mitochondrial ferritin (FTMT) expression on the OMM, which in turn recruits the ferritinophagy receptor NCOA4 to facilitate mitophagy. Therefore, it appears that certain stimuli, such as iron chelation, can lead to the orchestrated autophagy of multiple organelles.

Ubiquitylation is a major signal for specific autophagy, triggering the autophagic engulfment of diverse cargoes such as protein aggregates, intracellular pathogens and mitochondria (Gatica *et al*, 2018; Grumati & Dikic, 2018; Harper *et al*, 2018). The same also appears to be true for peroxisomes during pexophagy. The ubiquitin-binding autophagy receptors p62 and NBR1 were shown to degrade ubiquitin-coated peroxisomes (Kim *et al*, 2008; Deosaran *et al*, 2013), and the E3 ligases responsible for tagging peroxisomes with ubiquitin were later found to be the peroxisome-localised PEX2 (Sargent *et al*, 2016) as well as MARCH5 (Zheng *et al*, 2021). Interestingly, MARCH5 is also a mitochondrially localised E3, where it has been shown to mediate mitochondrial dynamics (Karbowski *et al*, 2007; Sugiura *et al*, 2013; Xu *et al*, 2016). Also of relevance here is that USP30, an OMM-localised deubiquitylase, originally found to inhibit Parkin-mediated mitophagy (Bingol *et al*, 2014), can also localise to peroxisomes to counteract pexophagy in a similar manner (Marcassa *et al*, 2018; Riccio *et al*, 2019; Rusilowicz-Jones *et al*, 2020, 2022). These data not only show that ubiquitin plays a key role in mitophagy and pexophagy but also show that common proteins can act in both pathways. However, how and if mitophagy and pexophagy are coordinated under these conditions are not clear, particularly given that the stimuli to induce mitophagy (mitochondrial depolarisation) is distinct from that to induce pexophagy (mTOR inhibition).

Specific autophagy can also occur in the absence of cargo ubiquitin priming, with well-characterised examples occurring during mitophagy. Here, several OMM proteins have been shown to act as mitophagic receptors that directly recruit the autophagy machinery, thus bypassing ubiquitin priming (e.g. NIX, BNIP3, FUNDC1 and BCL2L13; Montava-Garriga & Ganley, 2020). However, prior to this study, whether a similar ubiquitin-independent process occurs in mammalian pexophagy was not clear. For mitophagy, it has been described that these ubiquitin-independent receptors play a role in different contexts and stimuli, such as programmed mitophagy (during cell differentiation) or stress-induced mitophagy (during iron chelation or mitochondrial depolarisation), and given our data, this is also likely true for pexophagy. It is not yet clear why both ubiquitin-dependent and independent pathways exist, but it is possible that mitophagy and pexophagy occurring in these different contexts are responsible for the turnover of distinct subtypes or populations of mitochondria and peroxisomes. However, more work is needed to confirm this hypothesis.

Mitochondrial dysfunction is a feature of many diseases, including several neurodegenerative disorders such as Alzheimer's disease (AD), Parkinson's disease (PD), Huntington's disease (HD) and amyotrophic lateral sclerosis (ALS; Johri & Beal, 2012; Itoh *et al*, 2013). A wealth of evidence has revealed that structural and functional mitochondrial abnormalities, as well alteration of mitochondrial dynamics, are key factors in promoting neurodegeneration (Johri & Beal, 2012; Itoh *et al*, 2013), and it is clear to see how impaired mitophagy could contribute to this (Montava-Garriga & Ganley, 2020). Another less studied feature of age-related diseases, including neurodegenerative disorders, is peroxisomal dysfunction. The involvement of peroxisomes in these diseases has been largely neglected due to functional similarities between peroxisomes and mitochondria (Cipolla & Lodhi, 2017). Indeed, both organelles play major roles in cell metabolism, including fatty acid metabolism, and ROS production and scavenging. However, the respective contribution of mitochondria and peroxisome in these different processes (as well as their potential interconnection) remains unclear. In our study, we revealed that mitochondrial and peroxisomal turnover can occur together, with a block in mitophagy also hindering pexophagy (and potentially vice versa). Thus, dysfunction in both these organelles could contribute to disease pathology. Given this, it may prove beneficial to analyse specific autophagy pathways not in isolation but in combination, especially when monitoring the consequences of impaired mitophagy.

Materials and Methods

Antibodies and reagents

Primary antibodies used were as follows: anti-HIF1α (R&D system MAB1536, 1:1,000 for WB), anti-FTH1 (CST #4393S, 1:1,000 for WB), anti-NCOA4 (Bethyl Laboratories A302-272A, 1:1,000 for WB), anti-HSP60 (CST #4870S, 1:1,000 for WB), anti-OMI (MRC PPU Regeants and Services, University of Dundee, 1:500 for WB), anti-catalase (Calbiochem 219010, 1:2,000 for WB, 1:1,000 for IF and R&D system MAB3398-S, 1:1,000 for IF), anti-PMP70 (Sigma P0497, 1:2,000 for WB, 1:1,000 for IF), anti-PEX5 (NOVUS NBP1-54585, 1:500 for WB and Proteintech 66309, 1:1,000 for WB), anti-PEX19 (Genetex GTX110721, 1:5,000 for WB), anti-LC3 A/B (CST #4108S, 1:1,000 for WB), anti-LC3 I/II (MBL M152-3, 1:500 for IF), anti-p62 (Abnova H00008878-M01, 1:10,000 for WB and MBL M162-3, 1:500 for IF), anti-vinculin (Abcam ab129002, 1:10,000 for WB), anti-ULK1 (CST #8054S, 1:1,000 for WB), anti-ATG13 (Sigma SAB4200100, 1:1,000 for WB), anti-NIX (CST #12396, 1:1,000 for WB and 1:500 for IF), anti-COXIV (CST #4850S, 1:1,000 for WB), anti OPA1 (BD Biosciences 612606, 1:1,000 for WB), anti-BNIP3 (CST #3769S and CST #44060, 1:1,000 for WB), anti-LAMP1 (Santa Cruz sc-20011, 1:500 for IF), anti-GAPDH (Proteintech 60004-1-Ig, 1:10,000 for WB), anti-ATPB (Abcam ab14730, 1:500 for IF), anti-Ubiquitin (FK2) (Enzo life sciences BML-PW8810-0, 1:200 for IF and 1:1,000 for WB), anti-P-Ubiquitin (S65) (CST #62802S, 1:1,000 for WB), anti-GFP (Roche, 11814460001, 1:1,000), anti-MHC (RD system MAB4470, 1:500 for WB), anti-Troponin T (Thermo Fisher MA5-12960, 1:500 for WB), anti-Band3 (BRIC 71, IBGRL, Filton, Bristol, UK), mouse IgG1 control antibody (BioLegend), APC anti-mouse IgG1 secondary antibody (BioLegend), FITC CD49d Antibody (Miltenyi Biotec) and FITC isotype control antibody (Miltenyi Biotec).

Secondary antibodies used were as follows: goat anti-Rabbit IgG (H+L) HRP conjugate, goat anti-mouse IgG (H+L) HRP

conjugate and Donkey anti-sheep IgG (H+L) HRP conjugate were purchased from Thermo Scientific (#31460, #31430, #A16041, respectively, 1:5,000 for WB).

3-Hydroxy-1,2-dimethyl-4(1H)-pyridone (DFP) (Sigma 379409), Bafilomycin A1 (Enzo BML-CM110), Ponceau S (Sigma P3504), OligomycinA (Sigma 75351), AntimycinA (Sigma A8674), Carbonyl cyanide 3-chlorophenylhydrazone (CCCP) (Sigma C2759), Ivermectin (Sigma I8898), 1,10-Phenanthroline (Sigma, 131377).

Cell culture, transfections and infections

ARPE-19 (ATCC, CRL-2302) and SH-SY5Y (ATCC, CRL-2266) cells were maintained in 1:1 DMEM:F-12 media (Thermo Scientific) supplemented with 15% (v/v) FBS, 2 mM L-glutamine, 100 U/ml penicillin and 0.1 mg/ml streptomycin. HeLa and 293FT cells were cultured in DMEM with supplemented with 10% (v/v) FBS, 2 mM L-glutamine, 100 U/ml penicillin and 0.1 mg/ml streptomycin. H9c2 cardiomyoblasts (ECACC, 88092904) were cultured in DMEM with supplemented with 10% (v/v) FBS, 2 mM L-glutamine, 100 U/ml penicillin and 0.1 mg/ml streptomycin and MEM Non-Essential Amino Acids. All cell lines were confirmed mycoplasma negative using MycoAlert Detection kit (Lonza, LT07-318). Cells were split when they reached 70–80% confluency to avoid spontaneous differentiation into cardiomyocytes. Differentiation was induced by changing the medium to DMEM with 1% (v/v) FBS, 100 U/ml penicillin and 0.1 mg/ml streptomycin and 10 nM all-trans-Retinoic acid (ATRA) for 8 days. Media were changed every 2 days. All cell lines were grown at 37°C with 5% CO2 in a water-saturated incubator.

For hypoxia treatment, cells were grown at 37°C with 0.8% O_2 and 5% CO_2 in a water-saturated incubator for 3 days.

For the knockdown of endogenous ATG13 and ULK1, 1.5×10^5 cells in suspension were mixed with 500 µl Opti-MEM, 2.5 µl Transfectin™ (Bio-rad) and 50 pmol siRNA oligonucleotides and plated in a six-well plates. After 7 h, the media were replaced with growth media. Cells were typically treated with DFP 48 h post-transfection and harvested 96 h post-transfection.

Stable cell lines were generated by retroviral infections. To produce retroviral particles, 293FT cells at 60–70% of confluency, were cotransfected directly in the growth media with X-tremeGENE 9 DNA Transfection Reagent (Roche) ratio 3:1, the cDNA of interest, GAG/POL and VSVG vectors (Clontech). Virus was harvested 48 h post-transfection and applied to cells in the presence of 10 µg/ml polybrene and 20 mM HEPES. Cells were selected with 500 µg/ml hygromycin (Source Bioscience) or 2 µg/ml puromycin (Sigma) after 48 h post transduction. Stable pools were used for experiments.

The CRISPR/Cas9 system was used to generate NIX KO CRISPR cells. Plasmids used for the expression of the Cas9 D10A nuclease and paired guide RNAs (gRNAs) targeting exon 2 of the *Nix* gene were designed by Thomas Macartney at the MRC PPU Reagents and Services (gRNA sense: GTTGTGGATGGAGGATGAGGA, gRNA antisense: GAGACATGGAGAAGATTCTTT). The targeting gRNAs were cotransfected into cells using 23-polyethylenimine (PEI, Sigma-Aldrich). One-day post-transfection selection with 2 µg/ml puromycin was carried out for 48 h. After a recovery period, cells were diluted and plated to allow for the isolation of single colonies. Colonies were expanded before immunoblotting analysis and DNA sequencing.

U2OS HIF1 KO cells were generated as previously described in Zhao *et al* (2020).

siRNA and plasmids

Sequences of siRNAs used in this manuscript were as follows: Non-targeting siRNA oligo (Dharmacon, 5′-UGGUUUACAUGUCGACUAA UU-3′), ATG13 (Ambion by life technologies, 5′-CGGUGUACAA CAGACUGUCtt), ULK1 (Qiagen, 5′-CGCGGUACCUCCAGAGCAATT-3′), NIX (Life Technologies, 5′-CCAUAGCUCUCAGUCAGAATT-3′), BNIP3 (Life Technologies, 5′-GAAAAACUCAGAUUGGAUA-3′).

The retroviral expression vector for *mito*-QC was previously described (Allen *et al*, 2013). To generate pBabe mCherry-GFP-SKL, the vector pBabe mCherry-GFP-PEX26 (S245-P274) from DSTT, University of Dundee (DU45642), was modified with the In-fusion HD cloning Kit (Takarabio). Vectors pBabeD Flag-NIX, pBabeD Flag, pBabeD GFP-NIX, pBabe GFP-BNIP3, pQCXIP HA-Parkin were generated by the DSTT, University of Dundee (DU49760, DU37214, DU40155, DU40766, DU66655, respectively). To generate pBabeD GFP-NIX ΔTM, pBabeD GFP-NIX TM only, pBabeD Flag-NIX W36A \L39A, pBabeD Flag-NIX S34E\S35E and pBabeD Flag NIX G204A, the In-fusion HD cloning Kit was used (Takarabio) (DU75173, DU75172, DU75171, DU75169, DU75168, respectively). All plasmids are available at the following address https://mrcppureagents.dundee.ac.uk.

Preparation cell lysates and Western blot analysis

Cultured cells were lysed with HEPES lysis buffer (40 mM HEPES, 2 mM EDTA, 1% triton) supplemented with cOmplete, EDTA-free—Protease Inhibitor Cocktail (Roche) and phosphatase inhibitor cocktail (115 mM sodium molybdate, 400 mM sodium tartrate dihydrate, 1 M β-glycerophosphoric acid disodium salt, pentahydrate, 100 mM sodium fluoride, 100 mM activated sodium orthovanadate). Equal amounts of protein (around 10–15 µg) were resolved by 6–14% Bis Tris gels, transferred to nitrocellulose membrane (Amershan Protan 0.45 µm nitrocellulose membrane), blocked in 2% milk in TBS supplemented with Tween-20 and probed with primary antibody overnight at 4°C. After washes with TBS-Tween, membranes were subsequently incubated with HRP-conjugated secondary antibody for 1 h at room temperature. The signal detection was performed using ECL (Biorad, Clarity Western ECL Substrate) and Biorad Chemidoc imager.

Immunofluorescence

Cells were seeded onto sterile glass coverslips in 24-well dishes. Coverslips were washed once with PBS, fixed with 3.7% (w/v) formaldehyde, 200 mM HEPES pH 7.0 for 10 min and washed twice with PBS. Cells were permeabilized with 0.1% triton in PBS for 4 min. After two washes with PBS, samples were blocked by incubation for 30 min in blocking buffer (1% (w/v) BSA/PBS). Coverslips were incubated for 2 h at 37°C with primary antibodies in blocking buffer and washed three times in PBS. Coverslips were then incubated for 1 h at room temperature with Alexafluor

 © 2022 The Authors

coupled secondary antibodies (Life Technologies) in blocking buffer and washed an additional three times in PBS. If needed, nuclei were counterstained with 1 μg/ml Hoechst-33258 dye (Sigma) for 5 min, and slides were washed twice with PBS and mounted in ProLong Gold (Invitrogen). Observations were made with Nikon Eclipse Ti widefield microscope (Plan Apo Lambda 60× Oil Ph3 DM) or a Zeiss LSM880 Airyscan Confocal Scanning microscope (ZEISS, 63× objective, NA 1.4). Images were processed using ImageJ and Adobe Photoshop Software.

Mitophagy and pexophagy assays

Microscopy analysis

Cells stably expressing *mito*-QC mitophagy reporter system (mCherry-GFP-FIS1$^{101–152}$) or *pexo*-QC pexophagy reporter system (mCherry-GFP-SKL) were seeded onto sterile glass coverslips in 24-well dishes. After treatment, coverslips were washed twice with PBS, fixed with 3.7% (w/v) formaldehyde, 200 mM HEPES pH 7.0 for 10 min and washed twice with PBS. After nuclei counterstaining with 1 μg/ml Hoechst-33258 dye, slides were washed and mounted in ProLong Gold (Invitrogen). For quantification, images were taken with Nikon Eclipse Ti widefield microscope (Plan Apo Lambda 60× Oil Ph3 DM). All the images were processed with FIJI v1.52n software (ImageJ, NIH). Quantification of mitophagy or pexophagy was performed from three independent experiments counting over 40 cells per condition. Images were processed with the mito-*QC* Counter as previously described (Montava-Garriga *et al*, 2020). For pexophagy, a Gaussian blur was used prior the semi-automated quantitation. As quantitation was automated, initial blinding to sample ID was not performed.

Flow cytometry analysis

1.75×10^5 cells were seeded in a 6 cm dish. After treatment, cells were washed with PBS, trypsinized for 5 min and centrifuged 3 min at 1,200 rpm. The pellet of cells was resuspended in 250 μl of PBS and 2 ml of 3.7% (w/v) formaldehyde, 200 mM HEPES pH 7.0 were added. After 10 min on ice followed by 20 min at RT, 3 ml of PBS was added before centrifugation 5 min at 1,200 rpm. Finally, the pellet of cells was resuspended in 1% FCS in PBS and analysed by flow cytometry. For each independent experiment, at least 2×10^4 cells were acquired on LSRFortessa cell analyser. Based on FCS and SSC profiles, living cells were gated. As negative control, cells expressing any mitophagy or pexophagy reporter were used. To quantify the percentage of cells underdoing mitophagy or pexophagy, the ratio GFP/mCherry was analysed. The gate used for the nontreated condition or control cells was applied to all the other conditions. The value used for this was based on quantitation of microscopy data from *mito*-QC/*pexo*-QC cells that showed 7% (*mito*-QC) or 11% (*pexo*-QC) of cells had red-only puncta above the value of the mean.

Mitochondria depletion

As previously described (Correia-Melo *et al*, 2017), ARPE-19 cells overexpressing HA-Parkin were treated with 20 μM CCCP diluted in media for 24 h, washed with PBS and cultured in normal media for 24 h, prior DFP treatment. Finally, cells were lysed or fixed for Western blot analysis and immunofluorescence.

Colocalisation analysis

Colocalisation coefficient (Pearson correlation) was determined using Velocity 6.3 Image Analysis software (PerkinElmer). Quantification of colocalisation was performed from three independent experiments counting over 15 cells per condition.

Erythrocyte differentiation

Primary human erythroid cell cultures

Primary human erythroid cells were cultured as previously described (Griffiths *et al*, 2012; Kupzig *et al*, 2017). Waste blood from anonymous platelet apheresis donors (NHSBT, Filton, Bristol, UK) were provided with written informed consent for research use, given in accordance with the Declaration of Helsinki and approved by local Research Ethics Committee (Southmead Research Ethics Committee reference 08/H0102/26 and Bristol Research Ethics Committee Centre reference 12/SW/0199). On Day 0, $CD34^+$ cells were isolated from peripheral blood mononuclear cells (PBMCs) by positive selection using CD34 magnetic MicroBeads and the Magnetic Activated Cell Sorting system (MiniMACS) according to the manufacturer's instructions (Miltenyi Biotech) and seeded at a density of 2×10^5 cells/ml in base medium [Iscove's modified Dulbecco's medium (Biochrom IMDM, Merck) containing 3% (v/v) AB Serum (Merck), 2 mg/ml HSA (Irvine Scientific), 10 μg/ml Insulin (Merck), 3 U/ml heparin (Merck), 500 μg/ml holo-transferrin (BBI solutions) and 3 U/ml Epo (NeoRecormon, Roche)] supplemented with 10 ng/ml Stem Cell Factor (SCF, Miltenyi Biotec) and 1 ng/ml interleukin 3 (Il-3, BioTechne). Cells were counted daily from day 3 onwards and fed by medium addition. From days 0–10, 10 ng/ml SCF and 1 ng/ml Il-3 were used; from days 11–13, the medium was only supplemented with 10 ng/ml SCF and from days 14–20, base medium only was used. On day 20, the erythroid cell cultures were gravity filtered using a 5 μm Acrodisc syringe filter (Pall) to remove free nuclei (pyrenocytes) and nucleated precursors, and obtain reticulocytes collected from the flow through.

Western blot analysis

Cells (6×10^6) were pelleted on days 7, 10, 13 and snap frozen in liquid nitrogen. On day 20, after filtration, 6×10^6 filtered reticulocytes were pelleted and snap frozen in liquid nitrogen. Samples were stored at −80°C until use. Cells were lysed for 10 min on ice in lysis buffer (20 mM Tris–HCl, pH 8.0, 137 mM NaCl, 10 mM EDTA, 100 mM NaF, 1% (v/v) Nonidet P-40, 10% (v/v) glycerol, 10 mM Na3VO4, 2 mM PMSF and protease inhibitors, Calbiochem), followed by centrifugation at 4°C at 16,000 *g* for 10 min.

Cytospins

To analyse cell morphology on days 7, 10, 13 and 20, 2×10^5 erythroid cells or filtered reticulocytes were cytospun onto glass slides using a Shandon Cytospin 4 Cytocentrifuge with cytofunnels and cytoclips (Thermo Scientific), air dried for 1 min and fixed in methanol for 15 min. The slides were then stained with May-Grünwald's stain (Merck, diluted 1/2 in Sorensen buffered water) for 5 min, with Giemsa's stain (Merck, diluted 1/10 in Sorensen buffered water) for 10 min and left in Sorensen buffered water for 3 min. Air dried cytospins were imaged using a 40× lens on an

© 2022 The Authors

Olympus CX43 microscope coupled to an Olympus SC50 camera and the cellSens Entry software.

Flow cytometry

Cells taken from the cultures on days 7, 10, 13 and 20 were analysed for cell surface expression of Band 3 (CD233) and alpha 4 integrin (CD49d) to monitor erythroid cell differentiation as described by Hu *et al* (2013). The antibodies used were BRIC 71 (mouse IgG1 anti-Band 3 antibody, IBGRL, Filton, Bristol, UK), mouse IgG1 control antibody (BioLegend), APC anti-mouse IgG1 secondary antibody (BioLegend), FITC CD49d Antibody (Miltenyi Biotec) and FITC isotype control antibody (Miltenyi Biotec). For each flow cytometry test, 1×10^5 cells were washed in PBSAG [PBS containing 1 mg/ml bovine serum albumin (BSA) and 2 mg/ml glucose] and labelled with primary antibody diluted in PBSAG + 1% (w/v) BSA. Cells were washed in PBSAG, incubated with the APC-conjugated secondary antibody and washed in PBSAG. Data were acquired on a MacsQuant VYB Analyser (Miltenyi Biotec) using a plate reader and analysed using the FlowJo software. To reduce antibody binding-induced agglutination, day 20 cells were stained with 5 µg/ml Hoechst 33342 (Invitrogen) then fixed in 1% paraformaldehyde, 0.0075% glutaraldehyde in PBSAG before labelling with antibodies as described above. Reticulocytes were identified by gating upon the Hoechst-negative population.

In vivo experiments

Animals

All animal procedures and study protocols were approved by the local ethics committee for animal experimentation and the ethics committees of the CSIC and were carried out in accordance EU regulations and the ARVO Statement for the Use of Animals in Ophthalmic and Vision Research. NIX/BNIP3L mice were kindly provided by Prof. Gerald W. Dorn, II, Washington University in St. Louis (Diwan *et al*, 2007). Both male and female animals at 3–4 months of age were used in this study. Mice were reared in a barrier-controlled facility (20°C; 12-h light/dark cycle) with *ad libitum* access to food and water. Animals were euthanized by cervical dislocation. Eyes were removed under a dissecting microscope and fixed for 1 h in 4% PFA (w/v).

Immunofluorescence staining, microscopy & analysis

Fixed eyes were cryopreserved in 15% sucrose in 1× PBS overnight, followed by 30% sucrose for 1 week. Eyes were embedded in Tissue-Tek OCT (Sakura Finetechnical Co. Ltd., Tokyo, Japan) and sectioned at 12 µm on a LEICA CM 1950 cryostat. Eye cryosections were re-fixed with formaldehyde 4% (w/v) for 5 min, followed by three times 5 min PBS washes with agitation at room temperature. Retinal sections were permeabilized with 0.3% (v/v) Triton X-100 (Sigma-Aldrich, T8787) in PBS for 30 min and then blocked for 1 h with BGT [3 mg/ml BSA (Roche, 10735108001), 0.25% Triton X-100, 100 mM Glycine (VWR Chemicals, 0167) in PBS]. Primary antibodies were incubated in BGT (1:100) overnight at 4°C. Secondary antibodies were incubated for 1 h at room temperature in BGT (1:200) and darkness. Nuclei were stained with DAPI 1 µg/ml (ThermoFisher Scientific, D1306) and cryosections were mounted with Fluoromount (Bionova, 100-01) before sealing with nail polish and a 1.5 glass coverslip. Antibodies used were: PMP70 (Merck, P0497), Catalase (Merck, 219010) and LAMP1 (DSHB, 1D4B). Confocal images were taken with a confocal multispectral Leica TCS SP8 system. The interval between confocal planes was 1 µm. Maximum projections of the all z-stacks are displayed. PMP70, catalase and LAMP1 staining were quantified in ImageJ: maximum projections were performed and a minimum detection threshold common to all images was defined for positive area. Two to four different retinal regions have been measured to determine the mean of each mouse.

Statistics

Representative results of at least three independent experiments (biological replicates) are shown in all panels. Data are presented as mean and standard deviations (s.d.). For immunoblot quantifications, level of each protein was normalised to vinculin or GAPDH and expressed as fold change. GraphPad Prism software was used for all statistical analyses. Statistical significance was determined using unpaired Student's *t*-test for two group comparisons and one-way ANOVA with Dunnett's multiple comparison test or Tukey's multiple comparison test for comparing the means of > 2 groups. For multiple treatments, significance was determined by two-way ANOVA with Sidak's multiple comparisons test or Tukey's multiple comparisons test. *P*-values are indicated as $^*P < 0.05$, $^{**}P < 0.01$, $^{***}P < 0.001$ and $^{****}P < 0.0001$. ns: $P > 0.05$.

Data availability

This study includes no data deposited in external repositories.

Expanded View for this article is available online.

Acknowledgements

We would like to thank the Ganley lab for critical reading of this manuscript and Luke Simpson (MRC-PPU, University of Dundee) for generating the NIX KO clones. We acknowledge Paul Appleton at the Dundee Imaging Facility, Dundee and the Advanced Light Microscopy Service, headed by Ana Oña from CNB, and the technical staff at the animal facility of the CIB Margarita Salas for their support. The Zeiss LSM880 with Airyscan was supported by the "Wellcome Trust Multi-User Equipment Grant" (208401/Z/17/Z). We also acknowledge Prof. Gerald W. Dorn II, Washington University in St. Louis that kindly provided the NIX/BNIP3L tissues. We thank the technical support of the MRC PPU, including the MRC PPU tissue culture team (coordinated by Edwin Allen) and MRC PPU Reagents and Services, especially Thomas Macartney for his help in designing the CRISPR/Cas9 KO cell lines. We also thank Adrien Rousseau, MRC PPU Dundee, for all his help especially for the cloning. We acknowledge the group of prof. Ron Hay, GRE University of Dundee, for the hypoxia incubator. This work was funded by an EMBO Long-Term Fellowship (LPW; ALTF 1077-2018), a grant from the Medical Research Council, UK (IGG; MC_UU_00018/2) and by Ministerio de Ciencia, Innovación y Universidades (MCIU), Agencia Estatal de Investigación (AEI) and Fondo Europeo de Desarrollo Regional (FEDER) PGC2018-098557-B-I00 to PB. JZ-M holds a FPU fellowship from The Ministry of Universities, Spain. AMT was supported in part by the National Institute for Health Research Blood and Transplant Research Unit (NIHR BTRU) in Red Cell Products (IS-BTU-1214-10032), NHSBT R&D funding (WP-15-05), the European Union ITN "EVIDENCE" grant agreement ID 860436. The views expressed are those of

 © 2022 The Authors

the authors and not necessarily those of the National Health Service, NIHR, or the Department of Health and Social Care.

Author contributions

Léa P Wilhelm: Conceptualization; formal analysis; investigation; methodology; writing – original draft; writing – review and editing. **Juan Zapata-Muñoz:** Investigation; methodology. **Beatriz Villarejo-Zori:** Investigation; methodology. **Stephanie Pellegrin:** Formal analysis; investigation; writing – original draft. **Catarina Martins Freire:** Investigation. **Ashley M Toye:** Resources; formal analysis; supervision; funding acquisition; writing – original draft; writing – review and editing. **Patricia Boya:** Resources; supervision; funding acquisition; investigation; methodology; writing – original draft; writing – review and editing. **Ian G Ganley:** Conceptualization; resources; formal analysis; supervision; funding acquisition; investigation; visualization; methodology; writing – original draft; project administration; writing – review and editing.

Disclosure and competing interests statement

IGG is a consultant for Mitobridge Inc.

References

Allen GFG, Toth R, James J, Ganley IG (2013) Loss of iron triggers PINK1/Parkin-independent mitophagy. *EMBO Rep* 14: 1127 – 1135

Asano T, Komatsu M, Yamaguchi-Iwai Y, Ishikawa F, Mizushima N, Iwai K (2011) Distinct mechanisms of ferritin delivery to lysosomes in iron-depleted and iron-replete cells. *Mol Cell Biol* 31: 2040 – 2052

Aviram I, Shaklai N (1981) The association of human erythrocyte catalase with the cell membrane. *Arch Biochem Biophys* 212: 329 – 337

Bingol B, Tea JS, Phu L, Reichelt M, Bakalarski CE, Song Q, Foreman O, Kirkpatrick DS, Sheng M (2014) The mitochondrial deubiquitinase USP30 opposes Parkin-mediated mitophagy. *Nature* 510: 370 – 375

Braschi E, Goyon V, Zunino R, Mohanty A, Xu L, McBride HM (2010) Vps35 mediates vesicle transport between the mitochondria and peroxisomes. *Curr Biol* 20: 1310 – 1315

Braverman NE, Raymond GV, Rizzo WB, Moser AB, Wilkinson ME, Stone EM, Steinberg SJ, Wangler MF, Rush ET, Hacia JG *et al* (2016) Peroxisome biogenesis disorders in the Zellweger spectrum: an overview of current diagnosis, clinical manifestations, and treatment guidelines. *Mol Genet Metab* 117: 313 – 321

Cipolla CM, Lodhi IJ (2017) Peroxisomal dysfunction in age-related diseases. *Trends Endocrinol Metab* 28: 297 – 308

Correia-Melo C, Ichim G, Tait SWG, Passos JF (2017) Depletion of mitochondria in mammalian cells through enforced mitophagy. *Nat Protoc* 12: 183 – 194

Deosaran E, Larsen KB, Hua R, Sargent G, Wang Y, Kim S, Lamark T, Jauregui M, Law K, Lippincott-Schwartz J *et al* (2013) NBR1 acts as an autophagy receptor for peroxisomes. *J Cell Sci* 126: 939 – 952

Diwan A, Koesters AG, Odley AM, Pushkaran S, Baines CP, Spike BT, Daria D, Jegga AG, Geiger H, Aronow BJ *et al* (2007) Unrestrained erythroblast development in Nix−/− mice reveals a mechanism for apoptotic modulation of erythropoiesis. *Proc Natl Acad Sci USA* 104: 6794 – 6799

Egan DF, Shackelford DB, Mihaylova MM, Gelino S, Kohnz RA, Mair W, Vasquez DS, Joshi A, Gwinn DM, Taylor R *et al* (2011) Phosphorylation of ULK1 (hATG1) by AMP-activated protein kinase connects energy sensing to mitophagy. *Science* 331: 456 – 461

Esteban-Martinez L, Sierra-Filardi E, McGreal RS, Salazar-Roa M, Marino G, Seco E, Durand S, Enot D, Grana O, Malumbres M *et al* (2017) Programmed mitophagy is essential for the glycolytic switch during cell differentiation. *EMBO J* 36: 1688 – 1706

Gatica D, Lahiri V, Klionsky DJ (2018) Cargo recognition and degradation by selective autophagy. *Nat Cell Biol* 20: 233 – 242

Geng G, Liu J, Xu C, Pei Y, Chen L, Mu C, Wang D, Gao J, Li Y, Liang J *et al* (2021) Receptor-mediated mitophagy regulates EPO production and protects against renal anemia. *Elife* 10: e64480

Gong G, Song M, Csordas G, Kelly DP, Matkovich SJ, Dorn GW 2nd (2015) Parkin-mediated mitophagy directs perinatal cardiac metabolic maturation in mice. *Science* 350: aad2459

Griffiths RE, Kupzig S, Cogan N, Mankelow TJ, Betin VM, Trakarnsanga K, Massey EJ, Lane JD, Parsons SF, Anstee DJ (2012) Maturing reticulocytes internalize plasma membrane in glycophorin A-containing vesicles that fuse with autophagosomes before exocytosis. *Blood* 119: 6296 – 6306

Grumati P, Dikic I (2018) Ubiquitin signaling and autophagy. *J Biol Chem* 293: 5404 – 5413

Hanna RA, Quinsay MN, Orogo AM, Giang K, Rikka S, Gustafsson AB (2012) Microtubule-associated protein 1 light chain 3 (LC3) interacts with Bnip3 protein to selectively remove endoplasmic reticulum and mitochondria via autophagy. *J Biol Chem* 287: 19094 – 19104

Hara Y, Yanatori I, Tanaka A, Kishi F, Lemasters JJ, Nishina S, Sasaki K, Hino K (2020) Iron loss triggers mitophagy through induction of mitochondrial ferritin. *EMBO Rep* 21: e50202

Harper JW, Ordureau A, Heo JM (2018) Building and decoding ubiquitin chains for mitophagy. *Nat Rev Mol Cell Biol* 19: 93 – 108

Honda S, Arakawa S, Nishida Y, Yamaguchi H, Ishii E, Shimizu S (2014) Ulk1-mediated Atg5-independent macroautophagy mediates elimination of mitochondria from embryonic reticulocytes. *Nat Commun* 5: 4004

Hu J, Liu J, Xue F, Halverson G, Reid M, Guo A, Chen L, Raza A, Galili N, Jaffray J *et al* (2013) Isolation and functional characterization of human erythroblasts at distinct stages: implications for understanding of normal and disordered erythropoiesis *in vivo*. *Blood* 121: 3246 – 3253

Itoh K, Nakamura K, Iijima M, Sesaki H (2013) Mitochondrial dynamics in neurodegeneration. *Trends Cell Biol* 23: 64 – 71

Jin A, Lee JN, Kim MS, Kwak S, Kim S-J, Song K, Choe S-K, Park R (2016) 2,2′-dipyridyl induces pexophagy. *Biochem Biophys Res Commun* 469: 941 – 947

Jo DS, Bae D-J, Park SJ, Seo HM, Kim HB, Oh JS, Chang JW, Kim S-Y, Shin J-W, Cho D-H (2015) Pexophagy is induced by increasing peroxisomal reactive oxygen species in 1′10-phenanthroline-treated cells. *Biochem Biophys Res Commun* 467: 354 – 360

Jo DS, Park SJ, Kim A-K, Park NY, Kim JB, Bae J-E, Park HJ, Shin JH, Chang JW, Kim PK *et al* (2020) Loss of HSPA9 induces peroxisomal degradation by increasing pexophagy. *Autophagy* 16: 1989 – 2003

Johri A, Beal MF (2012) Mitochondrial dysfunction in neurodegenerative diseases. *J Pharmacol Exp Ther* 342: 619 – 630

Karbowski M, Neutzner A, Youle RJ (2007) The mitochondrial E3 ubiquitin ligase MARCH5 is required for Drp1 dependent mitochondrial division. *J Cell Biol* 178: 71 – 84

Kim PK, Hailey DW, Mullen RT, Lippincott-Schwartz J (2008) Ubiquitin signals autophagic degradation of cytosolic proteins and peroxisomes. *Proc Natl Acad Sci USA* 105: 20567 – 20574

Kirkin V (2020) History of the selective autophagy research: how did it begin and where does it stand today? *J Mol Biol* 432: 3 – 27

Kondapalli C, Kazlauskaite A, Zhang N, Woodroof HI, Campbell DG, Gourlay R, Burchell L, Walden H, Macartney TJ, Deak M *et al* (2012) PINK1 is activated by mitochondrial membrane potential depolarization and

stimulates Parkin E3 ligase activity by phosphorylating Serine 65. *Open Biol* 2: 120080

Kupzig S, Parsons SF, Curnow E, Anstee DJ, Blair A (2017) Superior survival of *ex vivo* cultured human reticulocytes following transfusion into mice. *Haematologica* 102: 476 – 483

Laker RC, Drake JC, Wilson RJ, Lira VA, Lewellen BM, Ryall KA, Fisher CC, Zhang M, Saucerman JJ, Goodyear LJ *et al* (2017) Ampk phosphorylation of Ulk1 is required for targeting of mitochondria to lysosomes in exercise-induced mitophagy. *Nat Commun* 8: 548

Lampert MA, Orogo AM, Najor RH, Hammerling BC, Leon LJ, Wang BJ, Kim T, Sussman MA, Gustafsson AB (2019) BNIP3L/NIX and FUNDC1-mediated mitophagy is required for mitochondrial network remodeling during cardiac progenitor cell differentiation. *Autophagy* 15: 1182 – 1198

Mancias JD, Wang X, Gygi SP, Harper JW, Kimmelman AC (2014) Quantitative proteomics identifies NCOA4 as the cargo receptor mediating ferritinophagy. *Nature* 509: 105 – 109

Marcassa E, Kallinos A, Jardine J, Rusilowicz-Jones EV, Martinez A, Kuehl S, Islinger M, Clague MJ, Urbé S (2018) Dual role of USP30 in controlling basal pexophagy and mitophagy. *EMBO Rep* 19: e45595

Marinković M, Šprung M, Novak I (2021) Dimerization of mitophagy receptor BNIP3L/NIX is essential for recruitment of autophagic machinery. *Autophagy* 17: 1232 – 1243

Mast FD, Rachubinski RA, Aitchison JD (2020) Peroxisome prognostications: exploring the birth, life, and death of an organelle. *J Cell Biol* 219: e201912100

Matsuda N, Sato S, Shiba K, Okatsu K, Saisho K, Gautier CA, Sou YS, Saiki S, Kawajiri S, Sato F *et al* (2010) PINK1 stabilized by mitochondrial depolarization recruits Parkin to damaged mitochondria and activates latent Parkin for mitophagy. *J Cell Biol* 189: 211 – 221

Mauro-Lizcano M, Esteban-Martinez L, Seco E, Serrano-Puebla A, Garcia-Ledo L, Figueiredo-Pereira C, Vieira HL, Boya P (2015) New method to assess mitophagy flux by flow cytometry. *Autophagy* 11: 833 – 843

McWilliams TG, Prescott AR, Allen GFG, Tamjar J, Munson MJ, Thomson C, Muqit MMK, Ganley IG (2016) mito-QC illuminates mitophagy and mitochondrial architecture *in vivo*. *J Cell Biol* 214: 333 – 345

McWilliams TG, Prescott AR, Montava-Garriga L, Ball G, Singh F, Barini E, Muqit MMK, Brooks SP, Ganley IG (2018) Basal mitophagy occurs independently of PINK1 in mouse tissues of high metabolic demand. *Cell Metab* 27: 439 – 449

McWilliams TG, Prescott AR, Villarejo-Zori B, Ball G, Boya P, Ganley IG (2019) A comparative map of macroautophagy and mitophagy in the vertebrate eye. *Autophagy* 15: 1296 – 1308

Mizushima N (2018) A brief history of autophagy from cell biology to physiology and disease. *Nat Cell Biol* 20: 521 – 527

Montava-Garriga L, Ganley IG (2020) Outstanding questions in mitophagy: what we do and do not know. *J Mol Biol* 432: 206 – 230

Montava-Garriga L, Singh F, Ball G, Ganley IG (2020) Semi-automated quantitation of mitophagy in cells and tissues. *Mech Ageing Dev* 185: 111196

Mortensen M, Ferguson DJP, Edelmann M, Kessler B, Morten KJ, Komatsu M, Simon AK (2010) Loss of autophagy in erythroid cells leads to defective removal of mitochondria and severe anemia *in vivo*. *Proc Natl Acad Sci USA* 107: 832 – 837

Murakawa T, Okamoto K, Omiya S, Taneike M, Yamaguchi O, Otsu K (2019) A mammalian mitophagy receptor, Bcl2-L-13, recruits the ULK1 complex to induce mitophagy. *Cell Rep* 26: 338 – 345

Narendra D, Tanaka A, Suen D-F, Youle RJ (2008) Parkin is recruited selectively to impaired mitochondria and promotes their autophagy. *J Cell Biol* 183: 795 – 803

Narendra D, Kane LA, Hauser DN, Fearnley IM, Youle RJ (2010a) p62/SQSTM1 is required for Parkin-induced mitochondrial clustering but not mitophagy; VDAC1 is dispensable for both. *Autophagy* 6: 1090 – 1106

Narendra DP, Jin SM, Tanaka A, Suen DF, Gautier CA, Shen J, Cookson MR, Youle RJ (2010b) PINK1 is selectively stabilized on impaired mitochondria to activate Parkin. *PLoS Biol* 8: e1000298

Neuspiel M, Schauss AC, Braschi E, Zunino R, Rippstein P, Rachubinski RA, Andrade-Navarro MA, McBride HM (2008) Cargo-selected transport from the mitochondria to peroxisomes is mediated by vesicular carriers. *Curr Biol* 18: 102 – 108

Ng MYW, Wai T, Simonsen A (2021) Quality control of the mitochondrion. *Dev Cell* 56: 881 – 905

Novak I, Kirkin V, McEwan DG, Zhang J, Wild P, Rozenknop A, Rogov V, Löhr F, Popovic D, Occhipinti A *et al* (2010) Nix is a selective autophagy receptor for mitochondrial clearance. *EMBO Rep* 11: 45 – 51

Riccio V, Demers N, Hua R, Vissa M, Cheng DT, Strilchuk AW, Wang Y, McQuibban GA, Kim PK (2019) Deubiquitinating enzyme USP30 maintains basal peroxisome abundance by regulating pexophagy. *J Cell Biol* 218: 798 – 807

Rocha S, Gomes D, Lima M, Bronze-da-Rocha E, Santos-Silva A (2015) Peroxiredoxin 2, glutathione peroxidase, and catalase in the cytosol and membrane of erythrocytes under H2O2-induced oxidative stress. *Free Radic Res* 49: 990 – 1003

Rodger CE, McWilliams TG, Ganley IG (2018) Mammalian mitophagy – from *in vitro* molecules to *in vivo* models. *FEBS J* 285: 1185 – 1202

Rogov VV, Suzuki H, Marinkovic M, Lang V, Kato R, Kawasaki M, Buljubasic M, Sprung M, Rogova N, Wakatsuki S *et al* (2017) Phosphorylation of the mitochondrial autophagy receptor Nix enhances its interaction with LC3 proteins. *Sci Rep* 7: 1131

Rosignol I, Villarejo-Zori B, Teresak P, Sierra-Filardi E, Pereiro X, Rodriguez-Muela N, Vecino E, Vieira HLA, Bell K, Boya P (2020) The mito-QC reporter for quantitative mitophagy assessment in primary retinal ganglion cells and experimental glaucoma models. *Int J Mol Sci* 21: 1882

Rusilowicz-Jones EV, Jardine J, Kallinos A, Pinto-Fernandez A, Guenther F, Giurrandino M, Barone FG, McCarron K, Burke CJ, Murad A *et al* (2020) USP30 sets a trigger threshold for PINK1-PARKIN amplification of mitochondrial ubiquitylation. *Life Sci Alliance* 3: e202000768

Rusilowicz-Jones EV, Barone FG, Lopes FM, Stephen E, Mortiboys H, Urbe S, Clague MJ (2022) Benchmarking a highly selective USP30 inhibitor for enhancement of mitophagy and pexophagy. *Life Sci Alliance* 5: e202101287

Sandoval H, Thiagarajan P, Dasgupta SK, Schumacher A, Prchal JT, Chen M, Wang J (2008) Essential role for Nix in autophagic maturation of erythroid cells. *Nature* 454: 232 – 235

Sargent G, van Zutphen T, Shatseva T, Zhang L, Di Giovanni V, Bandsma R, Kim PK (2016) PEX2 is the E3 ubiquitin ligase required for pexophagy during starvation. *J Cell Biol* 214: 677 – 690

Schrader M, Kamoshita M, Islinger M (2020) Organelle interplay-peroxisome interactions in health and disease. *J Inherit Metab Dis* 43: 71 – 89

Scott A, Fruttiger M (2010) Oxygen-induced retinopathy: a model for vascular pathology in the retina. *Eye (Lond)* 24: 416 – 421

Singh F, Ganley IG (2021) Parkinson's disease and mitophagy: an emerging role for LRRK2. *Biochem Soc Trans* 49: 551 – 562

Singh F, Prescott AR, Rosewell P, Ball G, Reith AD, Ganley IG (2021) Pharmacological rescue of impaired mitophagy in Parkinson's disease-related LRRK2 G2019S knock-in mice. *Elife* 10: e67604

 © 2022 The Authors

Smith JJ, Aitchison JD (2013) Peroxisomes take shape. *Nat Rev Mol Cell Biol* 14: 803 – 817

Sugiura A, Nagashima S, Tokuyama T, Amo T, Matsuki Y, Ishido S, Kudo Y, McBride HM, Fukuda T, Matsushita N *et al* (2013) MITOL regulates endoplasmic reticulum-mitochondria contacts via Mitofusin2. *Mol Cell* 51: 20 – 34

Sugiura A, Mattie S, Prudent J, McBride HM (2017) Newly born peroxisomes are a hybrid of mitochondrial and ER-derived pre-peroxisomes. *Nature* 542: 251 – 254

Tabak HF, Braakman I, Distel B (1999) Peroxisomes: simple in function but complex in maintenance. *Trends Cell Biol* 9: 447 – 453

Teresak P, Lapao A, Subic N, Boya P, Elazar Z, Simonsen A (2022) Regulation of PRKN-independent mitophagy. *Autophagy* 18: 24 – 39

Vives-Bauza C, Zhou C, Huang Y, Cui M, de Vries RLA, Kim J, May J, Tocilescu MA, Liu W, Ko HS *et al* (2010) PINK1-dependent recruitment of Parkin to mitochondria in mitophagy. *Proc Natl Acad Sci* 107: 378 – 383

Wangler MF, Hubert L, Donti TR, Ventura MJ, Miller MJ, Braverman N, Gawron K, Bose M, Moser AB, Jones RO *et al* (2018) A metabolomic map of Zellweger spectrum disorders reveals novel disease biomarkers. *Genet Med* 20: 1274 – 1283

Waterham HR, Ferdinandusse S, Wanders RJ (2016) Human disorders of peroxisome metabolism and biogenesis. *Biochim Biophys Acta* 1863: 922 – 933

Wu W, Tian W, Hu Z, Chen G, Huang L, Li W, Zhang X, Xue P, Zhou C, Liu L *et al* (2014) ULK1 translocates to mitochondria and phosphorylates FUNDC1 to regulate mitophagy. *EMBO Rep* 15: 566 – 575

Xu S, Cherok E, Das S, Li S, Roelofs BA, Ge SX, Polster BM, Boyman L, Lederer WJ, Wang C *et al* (2016) Mitochondrial E3 ubiquitin ligase MARCH5 controls mitochondrial fission and cell sensitivity to stress-induced apoptosis through regulation of MiD49 protein. *Mol Biol Cell* 27: 349 – 359

Zachari M, Ganley IG (2017) The mammalian ULK1 complex and autophagy initiation. *Essays Biochem* 61: 585 – 596

Zachari M, Gudmundsson SR, Li Z, Manifava M, Shah R, Smith M, Stronge J, Karanasios E, Piunti C, Kishi-Itakura C *et al* (2019) Selective autophagy of mitochondria on a ubiquitin-endoplasmic-reticulum platform. *Dev Cell* 50: 627 – 643

Zhang J, Ney PA (2009) Role of BNIP3 and NIX in cell death, autophagy, and mitophagy. *Cell Death Differ* 16: 939 – 946

Zhao J-F, Rodger CE, Allen GFG, Weidlich S, Ganley IG (2020) HIF1α-dependent mitophagy facilitates cardiomyoblast differentiation. *Cell Stress* 4: 99 – 113

Zheng J, Chen X, Liu Q, Zhong G, Zhuang M (2021) Ubiquitin ligase MARCH5 localizes to peroxisomes to regulate pexophagy. *J Cell Biol* 221: e202103156

License: This is an open access article under the terms of the Creative Commons Attribution License, which permits use, distribution and reproduction in any medium, provided the original work is properly cited.

Expanded View Figures

Figure EV1. Analysis of pexophagy upon iron chelation and hypoxia.

A Representative immunoblots of the indicated proteins in lysates of ARPE-19 cells treated 48 h with 1 mM DFP.

B, C Representative immunoblots of the indicated proteins in lysates of HeLa and SH-SY5Y cells (B) or ARPE-19 cells (C), treated with 1 mM DFP or 50 μM Phenentroline (Phen) for the indicated time (h).

D Representative immunoblots of the indicated proteins in lysates of ARPE-19 cells stimulated with hypoxia (0.8%) for 3 days.

E Representative confocal images of ARPE-19 cells stably expressing the *pexo*-QC reporter stimulated with hypoxia (0.8%) for 3 days and at right, quantification of total red-only punctate per cell (pexolysosomes). Enlarged images of the area outlined in white are shown in the lower corners.

F, G Representative confocal images of ARPE-19 cells stably expressing the *pexo*-QC reporter treated with 1 mM DFP for 48 h and immunostained with peroxisomal markers (F, Catalase and PMP70), or lysosomal marker (G, LAMP1). Enlarged images of the area outlined in white are shown on the right or below.

H, I ARPE-19 cells expressing the *pexo*-QC reporter were transfected with 50 pmol of non-targeting siRNA (siNT) or 50 pmol of siRNA targeting ATG13 or ULK1. 48 h post transfection, cells were treated with 1 mM DFP for an additional 48 h and analysed by immunoblot (H) or by flow cytometry (I). Representative dot plots are shown after analysing GFP and mCherry signals. The percentage of cell underdoing pexophagy (purple population) is indicated in bold purple on each dot plot.

Data information: Overall data are mean ± s.d.; $n = 3$ biological replicates. *$P \leq 0.05$; ****$P \leq 0.0001$; ns, not significant (two-way ANOVA, Sidak's multiple comparisons test). Nuclei were stained in blue (Hoechst) and scale bars: 10 μm.
Source data are available online for this figure.

 © 2022 The Authors

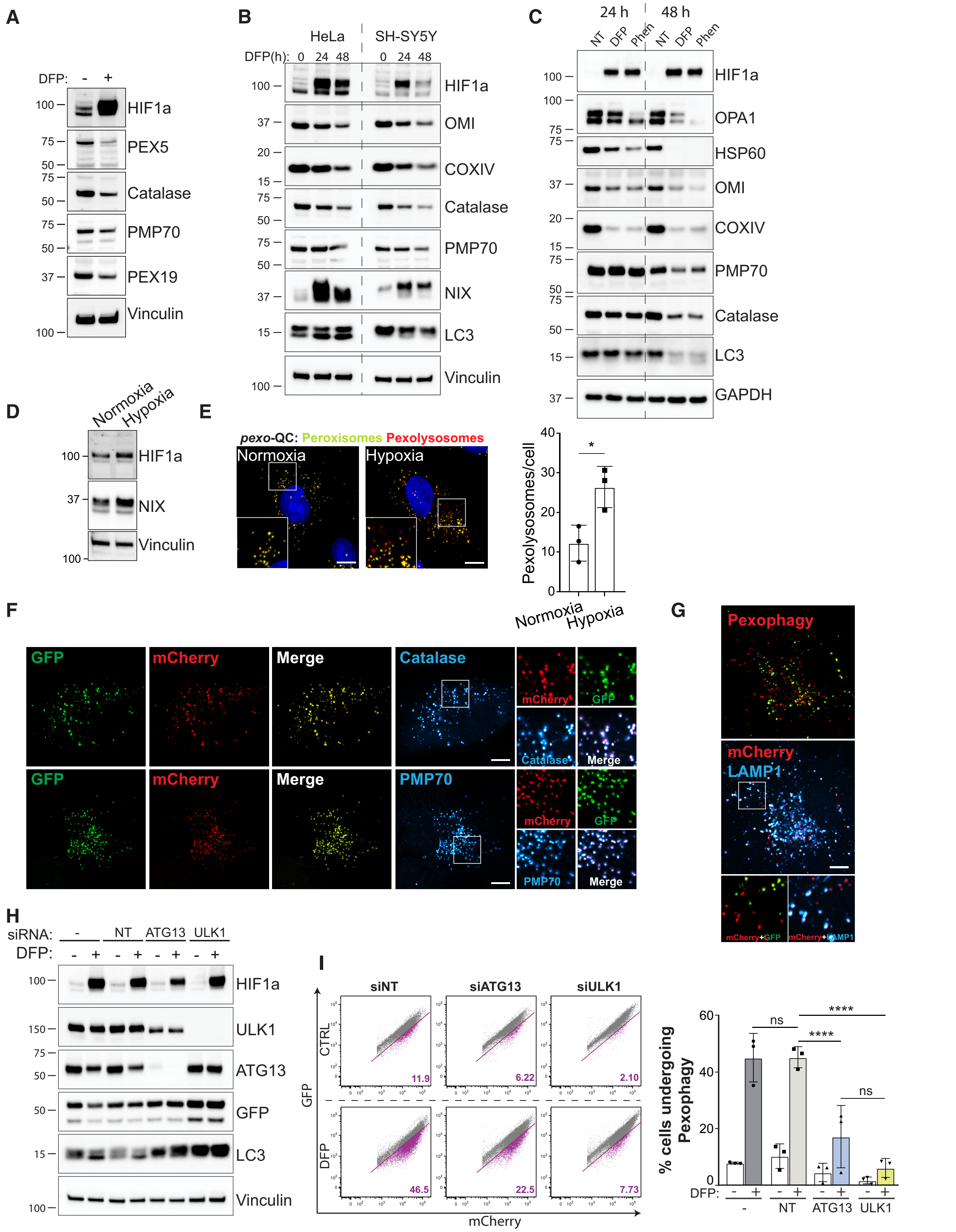

A
DFP: - +
HIF1a
PEX5
Catalase
PMP70
PEX19
Vinculin
B
HeLa
SH-SY5Y
DFP(h): 0 24 48 0 24 48
HIF1a
OMI
COXIV
Catalase
PMP70
NIX
LC3
Vinculin
C
24 h
48 h
NT DFP Phen NT DFP Phen
HIF1a
OPA1
HSP60
OMI
COXIV
PMP70
Catalase
LC3
GAPDH
D
Normoxia Hypoxia
HIF1a
NIX
Vinculin
E
pexo-QC: Peroxisomes Pexolysosomes
Normoxia
Hypoxia
Pexolysosomes/cell
F
GFP
mCherry
Merge
Catalase
PMP70
G
Pexophagy
mCherry
LAMP1
mCherry+GFP
mCherry+LAMP1
H
siRNA: - NT ATG13 ULK1
DFP: - + - + - + - +
HIF1a
ULK1
ATG13
GFP
LC3
Vinculin
I
siNT siATG13 siULK1
CTRL
DFP
GFP
mCherry
11.9 6.22 2.10
46.5 22.5 7.73
% cells undergoing Pexophagy
ns
****
****
ns
DFP: - + - + - + - +
- NT ATG13 ULK1

Figure EV1.

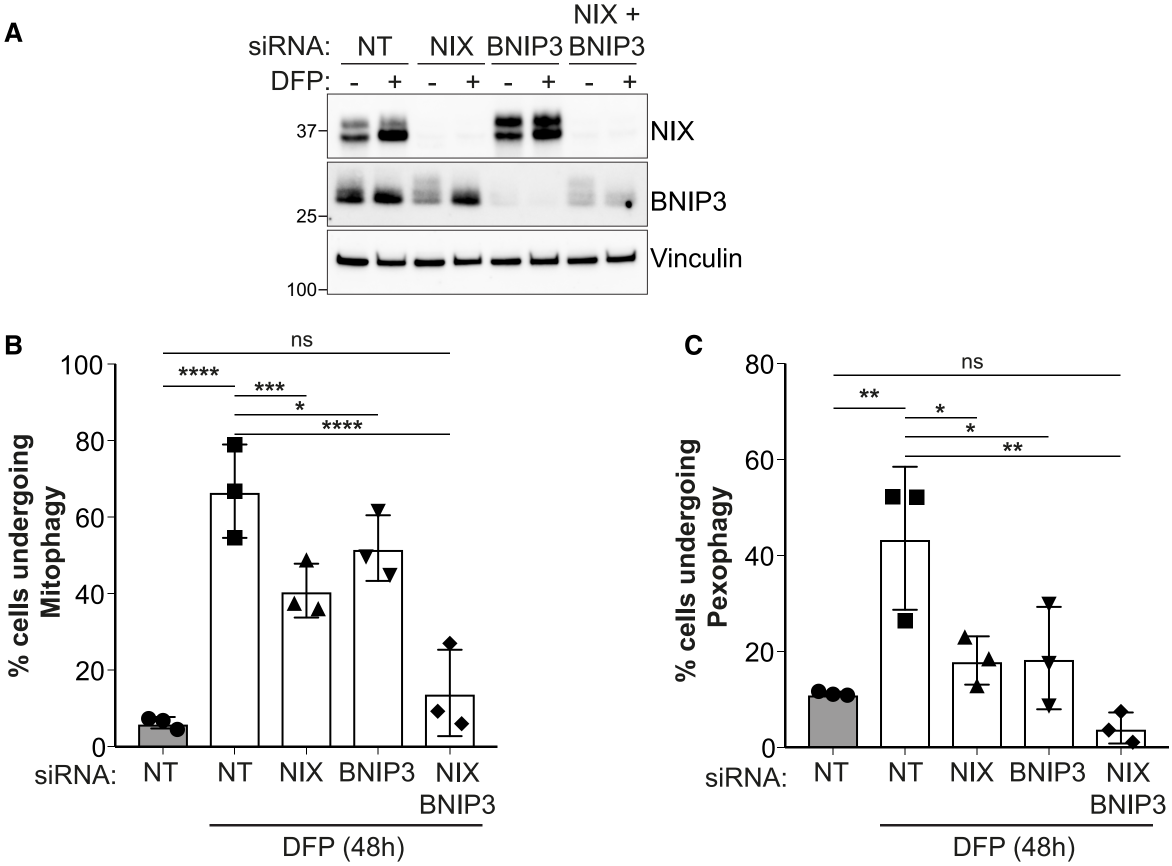

Figure EV2. BNIP3 is involved in mitophagy and pexophagy.

A Representative immunoblots of the indicated proteins in lysates of ARPE-19 cells transfected with 50 pmol of non-targeting siRNA (NT) or 50 pmol of siRNA targeting NIX or BNIP3. 48 h post transfection, cells were treated with 1 mM DFP for an additional 48 h.

B, C Quantification by flow cytometry of the mCherry/GFP ratio in ARPE-19 cells expressing the *mito*-QC reporter (B) or the *pexo*-QC reporter (C) and treated as in (A).

Data information: Overall data are mean ± s.d.; $n = 3$ biological replicates. *$P \le 0.05$; **$P \le 0.01$; ***$P \le 0.001$; ****$P \le 0.0001$; ns, not significant. One-way ANOVA, Tukey's multiple comparisons test.
Source data are available online for this figure.

Figure EV3. Analysis of NIX localisation.

A Representative confocal images of ARPE-19 cells immunostained with an anti-NIX antibody (green) and anti-catalase antibody (red) after 1 mM DFP for the indicated time (h). Peroxisomes enriched for NIX or having minimal signal for NIX are depicted with yellow or white arrowheads, respectively.

B Representative immunoblots and quantifications (below) of the indicated proteins in lysates of cells treated as (A). Data are mean ± s.d.; $n = 4$ biological replicates. *$P \le 0.05$; **$P \le 0.01$; ns, not significant (one-way ANOVA, Tukey's multiple comparisons test).

C Representative confocal images of ARPE-19 cells WT (right panel) or stably expressing GFP-NIX (left panel) and stimulated with hypoxia for 3 days. Peroxisomes were stained with anti-catalase antibody (red) and endogenous NIX were stained with anti-NIX antibody (green, right panel).

D Representative confocal images of untreated (CTRL) and DFP-treated ARPE-19 cells (1 mM, 48 h), stably expressing GFP-BNIP3 (green) and stained with anti-catalase antibody (red). At right, quantification of Pearson correlation coefficients between BNIP3 and the peroxisomal marker catalase after DFP treatment for 48 h.

E Schematic representation of the NIX mutants. Right panel, confocal images of ARPE-19 cells stably expressing GFP-NIX mutants and immunostained with a mitochondrial marker (ATPB, red).

Data information: Enlarged images of the area outlined in white are shown on the right. Nuclei were stained in blue (Hoechst) and scale bars: 10 μm.
Source data are available online for this figure.

▶

 © 2022 The Authors

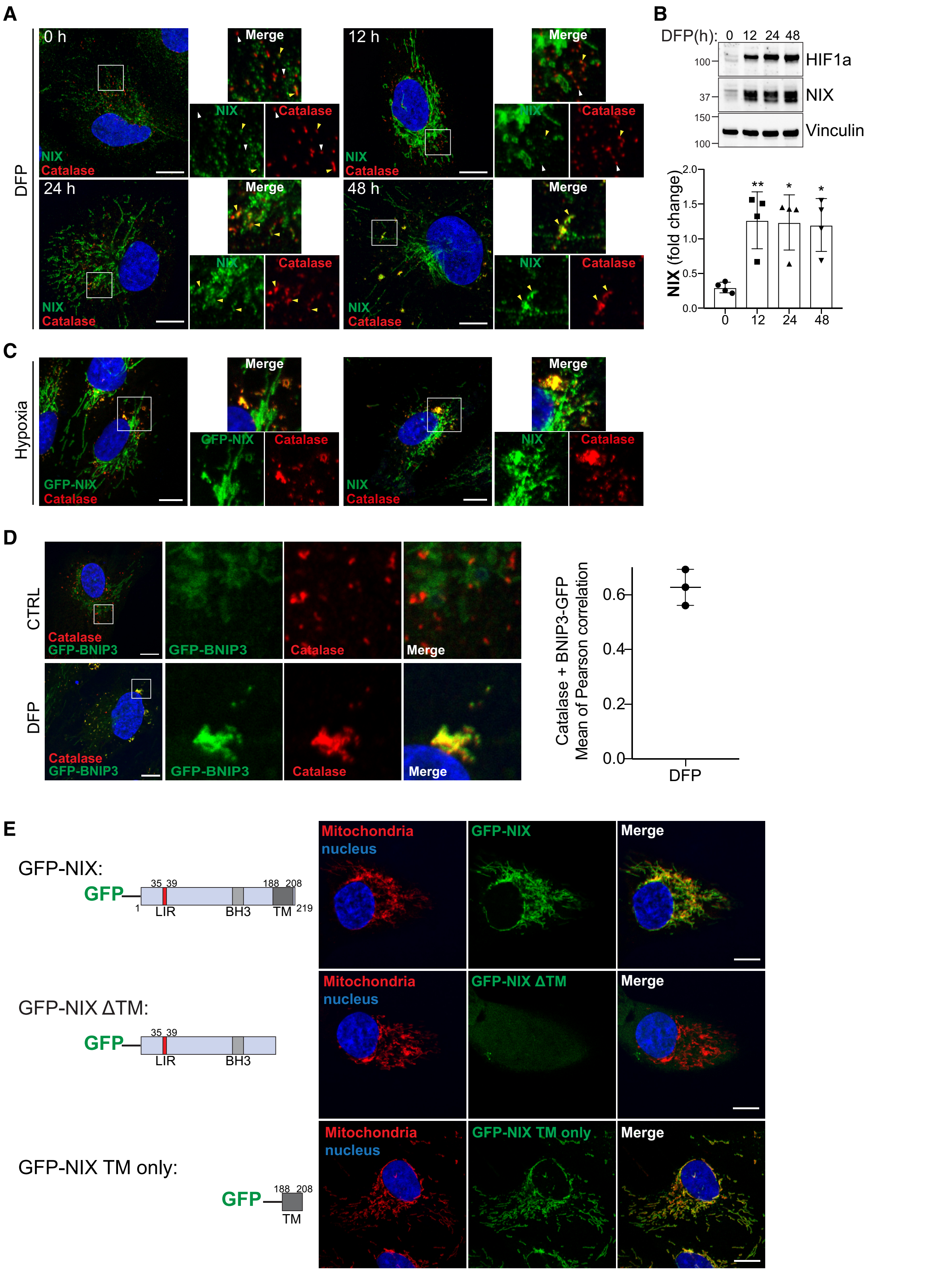
A
B
DFP
0 h
12 h
24 h
48 h
Merge
NIX
Catalase
DFP(h): 0 12 24 48
HIF1a
NIX
Vinculin
100
37
150
100
NIX (fold change)
2.0
1.5
1.0
0.5
0.0
0
12
24
48
**
*
*
C
Hypoxia
GFP-NIX
Catalase
Merge
NIX
D
CTRL
DFP
Catalase
GFP-BNIP3
Merge
Catalase + BNIP3-GFP
Mean of Pearson correlation
0.6
0.4
0.2
0.0
DFP
E
GFP-NIX:
GFP
35 39
188 208
1
LIR
BH3
TM
219
GFP-NIX ΔTM:
GFP-NIX TM only:
Mitochondria
nucleus
GFP-NIX
GFP-NIX ΔTM
GFP-NIX TM only
Merge

Figure EV3.

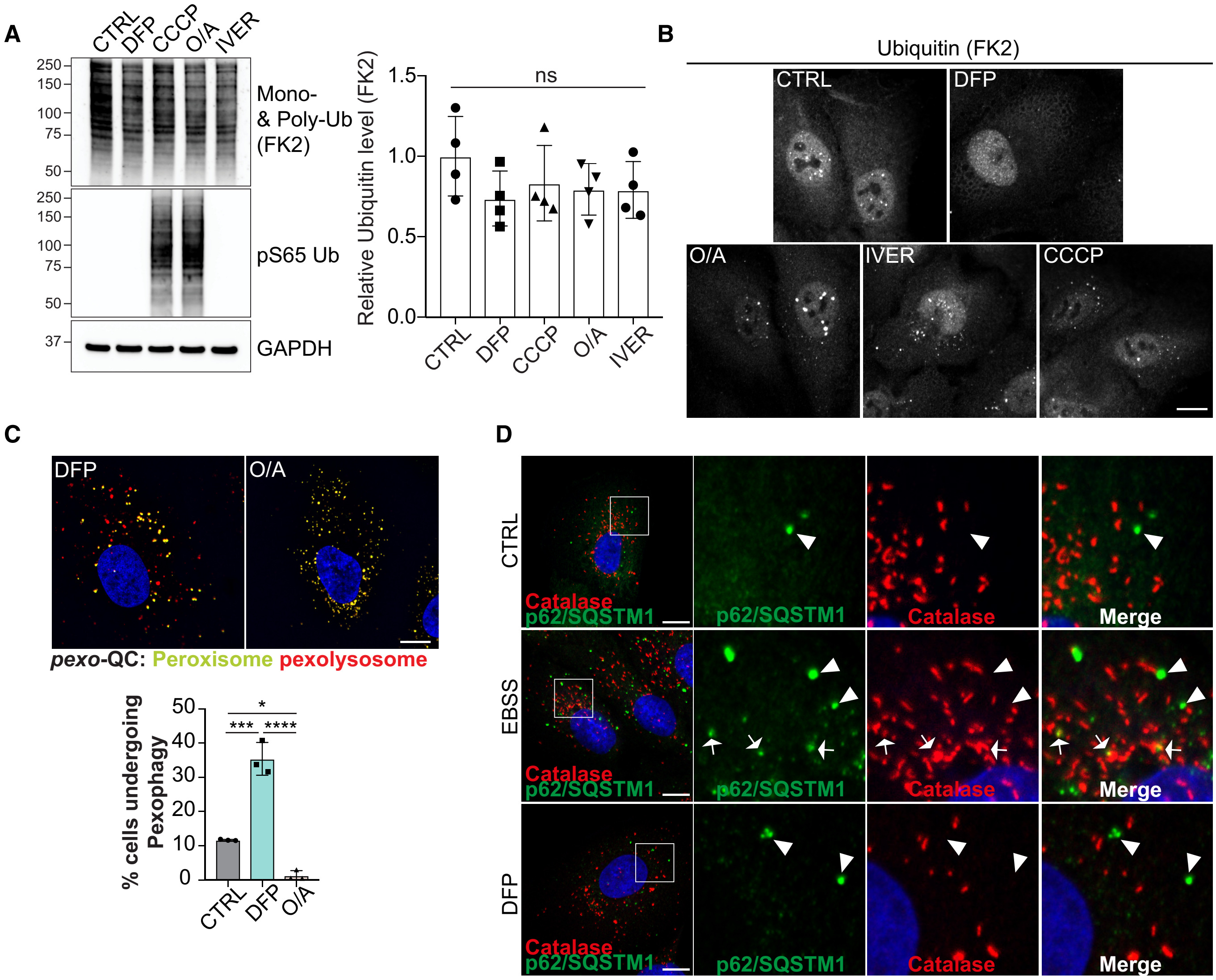

Figure EV4. Ubiquitylation status upon DFP treatment.

A Representative immunoblots and quantification of the indicated proteins in lysates of ARPE-19 cells treated with 1 mM DFP, 20 μM CCCP, 10 μM Antimycin and 5 μM Oligomycin A (O/A), 15 μM Ivermectin for 24 h.

B Representative confocal images of ARPE-19 cells treated as in A and stained with anti-Ubiquitin antibody (FK2).

C Representative confocal images of ARPE-19 cells stably expressing the *pexo*-QC reporter and treated with 1 mM DFP or 10 μM Antimycin and 5 μM Oligomycin A (O/A) for 48 h. Below, flow cytometry analysis of the mCherry/GFP ratio.

D Representative confocal images of ARPE-19 cells treated with EBSS for 24 h or 1 mM DFP for 48 h and immunostained with anti-catalase (red) and anti-p62 (green) antibodies. Autophagosomes containing peroxisomes are depicted with white arrows while autophagosomes not colocalizing with peroxisome marker are pointed with white arrowheads. Enlarged images of the area outlined in white are shown on the right.

Data information: Data are mean ± s.d.; Statistical significance is displayed as $*P \leq 0.05$; $***P \leq 0.001$; $****P \leq 0.0001$; ns, not significant. (A) $n = 4$ biological replicates, (C) $n = 3$ biological replicates; one-way ANOVA, Tukey's multiple comparisons test.

 © 2022 The Authors

Figure EV5. Involvement of NIX dimerisation in mitophagy and pexophagy.

A Representative immunoblots of the indicated proteins in lysates of NIX KO ARPE-19 cells (Cl 31) stably expressing a pBabe Flag vector (∅), a pBabe Flag-NIX vector (NIX), a pBabe Flag-NIX G204A vector (G204A), and treated with 1 mM DFP for 48 h prior lysis.

B Representative confocal images of cells as in (A), stably expressing the *mito*-QC reporter or the *pexo*-QC reporter and treated with 1 mM DFP for 48 h (left panel) and flow cytometry analysis of the mCherry/GFP ratio (right panel). Enlarged images of the area outlined in white are shown in the lower corners.

C, D Representative confocal images of NIX KO ARPE-19 cells (Cl 31) stably expressing a pBabe Flag vector (∅), a pBabe Flag-NIX vector (NIX), a pBabe Flag-NIX W36A \L39A vector (W36A\L39A) or a pBabe Flag-NIX G204A vector (G204A), untreated (C) or treated with 1 mM DFP for 48 h (D) and immunostained with a mitochondrial marker (C, ATPB in red) or a peroxisomal marker (D, Catalase in red) and with an anti-NIX antibody (green).

Data information: Nuclei were stained in blue (Hoechst). Scale bars: 10 μm. Data are mean ± s.d.; Statistical significance is displayed as $^{***}P \le 0.001$; $^{****}P \le 0.0001$; ns, not significant. (B) $n = 3$ biological replicates; one-way ANOVA, Tukey's multiple comparisons test.
Source data are available online for this figure.

© 2022 The Authors

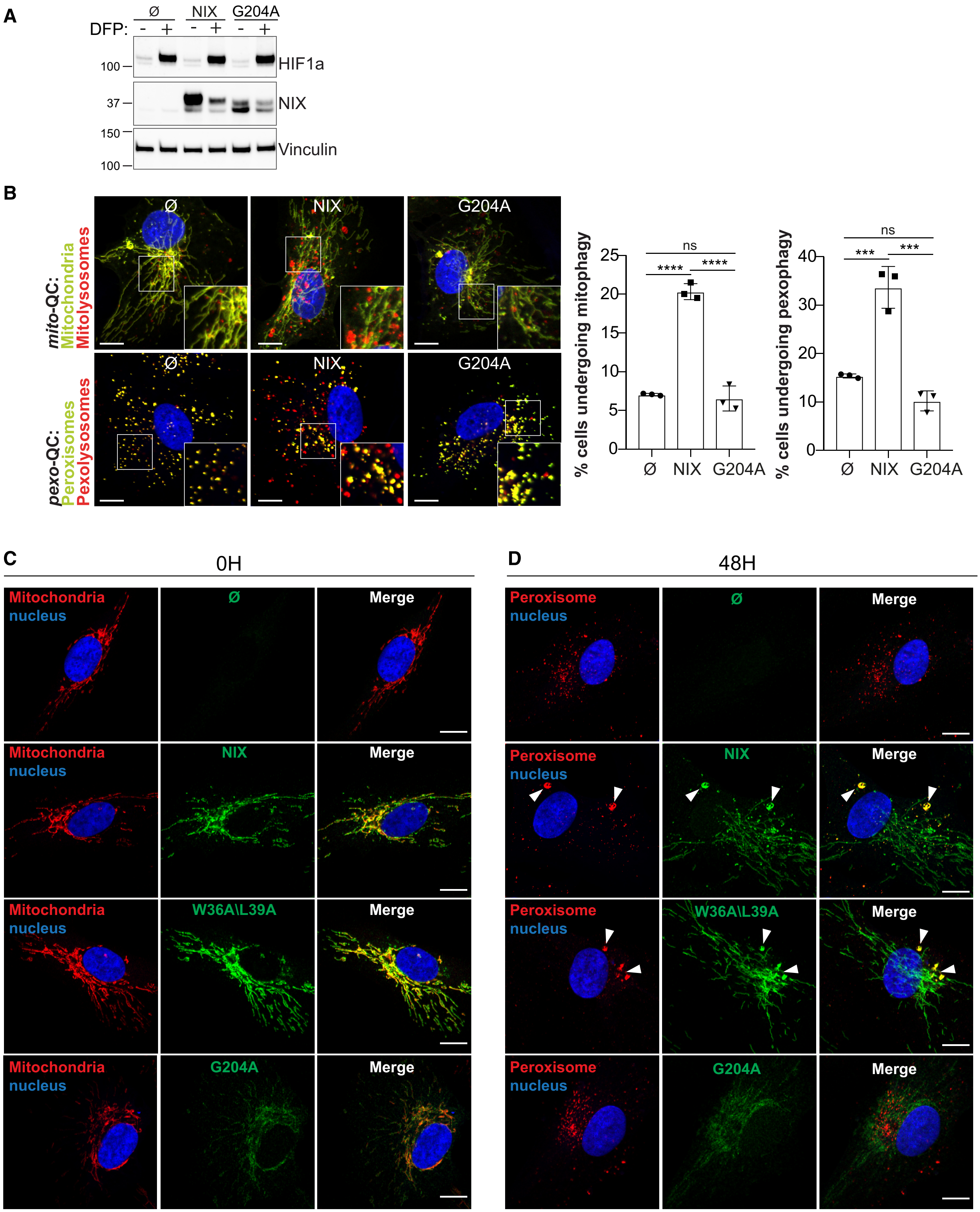

Figure EV5.

 © 2022 The Authors

Figure EV6. Pexophagy occurs during differentiation.

A Representative flow cytometry data of *in vitro* human erythroid cells cultured for 7, 10 and 13 days, as well as of reticulocytes obtained after 20 days in culture. The dot plots show unstained cells (black), cells stained with IgG control antibodies (grey) and cells stained with antibodies against alpha 4 integrin and Band 3 (coloured).

B Representative images of erythroblasts or reticulocytes as in (A), prepared by cytospin and stained with May-Grünwald's stain and Giemsa's stain. Scale bars: 10 μm.

C Immunoblots of the indicated proteins in lysates of erythroblasts or reticulocytes obtained from three different donors.

D Representative immunoblots of the indicated proteins in lysates of *mito*-QC- or *pexo*-QC-H9c2 cells during differentiation.

E Representative confocal images of *pexo*-QC reporter H9c2 cells before (D0) and after 8 days of differentiation (D8). Nuclei were stained in blue (Hoechst). Red-only puncta (pexolysosomes) are depicted with white arrowheads. At right, quantification of total number of pexolysosomes counted per cell analysed during cadiomyocyte differentiation. Overall data are mean ± s.d.; $n = 3$ biological replicates with > 85 cells per condition and replicate, unpaired *t*-test, two-tailed. $^{**}P \leq 0.01$.

F, G Representative confocal images of differentiated *pexo*-QC reporter H9c2 cells immunostained with anti-catalase (F) or anti-LAMP1 (G). White arrowheads indicate structures positive for both red-puncta and LAMP1. Enlarged images of the area outlined in white are shown below.

Data information: Scale bar: 10 μm.
Source data are available online for this figure.

© 2022 The Authors

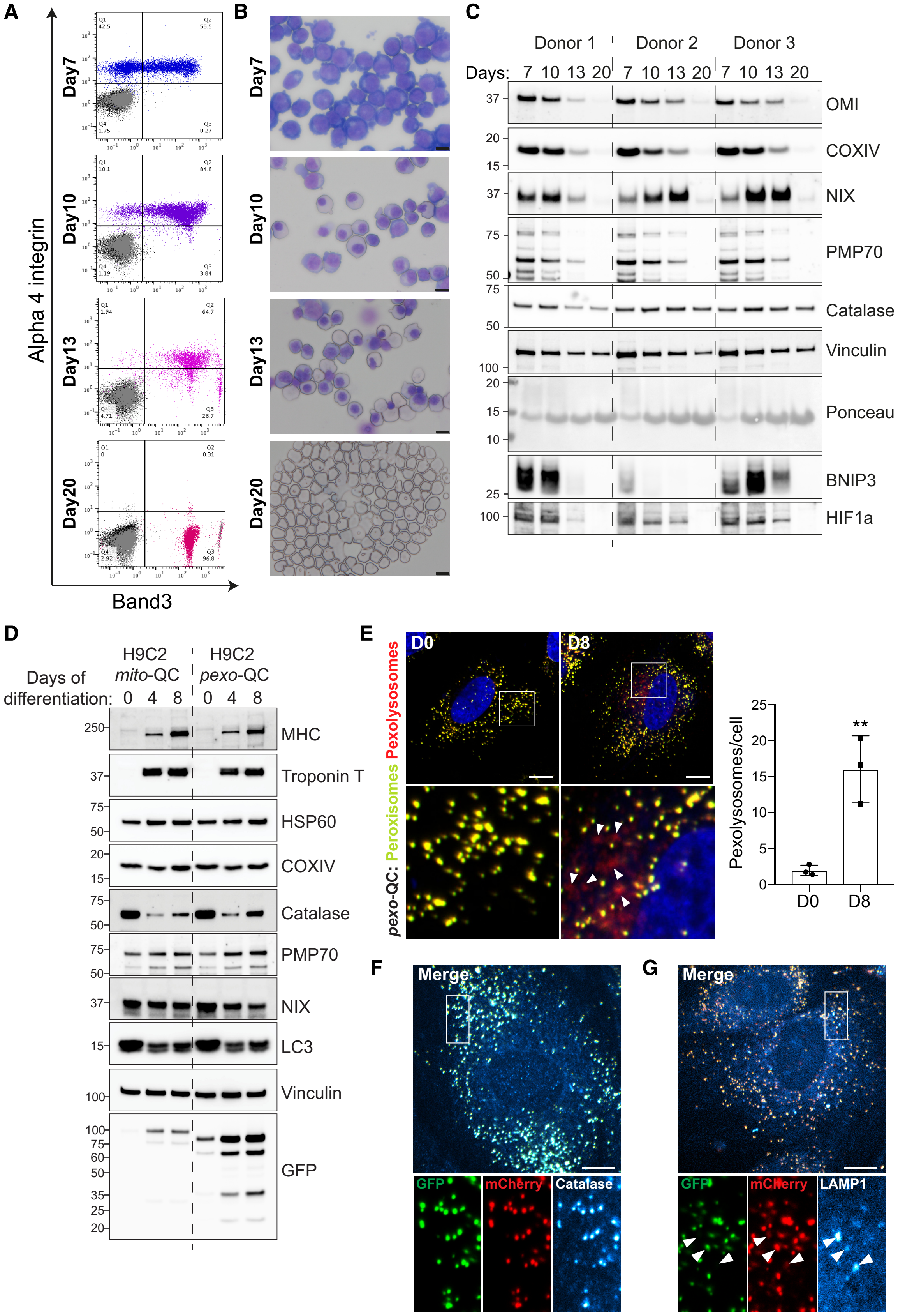

Figure EV6.

 © 2022 The Authors
