# Supplementary material for: BNIP3L/NIX regulates both mitophagy and pexophagy
Source: EMBO J. 2022 Oct 10;41(24):e111115. doi: 10.15252/embj.2022111115 (PMC9753467; doi:10.15252/embj.2022111115)
Supplement: Supplementary file 8 [file EMBJ-41-e111115-s001.pdf]

THE EMBO JOURNAL

BNIP3L/NIX regulates both mitophagy and pexophagy

Léa Wilhelm, Juan Munoz, Beatriz Villarejo-Zori, Stephanie Pellegrin, Catarina Freire, Ashley Toye, Patricia Boya, and Ian Ganley
DOI: 10.15252/embj.2022111115

Corresponding author(s): Ian Ganley (i.ganley@dundee.ac.uk)

Review Timeline:

Submission Date:	9th Mar 22
Editorial Decision:	21st Apr 22
Revision Received:	15th Aug 22
Editorial Decision:	2nd Sep 22
Revision Received:	14th Sep 22
Accepted:	16th Sep 22

Editor: William Teale

Transaction Report:

(Note: With the exception of the correction of typographical or spelling errors that could be a source of ambiguity, letters and reports are not edited. Depending on transfer agreements, referee reports obtained elsewhere may or may not be included in this compilation. Referee reports are anonymous unless the Referee chooses to sign their reports.)

1st Editorial Decision **21st Apr 2022**

Dear Dr Ganley,

Thank you again for the submission of your manuscript entitled "BNIP3L/NIX coordinates mitophagy and pexophagy". I have now received the referees' reports, which are copied to the bottom of this message.

At its heart, all referees agree that the work is based on a technically accomplished and well-described series of experiments. They also state unambiguously that the manuscript is timely and the topic is important. However, the feedback was not unambiguously positive. The data that you present will need to be expanded into a wider biological context if they are to be published in EMBO Journal.

I would like to invite you to address the comments of all referees in a revised version of the manuscript. In particular, I would like to draw your attention to the referee 1's concerns about the localization of endogenous NIX and the need to induce pexophagy in different ways, and referee 2 and 3's points about targeting of the NIX TM domain. The referees ask for a substantial amount of work; if you judge that such experiments are not feasible within a reasonable time-frame, it may be in your best interests to submit the study elsewhere. Our usual revision time of three months is used as a guideline, not a deadline; manuscripts frequently take longer to revise. I will be available and happy to talk next week if you have any questions, I recommend that we go over our next steps and discuss the referees' comments further over Zoom.

I should add that it is The EMBO Journal policy to allow only a single major round of revision and that it is therefore important to resolve these concerns at this stage. I believe the concerns of the referees are reasonable and addressable, but please contact me if you have any questions, need further input on the referee comments or if you anticipate any problems in addressing any of their points. Please, follow the instructions below when preparing your manuscript for resubmission.

I would also like to point out that as a matter of policy, competing manuscripts published during this period will not be taken into consideration in our assessment of the novelty presented by your study ("scooping" protection). We have extended this 'scooping protection policy' beyond the usual 3 month revision timeline to cover the period required for a full revision to address the essential experimental issues. Please contact me if you see a paper with related content published elsewhere to discuss the appropriate course of action.

When preparing your letter of response to the referees' comments, please bear in mind that this will form part of the Review Process File, and will therefore be available online to the community. For more details on our Transparent Editorial Process, please visit our website: https://www.embopress.org/page/journal/14602075/authorguide#transparentprocess

Again, please contact me at any time during revision if you need any help or have further questions.

Thank you very much again for the opportunity to consider your work for publication. I look forward to your revision.

Best regards,

William Teale

William Teale, Ph.D.
Editor
The EMBO Journal

When submitting your revised manuscript, please carefully review the instructions below and include the following items:

1) a .docx formatted version of the manuscript text (including legends for main figures, EV figures and tables). Please make sure that the changes are highlighted to be clearly visible.

2) individual production quality figure files as .eps, .tif, .jpg (one file per figure).

3) a .docx formatted letter INCLUDING the reviewers' reports and your detailed point-by-point response to their comments. As part of the EMBO Press transparent editorial process, the point-by-point response is part of the Review Process File (RPF), which will be published alongside your paper.

4) a complete author checklist, which you can download from our author guidelines (https://wol-prod-cdn.literatumonline.com/pb-assets/embo-site/Author Checklist%20-%20EMBO%20J-1561436015657.xlsx). Please insert information in the checklist that is also reflected in the manuscript. The completed author checklist will also be part of the RPF.

5) Please note that all corresponding authors are required to supply an ORCID ID for their name upon submission of a revised manuscript.

6) We require a 'Data Availability' section after the Materials and Methods. Before submitting your revision, primary datasets produced in this study need to be deposited in an appropriate public database, and the accession numbers and database listed under 'Data Availability'. Please remember to provide a reviewer password if the datasets are not yet public (see https://www.embopress.org/page/journal/14602075/authorguide#datadeposition). If no data deposition in external databases is needed for this paper, please then state in this section: This study includes no data deposited in external repositories. Note that the Data Availability Section is restricted to new primary data that are part of this study.

Note - All links should resolve to a page where the data can be accessed.

7) When assembling figures, please refer to our figure preparation guideline in order to ensure proper formatting and readability in print as well as on screen:
http://bit.ly/EMBOPressFigurePreparationGuideline

Please remember: Digital image enhancement is acceptable practice, as long as it accurately represents the original data and conforms to community standards. If a figure has been subjected to significant electronic manipulation, this must be noted in the figure legend or in the 'Materials and Methods' section. The editors reserve the right to request original versions of figures and the original images that were used to assemble the figure.

8) For data quantification: please specify the name of the statistical test used to generate error bars and P values, the number (n) of independent experiments (specify technical or biological replicates) underlying each data point and the test used to calculate p-values in each figure legend. The figure legends should contain a basic description of n, P and the test applied. Graphs must include a description of the bars and the error bars (s.d., s.e.m.).

9) We would also encourage you to include the source data for figure panels that show essential data. Numerical data can be provided as individual .xls or .csv files (including a tab describing the data). For 'blots' or microscopy, uncropped images should be submitted (using a zip archive or a single pdf per main figure if multiple images need to be supplied for one panel). Additional information on source data and instruction on how to label the files are available at .

10) We replaced Supplementary Information with Expanded View (EV) Figures and Tables that are collapsible/expandable online (see examples in https://www.embopress.org/doi/10.15252/embj.201695874). A maximum of 5 EV Figures can be typeset. EV Figures should be cited as 'Figure EV1, Figure EV2" etc. in the text and their respective legends should be included in the main text after the legends of regular figures.

- For the figures that you do NOT wish to display as Expanded View figures, they should be bundled together with their legends in a single PDF file called *Appendix*, which should start with a short Table of Content. Appendix figures should be referred to in the main text as: "Appendix Figure S1, Appendix Figure S2" etc. See detailed instructions regarding expanded view here: .

- Additional Tables/Datasets should be labelled and referred to as Table EV1, Dataset EV1, etc. Legends have to be provided in a separate tab in case of .xls files. Alternatively, the legend can be supplied as a separate text file (README) and zipped together with the Table/Dataset file.

11) Our journal encourages inclusion of *data citations in the reference list* to directly cite datasets that were re-used and obtained from public databases. Data citations in the article text are distinct from normal bibliographical citations and should directly link to the database records from which the data can be accessed. In the main text, data citations are formatted as follows: "Data ref: Smith et al, 2001" or "Data ref: NCBI Sequence Read Archive PRJNA342805, 2017". In the Reference list, data citations must be labeled with "[DATASET]". A data reference must provide the database name, accession number/identifiers and a resolvable link to the landing page from which the data can be accessed at the end of the reference. Further instructions are available at .

Further instructions for preparing your revised manuscript:

Please make sure you upload a letter of response to the referees' comments together with the revised manuscript.

Please also check that the title and abstract of the manuscript are brief, yet explicit, even to non-specialists.

When assembling figures, please refer to our figure preparation guideline in order to ensure proper formatting and readability in print as well as on screen:
https://bit.ly/EMBOPressFigurePreparationGuideline
See also guidelines for figure legends: https://www.embopress.org/page/journal/14602075/authorguide#figureformat

IMPORTANT: When you send the revision we will require
- a point-by-point response to the referees' comments, with a detailed description of the changes made (as a word file).
- a word file of the manuscript text.
- individual production quality figure files (one file per figure)
- a complete author checklist, which you can download from our author guidelines (https://www.embopress.org/page/journal/14602075/authorguide).
- Expanded View files (replacing Supplementary Information)

Please see out instructions to authors
https://www.embopress.org/page/journal/14602075/authorguide#expandedview

Please remember: Digital image enhancement is acceptable practice, as long as it accurately represents the original data and conforms to community standards. If a figure has been subjected to significant electronic manipulation, this must be noted in the figure legend or in the 'Materials and Methods' section. The editors reserve the right to request original versions of figures and the original images that were used to assemble the figure.

Further information is available in our Guide For Authors: https://www.embopress.org/page/journal/14602075/authorguide

We realize that it is difficult to revise to a specific deadline. In the interest of protecting the conceptual advance provided by the work, we recommend a revision within 3 months (11th Jul 2022). Please discuss the revision progress ahead of this time with the editor if you require more time to complete the revisions. Use the link below to submit your revision:

https://emboj.msubmit.net/cgi-bin/main.plex

Further instructions for preparing your revised manuscript:

Please make sure you upload a letter of response to the referees' comments together with the revised manuscript.

Please also check that the title and abstract of the manuscript are brief, yet explicit, even to non-specialists.

When assembling figures, please refer to our figure preparation guideline in order to ensure proper formatting and readability in print as well as on screen:
https://bit.ly/EMBOPressFigurePreparationGuideline
See also guidelines for figure legends: https://www.embopress.org/page/journal/14602075/authorguide#figureformat

IMPORTANT: When you send the revision we will require
- a point-by-point response to the referees' comments, with a detailed description of the changes made (as a word file).
- a word file of the manuscript text.
- individual production quality figure files (one file per figure)
- a complete author checklist, which you can download from our author guidelines (https://www.embopress.org/page/journal/14602075/authorguide).
- Expanded View files (replacing Supplementary Information)

Please see out instructions to authors
https://www.embopress.org/page/journal/14602075/authorguide#expandedview

Please remember: Digital image enhancement is acceptable practice, as long as it accurately represents the original data and conforms to community standards. If a figure has been subjected to significant electronic manipulation, this must be noted in the figure legend or in the 'Materials and Methods' section. The editors reserve the right to request original versions of figures and the original images that were used to assemble the figure.

Further information is available in our Guide For Authors: https://www.embopress.org/page/journal/14602075/authorguide

We realize that it is difficult to revise to a specific deadline. In the interest of protecting the conceptual advance provided by the work, we recommend a revision within 3 months (20th Jul 2022). Please discuss the revision progress ahead of this time with the editor if you require more time to complete the revisions. Use the link below to submit your revision:

https://emboj.msubmit.net/cgi-bin/main.plex

Referee #1:

In a manuscript by Wilhelm et al., the authors analyze the role of autophagy adaptor BNIP3L/Nix in coordinated mitophagy and pexophagy. Despite the metabolic functions of peroxisomes and mitochondria and some common proteins localizing to both organelles, the coordination of these organelles, including their turnover under changing metabolic conditions, is not well understood. The authors use the iron chelator deferiprone (DFP) model and find that mitophagy and pexophagy induced under these conditions require the activity of BNIP3L/Nix and can occur independently of PINK1/Parkin pathway. They also found that BNIP3L localizes to peroxisomes, in addition to known mitochondrial localization of this protein. Overall, the high technical quality data from well-controlled experiments support the conclusion that BNIP3L is required for iron depletion-induced pexophagy. The primary issue with this work is that it focuses on DFP-treatment and does not analyze pexophagy under somewhat more physiologically relevant conditions. For example, does depletion of BNIP3L inhibits starvation-induced pexophagy? The data showing different levels of peroxisomal markers in Nix KO retina only partially support the pexophagy angle. Another weakness is that the peroxisomal localization of NIX is only detected using exogenous GFP-tagged NIX. Given that overexpressed proteins can be targeted to many cellular locations nonspecifically, the authors should address this issue with new experiments. At the minimum, cell fractionation and Western blot would address this issue (assuming that suitable antibodies for immunofluorescence or immune EM are not available).

Specific comments:
1. The authors proposed that treatment with DFP mimics hypoxic conditions. Therefore, it would be interesting to test whether a similar mechanism of pexophagy also occurs under hypoxia.
2. While the mito-QC and pexo-QC tools are quite revealing, it would be important to present data indicating mitochondrial and peroxisomal colocalization of LC3 as an independent method verifying pexophagy (and mitophagy) in DFP-treated WT and NIX KO cells.
3. Degradation of p62 (Figure 1A), which primary role is in Ub-mediated autophagy, suggest that ubiquitination may occur in the DFP model. Another possibility is that p62 is degraded by autophagy nonspecifically. Does p62 play a role in DFP-induced pexophagy? Looking at p62 localization in relation to peroxisomes in DFP-treated cells could partially answer this question.
4. Considering predominantly mitochondrial localization of Nix, it is surprising that NIX protein levels were dramatically increased by DFP in CCCP-treated Parkin overexpressing cells, but not in DFP "-" CCCP-treated cells (Fig. 5C). The authors conclude that these cells are deficient in mitochondria. Does DFP-specifically induce NIX in these cells without re-formation of the mitochondria?

Referee #2:

In this manuscript, Wilhelm et al. report the role of BNIP3L/NIX, a mammalian protein that has been suggested to promote mitochondria-specific autophagy (mitophagy), in autophagy-dependent degradation selective for peroxisomes (pexophagy). Mitophagy and pexophagy are the major selective autophagy-related processes whose basic principles are conserved among eukaryotes: ubiquitin chains conjugated to proteins on the organellar surface, or LIR (LC3-interacting region)/AIM (Atg8-family interacting motif)-containing specific proteins anchored to, or tightly associated with, the surface of organelles, establish the specificity and activity of their clearance. In mammals, multiple molecular mechanisms underlying mitophagy have been uncovered, whereas proteins promoting pexophagy remain poorly understood. In this study, the authors demonstrate that iron-depleted mammalian cultured cells undergo both mitophagy and pexophagy in a manner dependent on NIX, a LIR-containing single-pass membrane protein that has been shown to promote mitophagy during reticulocyte maturation and under several stress conditions including hypoxia. When treated with the iron chelator deferiprone (DFP), cells upregulated the hypoxia-inducible transcription factor HIF1a and its downstream target NIX. The authors also found that DFP-induced mitophagy and pexophagy require the core autophagy-related proteins ULK1 and ATG13, indicating that these catabolic pathways rely on the conventional autophagic machinery. As expected, loss of NIX suppresses degradation of mitochondrial and peroxisomal proteins, and transport of mitophagy- and pexophagy-specific probes to the lysosome. Remarkably, DFP treatment induced localization of NIX to peroxisomes in a manner dependent on its transmembrane domain, raising the possibility that a fraction of NIX is anchored to the surface of peroxisomes and promotes pexophagy. This iron chelation-induced NIX localization to peroxisomes were seen even in cells devoid of mitochondria. Finally, the authors focused on peroxisomal clearance in a physiological context and showed a NIX-dependent decrease of peroxisomal proteins in retina, and an increase in transport of peroxisomes to lysosomes during cardiomyocyte differentiation.

The findings in this study are potentially interesting and could provide new insights into the molecular mechanisms of pexophagy in mammals. However, there are several uncertain points that do not fit with the others in this manuscript, and missing data that are needed to fully support the model for NIX-mediated pexophagy. It also remains unclear how targeting of NIX to peroxisomes is inducibly regulated under iron depletion conditions. In conclusion, this study would significantly be strengthened if the authors clarify these issues and address the following points.

Specific points:

1. Upon DFP treatment, HIF1a is upregulated, which in turn induces NIX expression to promote mitophagy and pexophagy (Figure 3A). The authors should try to test whether cells expressing constitutively stable HIF1a mutant (P402A/P564A) (Zhao et al., Cell Stress 4: 99-113 [2020]) can trigger NIX induction and facilitate mitophagy and pexophagy independently of iron depletion.

2. As described in Figure 3C, the authors should examine molecular functions of several NIX domains such as LIR, BH3 (BCL2 homologue 3), several phosphorylation sites, and homodimerization motifs for pexophagy.

3. In Figure EV3A, overexpression of NIX in NIX KO cells did not lead to reduction of proteins in mitochondria and peroxisomes, even though the protein levels were very similar to those in DFP-treated WT cells. Moreover, when these NIX-overexpressing cells were treated with DFP, the NIX levels were strongly decreased, which is inconsistent with the idea that NIX induction is critical to promote mitophagy and pexophagy. The authors should reconcile this discrepancy.

4. It is conceivable that the NIX transmembrane (TM) domain serves as a signal for dual targeting/insertion to mitochondria and peroxisomes (Figures 4C and EV4C). The authors should investigate how NIX targets to peroxisomes in more depth. For example, can GFP-NIX (TM-only) localize to both mitochondria and peroxisomes? If so, does the peroxisomal targeting occur only in response to iron depletion?

5. In Figure 5C, NIX is induced in cells and cells overexpressing Parkin even without iron depletion, which does not, however, promote degradation of mitochondrial and peroxisomal proteins. Does it mean that NIX induction by itself is not sufficient for mitophagy and pexophagy? The authors should perform fluorescence microscopy to localize NIX in these cells and discuss about their observations.

Referee #3:

In this manuscript, Ganley and colleagues describe a novel function of NIX in pexophagy induced by iron-chelation. First, they show that DFP treatment induces ferritinophagy, mitophagy and pexophagy sequentially. NIX is not required for ferrtinophagy, but it is essential for the clearance of the two organelles. NIX KO cells exhibit a significant block in both mitophagy and pexophagy. To assess how a mitochondrial protein (NIX) may be involved in pexophagy, the authors found, surprisingly, that NIX translocates to peroxisomes after DFP treatment. This translocation is not dependent on mitochondria. Finally, they show that NIX regulates peroxisomal levels in physiological contexts such as retina development and erythrocyte maturation as well as cardiomyocyte differentiation.

The unexpected role of NIX in pexophagy and its translocation to peroxisomes are intriguing and could lead to new directions regarding how organelle clearance can be connected. The data are pretty solid and experiments are well designed. However, the authors should dig a little bit deeper in terms of the mechanisms and some conclusions still need more evidence to be convincing.

Major points:

1. BNIP3 is a close homolog of NIX and both have been shown to be involved in the same mitophagy pathway. While the role of NIX in physiological processes such as reticulocyte maturation is well known, BNIP3 has not been shown to be required for the same process. Is it because BNIP3/NIX are differentially regulated or may have distinct functions? Therefore, it will be very informative for the authors to determine if BNIP3 is also is involved in pexophagy, and if it translocates to peroxisomes. Such information will reveal if NIX uniquely evolved for pexophagy.

2. The authors fail to explore how NIX translocates to peroxisomes after DFP treatment. This is a very important question and a couple of simply experiments will provide some clues. For instance, will artificially targeting NIX to peroxisomes by removal of the TM of NIX and replacing it with peroxisome targeting TM such as that of PEX26 used previously (https://doi.org/10.1083/jcb.201605002) induce pexophagy? Vice versa, please replace the TM of NIX with that of OMP25 to see if that prevents NIX translocation to peroxisomes and pexophagy.

3. Pexophagy and interfering of peroxisome biogenesis lead to the same phenotype: less of peroxisomes. One way to distinguish the two processes is to see if the peroxisomal loss can be reversed by blocking the general autophagy machinery. Therefore, while it is nice for the authors to show the block of pexophagy in NIX KO mice retina, do they see the same blocking phenotype in FIP200 KO mice? Unfortunately, most of the ATG KO mice are lethal. But the FIP200 KI/KI (knocking of a LQFL/AAAA mutant) is viable. It's a big to ask for such in a revision, but at least it should be considered for future work and discussed in the discussion.

4. NIX translocation to peroxisomes seems not very robust based in the IF images and only a fraction of peroxisomes show NIX

localization. To make it much more convincing, the authors should check NIX translocation in Drp1 KO or KD cells, where peroxisomes will become very elongated and filament-like. Will Bafilomycin treatment (blocking pexophagy) enhance NIX translocation? Will hypoxia also cause NIX translocation? Since HIF1 is required for NIX up-regulation, it might also be required for pexophagy. The authors should check this.

5. In all the WB they performed to show pexophagy, only PMP70 and catalase levels are examined. Some additional peroxisomal markers such as PEX14, PEX2, PEX10, PEX13 or other matrix proteins such as alcohol oxidase (AOx) and 3-ketoacyl-CoA thiolase (ACAA2) should be examined.

6. The title says "coordinates" which is not really supported by the data. They only show NIX is required for both mitophagy and pexophagy, but coordination of the two processes is not shown. They also state that mitophagy and pexophagy occur sequentially, which is not really clear. Once NIX is unregulated by HIF1, it may initiate the two processes simultaneously. The reason the authors saw slower clearance or degradation of peroxisomes than mitochondria may simply be because there is much less NIX translocated to peroxisomes.

Minor comments

Fig. 1B. In addition to the count of the number of pexolysosomes/cell, the number of peroxisomes/cell should be counted as well to determine if some of the peroxisomes are completely cleared.
Fig. 1D. It's odd that LC3 expression is dramatically increased in both siATG13 and siULK1 even without DFP treatment. Also, LC3 lipidation seems to be completely blocked in siATG13 but not siULK1. Can the authors explain?

Fig. 2B. While pS65 Ub is not detected in DFP or Ivermectin treated cells, it can't be ruled out that other types of ubiquitination could be involved. Indeed, Sulkshane et al in a recent paper (Redox Biology, 2021, https://doi.org/10.1016/j.redox.2021.102047) showed that increased ubiquitin levels was detected on mitochondria after CoCl2 treatment or under hypoxic conditions. So, ideally, subcellular fractionation should be checked to see if more ubiquitin can be detected on mitochondria after DFP treatment.

Fig. 2C. It's odd that FK2 staining detected some puncta in the control but not in DFP samples. The IF should be repeated with co-staining of Tom20 to see if those puncta are on mitochondria.

Fig. 5C. It's surprising that NIX levels are similar between untreated and DFP treated cells. Isn't NIX supposed to be induced by DFP treatment (compare to Fig. EV3A)? Why is NIX not degraded like other mitochondrial proteins after 48h treatment? Is it because NIX is constantly induced? In this case, NIX should not be on mitochondria anymore, right?

Fig. 6A. While PMP70 staining shows the normal peroxisomal localization (spots), catalase staining does not show the pattern like PMP70, more like diffuse and cytosolic. Why? Mitochondrial marker should be also checked to verify the mitophagy is blocked in NIX KO retina.

Fig. EV1F. The FACS plots show that the GFP/mCherry ratio is already quite different in each cell samples without treatment, indicating the basal pexophagy level is different. It should be adjusted to 1% individually so that the effect of DFP over control can be properly measured.

Fig. EV3A. Why is the exogenous NIX (from pBabe-NIX) significantly reduced in the rescued cells (KO + pBabe-NIX) but endogenous NIX is not degraded (WT)?

Fig. EV6D. It is understandable that GFP-NIX is degraded with DFP treatment. But why is GFP-NiXdeltaTM also degraded with DFP? This truncated NIX would be cytosolic and shouldn't be degraded by mtiophagy or pexophagy.

Fig. EV6C-6D. In 6C, PMP70 degradation is significant but not catalase. But in 6D, it's the opposite - catalase degradation is very obvious but not PMP70. Why? NIX levels seems to be very high from Day 0 of differentiation and don't change much on Day 4 and Day 8. Can the authors explain this? In both 6C-6D, HIF1 levels should be checked as well as BNIP3. It's mysterious that NIX but not BNIP3 is required for mitochondria clearance in reticulocytes. Therefore, it will be very informative to show if HIF1 induction of BNIP3 is not present in these situation.

Page 12. Please explain Band3 and alpha-4-integrin a little bit so readers can understand why you checked these two markers.

Firstly, we would like to thank the Editor and Reviewers for all their hard work in assessing our manuscript and providing helpful and constructive suggestions. We have tried to address these – hopefully to the satisfaction of everybody. The manuscript is indeed much improved.

Editor Comments:
I would like to invite you to address the comments of all referees in a revised version of the manuscript. In particular, I would like to draw your attention to the referee 1's concerns about the localization of endogenous NIX and the need to induce pexophagy in different ways, and referee 2 and 3's points about targeting of the NIX TM domain.

In the original manuscript, we had significant endogenous co-localisation between NIX and peroxisomal markers upon DFP treatment. However, we apologise if this was not made clear and perhaps the assumption was that it was only GFP-NIX colocalising (given this figure was first). We have tried to make this more obvious in the text at multiple points. Example image of endogenous NIX is shown below (from new Fig 4, but also see new EV3):

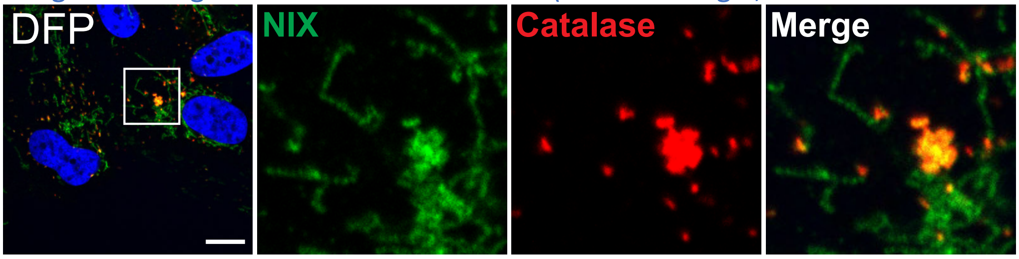

From Fig. 4D

As detailed below, we have now induced pexophagy with hypoxia, in addition to DFP, and we have made multiple mutants of NIX to gain more mechanistic insight into its role during pexophagy. The manuscript contains numerous new experimental data, which has resulted in significant rearrangement of the figures to accommodate these. To aid in the review, we have included a version of the manuscript with the major text changes highlighted in blue.

Referee #1:
Specific comments:

1. The authors proposed that treatment with DFP mimics hypoxic conditions. Therefore, it would be interesting to test whether a similar mechanism of pexophagy also occurs under hypoxia.

We thank the Reviewer for this suggestion for a more physiological stimulus. We have now found that hypoxia does indeed induce NIX expression and pexophagy. These data are now included in new Fig. EV1D-E and discussed on page 5 of the new manuscript:

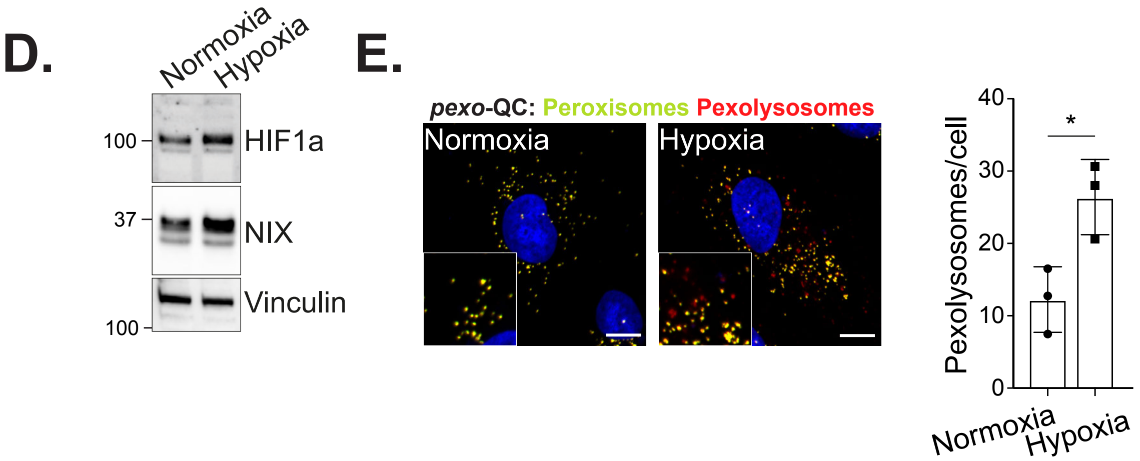

Relatedly, we show that HIF1a is essential for pexophagy (new Fig. 2A-B). In addition, we also show that both GFP-NIX or endogenous NIX localises to peroxisomes upon hypoxia treatment. These data are shown in new Fig. EV3C and discussed on page 10:

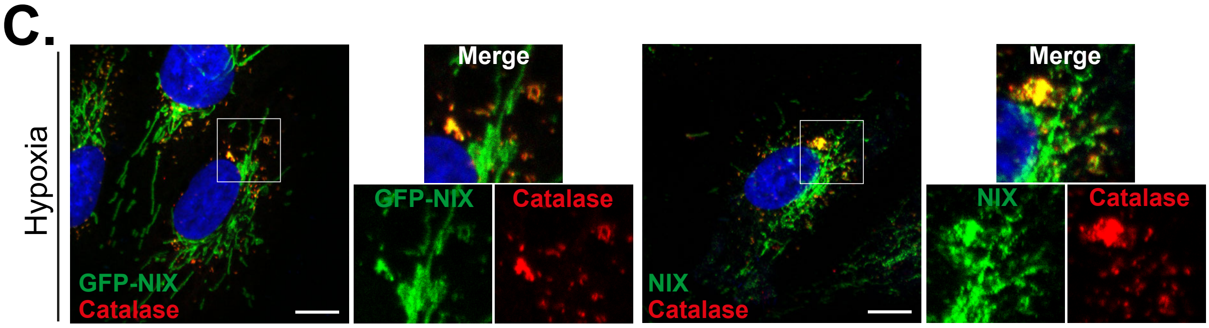

2. While the mito-QC and pexo-QC tools are quite revealing, it would be important to present data indicating mitochondrial and peroxisomal colocalization of LC3 as an independent method verifying pexophagy (and mitophagy) in DFP-treated WT and NIX KO cells.

We note that we did use a mito/pexo-QC reporter-independent method to study NIX mediated mitophagy and pexophagy (western blot data showing loss of protein is blocked upon NIX KO – Previous Fig. 3 but now shown in new Fig. 2C and F). We also showed that LC3 colocalises with peroxisomes (old Fig. 5D, now new Fig. 5A). However, we appreciate the comment, which was also related to one raised by Referee 2. To strengthen the role for LC3/ATG8s in NIX-dependent pexophagy, we rescued NIX KO cells with WT and LIR mutant NIX – we found that both mitophagy and pexophagy were significantly impaired with LIR mutant compared to WT. These data are shown in new Fig. 5B-D and discussed on page 12:

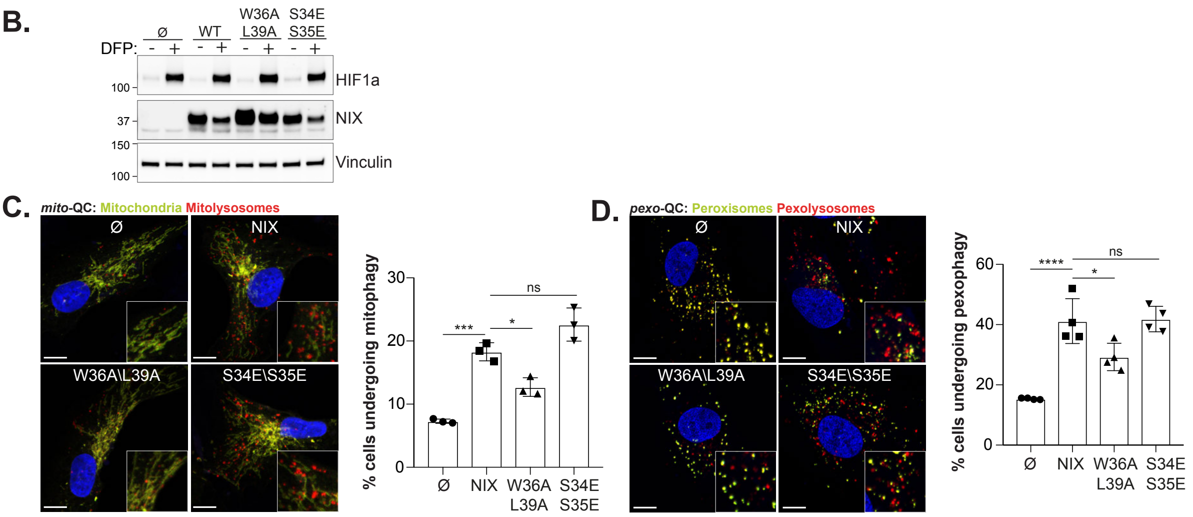

3. Degradation of p62 (Figure 1A), which primary role is in Ub-mediated autophagy, suggest that ubiquitination may occur in the DFP model. Another possibility is that p62 is degraded by autophagy nonspecifically. Does p62 play a role in DFP-induced pexophagy? Looking at p62 localization in relation to peroxisomes in DFP-treated cells could partially answer this question.

This is an interesting point. We carried out the suggested experiment and found no significant co-localisation of p62 with peroxisomes (catalase) upon DFP treatment. This is in contrast to EBSS starvation that has previously been shown to c ause p62/NBR1 – peroxisome colocalization. We thus conclude that p62 is being turned over independently. The new data is shown in Fig. EV5D and discussed on page 14:

D.

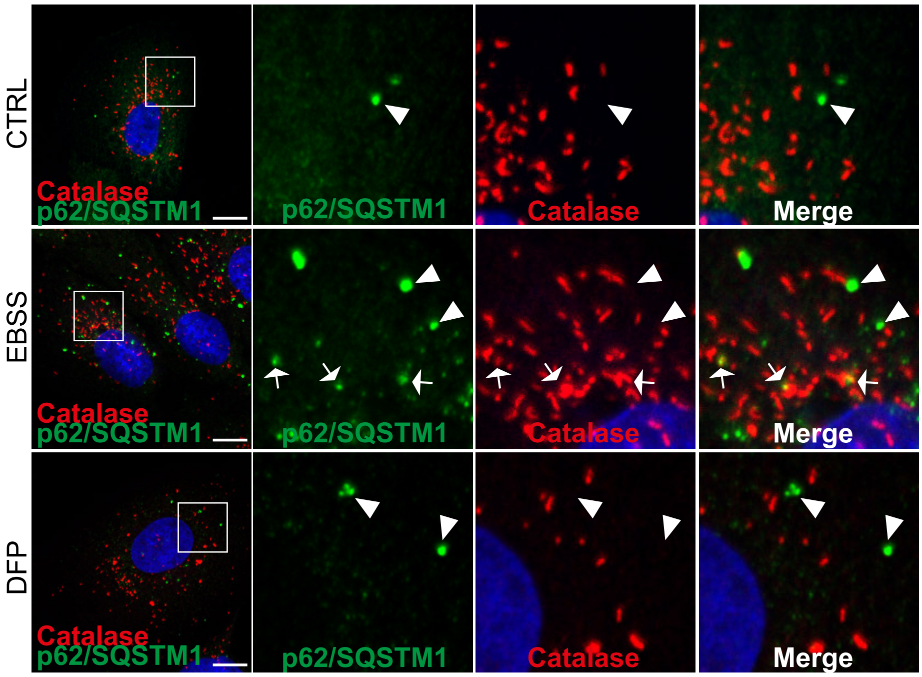

4. Considering predominantly mitochondrial localization of Nix, it is surprising that NIX protein levels were dramatically increased by DFP in CCCP-treated Parkin overexpressing cells, but not in DFP "-" CCCP-treated cells (Fig. 5C). The authors conclude that these cells are deficient in mitochondria. Does DFP-specifically induce NIX in these cells without re-

formation of the mitochondria?

We apologise as this was perhaps confusing in the text. Yes, we believe the Reviewer is correct - in the absence of mitochondria, DFP still stabilises HIF1a, which in turn transcriptionally upregulates NIX. As there are no mitochondria, this localises to peroxisomes. We have now included HIF1a in a revised blot to show DFP still results in its stabilisation in the absence of mitochondria, as well as schematic of the process to aid readers (new Fig. 4A-B and mentioned in text on page 11):

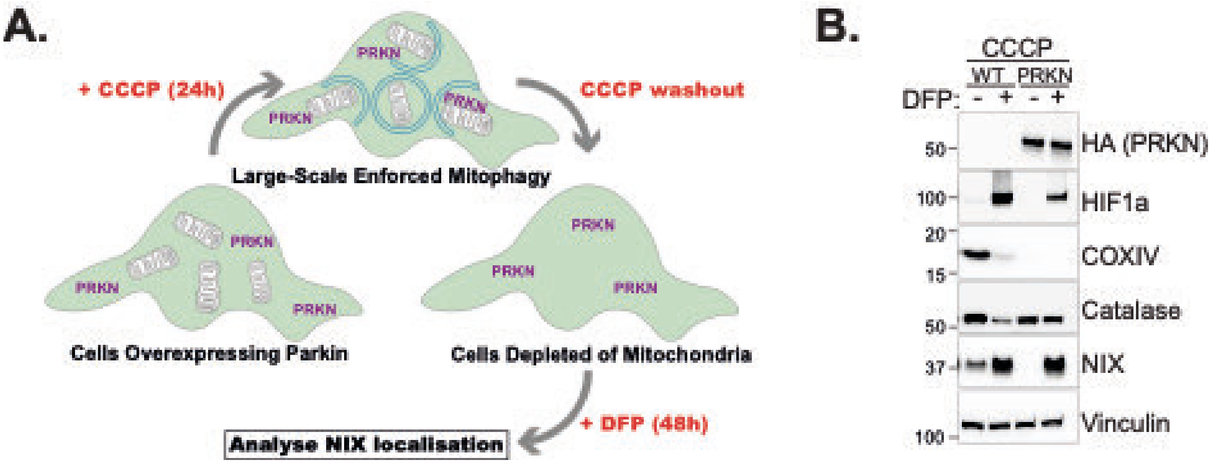

Referee #2:

Specific points:

1. Upon DFP treatment, HIF1a is upregulated, which in turn induces NIX expression to promote mitophagy and pexophagy (Figure 3A). The authors should try to test whether cells expressing constitutively stable HIF1a mutant (P402A/P564A) (Zhao et al., Cell Stress 4: 99-113 [2020]) can trigger NIX induction and facilitate mitophagy and pexophagy independently of iron depletion.

We thank the Reviewer for this suggestion, which was also related to point 1 raised by Reviewer 1. To address both points more easily, we decided to use a previously generated HIF1a KO cell line (Zhao et al 2020) to confirm a role for HIF1 in pexophagy. These new data are shown new Fig. 2A-B and is described on page 7 of the new manuscript:

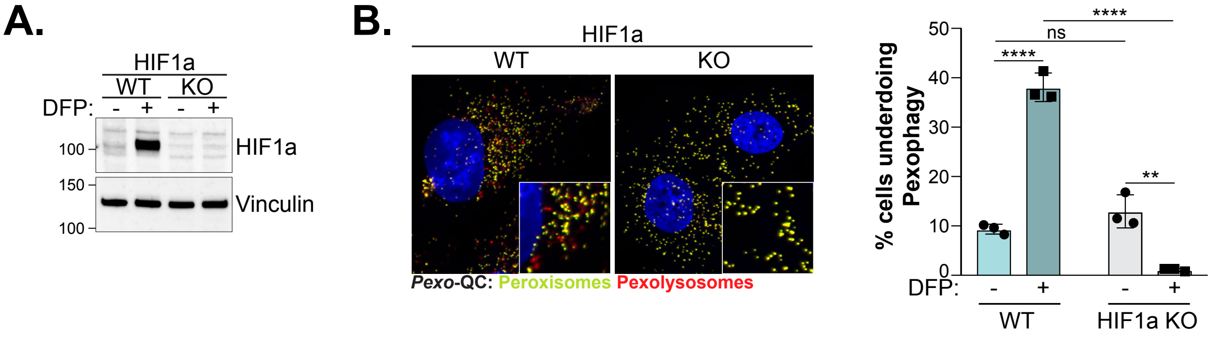

2. As described in Figure 3C, the authors should examine molecular functions of several NIX domains such as LIR, BH3 (BCL2 homologue 3), several phosphorylation sites, and homodimerization motifs for pexophagy.

We thank the Reviewer for these more mechanistic suggestions that have generated a lot of useful information. We made several NIX mutants and used these in “NIX-KO rescue” experiments. We made the LIR mutant (W36A/L39A) and found that its ability to rescue mitophagy and pexophagy was significantly less than that of WT (our mitophagy data is very similar to that previously published in the original Novak et al 2010 paper). We also made the phosphomimetic mutant (S34E/S35E) and found this rescued levels to a similar amount as WT. As stated in the text, it is not known how these residues are phosphorylated – so it is possible that baseline phosphorylation is already high in this cell type (hence no significant increase in mitophagy/pexophagy. These data are shown in new Fig. 5B-D and described on page 12:

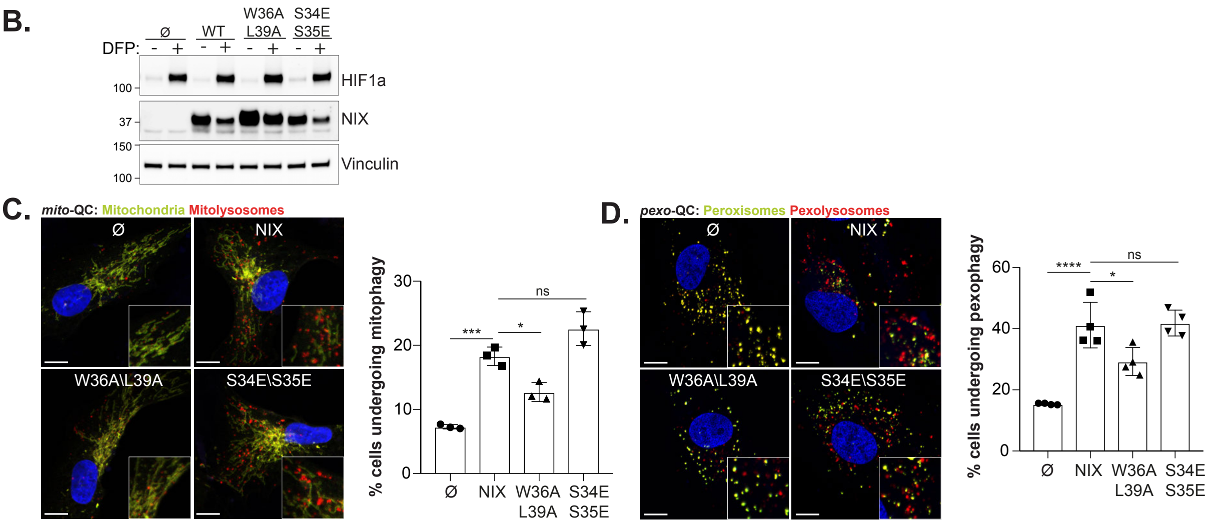

We also generated the NIX dimer mutant (G204A) and as with the previously published work, we found this was unable to rescue mitophagy in NIX KO cells. Importantly though, this was also unable to rescue pexophagy. These new data are shown in Fig. EV5 E-F and described on page 13:

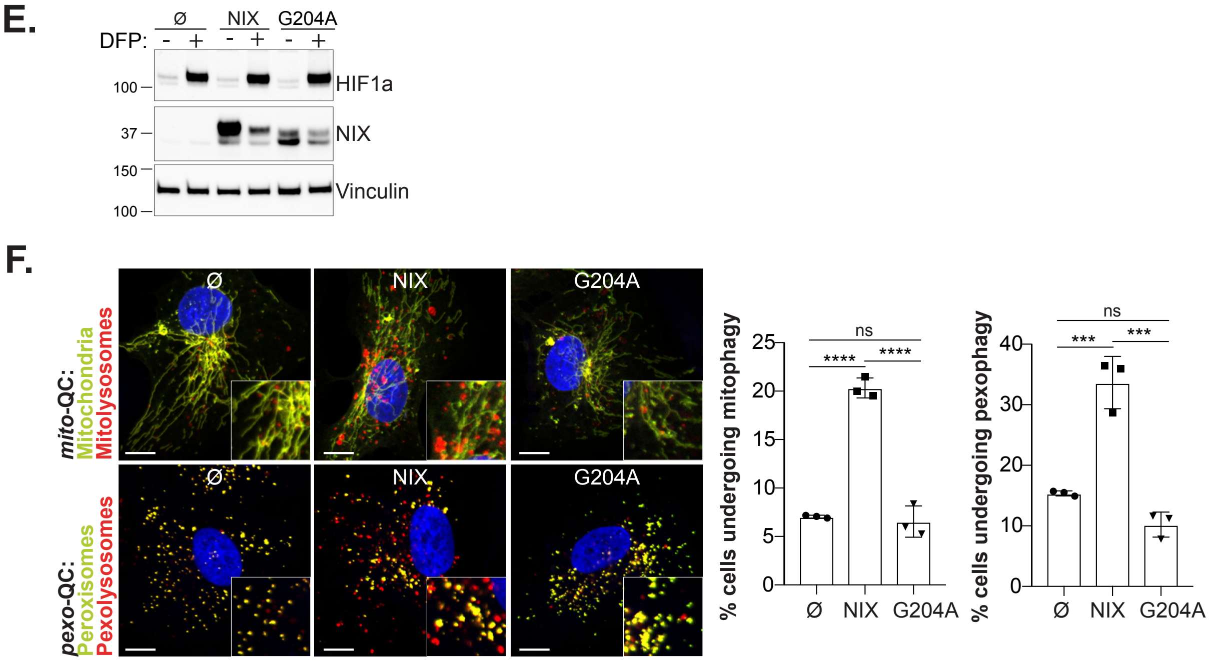

3. In Figure EV3A, overexpression of NIX in NIX KO cells did not lead to reduction of proteins in mitochondria and peroxisomes, even though the protein levels were very similar to those in DFP-treated WT cells. Moreover, when these NIX-overexpressing cells were treated with DFP, the NIX levels were strongly decreased, which is inconsistent with the idea that NIX induction is critical to promote mitophagy and pexophagy. The authors should reconcile this discrepancy.

We apologise as perhaps this was not well explained in the original text. Firstly, though NIX expression is critical for mitophagy/pexophagy it is not sufficient on its own (hence overexpression of NIX without DFP does not stimulate large-scale loss of mito/pexo proteins). Secondly, this overexpressed NIX is not under the endogenous promoter, so it is not transcriptionally upregulated with DFP (as with endogenous). In this scenario, its rate of degradation exceeds its rate of transcription and therefore total levels decrease. The old Fig. EV3A is now the new Fig. 2F and we have included the following text on page 8 to hopefully clarify this:

"We found that overexpression of NIX alone in the KO cells, in the absence of DFP, was insufficient to restore mitophagy and pexophagy even though levels were comparable to WT cells treated with DFP. This suggests that although NIX is essential for mitophagy and pexophagy, it is not sufficient for a robust response by itself in these cells. We are currently exploring what these additional HIF1-dependent inputs might be. It is also important to note that the endogenous and exogenous protein levels of NIX differ in response to DFP. This is likely because endogenous NIX has the HIF1-responsive elements within its promoter and is strongly induced upon DFP treatment, to a level that exceeds the rate of mitophagy/pexophagy, hence it accumulates. In contrast, exogenous NIX has no such promoter elements and is expressed constitutively at a rate that does not exceed that of its turnover via mitophagy/pexophagy, hence its levels decrease with DFP treatment."

4. It is conceivable that the NIX transmembrane (TM) domain serves as a signal for dual targeting/insertion to mitochondria and peroxisomes (Figures 4C and EV4C). The authors should investigate how NIX targets to peroxisomes in more depth. For example, can GFP-NIX (TM-only) localize to both mitochondria and peroxisomes? If so, does the peroxisomal targeting occur only in response to iron depletion?

This was a good suggestion and so we made the GFP-NIX-TM only construct. The TM only domain was sufficient to localise to mitochondria – see new Fig. EV3E and discussion on page 10:

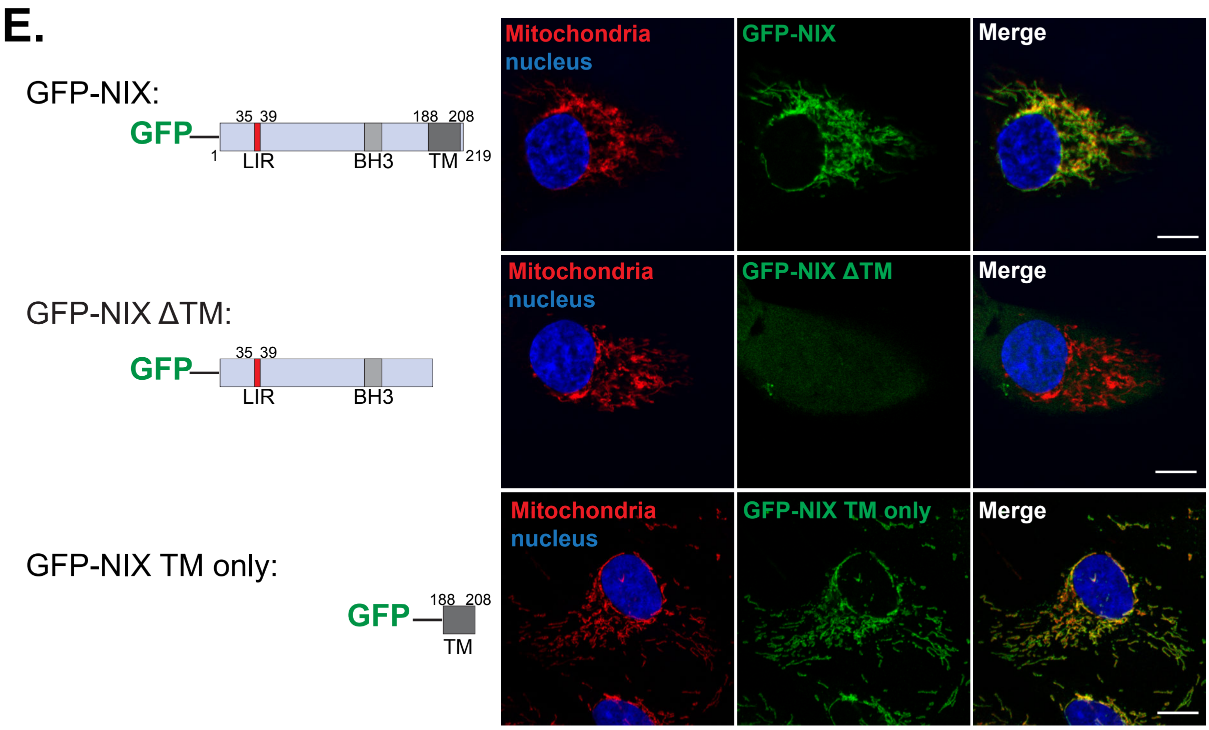

Additionally, the TM only domain localised to peroxisomes upon DFP treatment – see new Fig. 3C:

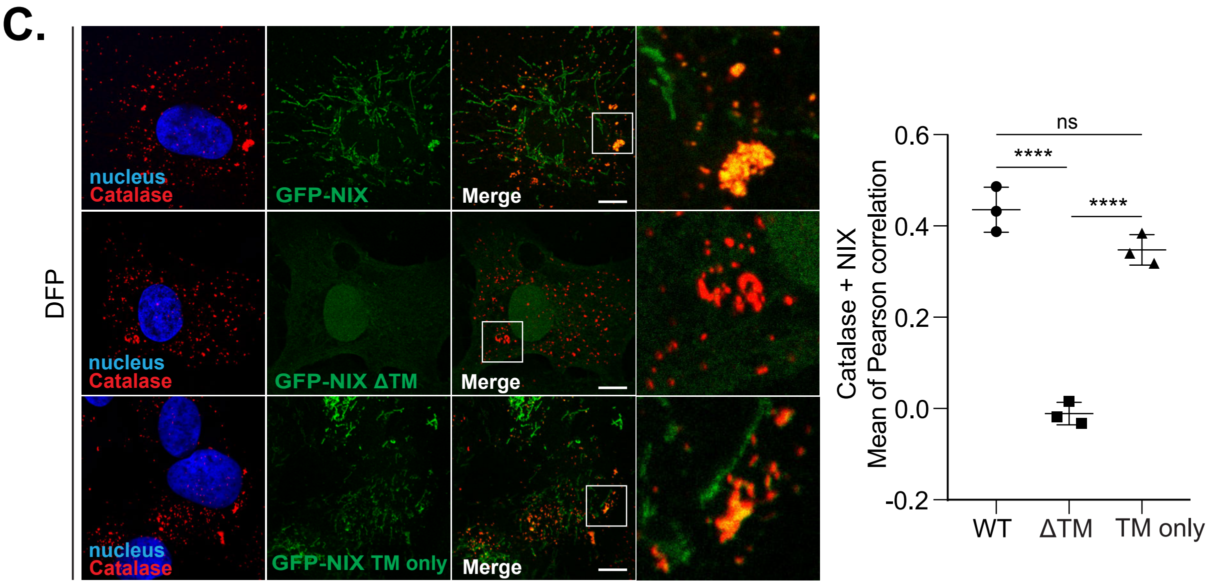

5. In Figure 5C, NIX is induced in cells and cells overexpressing Parkin even without iron depletion, which does not, however, promote degradation of mitochondrial and peroxisomal proteins. Does it mean that NIX induction by itself is not sufficient for mitophagy and pexophagy? The authors should perform fluorescence microscopy to localize NIX in these cells and discuss about their observations.

Apologies, as before perhaps this was not explained well. As mentioned in point 3, NIX is necessary but not sufficient for mitophagy and pexophagy. Referee 3 also questioned these blots in terms of NIX expression, and we do think that NIX is further induced upon iron depletion. However, we cannot yet fully explain why we do not see a similar strong induction of NIX in the non-CCCP cells in these experiments (as is seen in the other

experiments). But, the time course in this experiment is much longer (24h -/+ CCCP, then 24h recovery, then 48h DFP) and the non-CCCP cells are significantly more confluent compared to the CCCP-treated ones. We have previously noted that induced NIX levels can be lower in confluent cells and here, we think that although NIX is induced its level is not quite as high and is balanced by its autophagic turnover (hence no large accumulation). However, as the non-CCCP treated cells are not used in our colocalization experiments or discussed in the text (and the best control is already present (WT vs Parkin overexpression)), we have cropped the blot showing the non-CCCP treated samples to avoid confusion (new Fig. 4B).

Referee #3:
Major points:

1. BNIP3 is a close homolog of NIX and both have been shown to be involved in the same mitophagy pathway. While the role of NIX in physiological processes such as reticulocyte maturation is well known, BNIP3 has not been shown to be required for the same process. Is it because BNIP3/NIX are differentially regulated or may have distinct functions? Therefore, it will be very informative for the authors to determine if BNIP3 is also is involved in pexophagy, and if it translocates to peroxisomes. Such information will reveal if NIX uniquely evolved for pexophagy.
We thank the Reviewer for this suggestion and we explored a role for BNIP3 in pexophagy. As we have previously found with mitophagy (Zhao et al., 2020), it appears that BNIP3 can also influence pexophagy in ARPE19 cells. Using siRNA of NIX and BNIP3, alone or in combination, we found a degree of redundancy with respect to pexophagy. These data are shown in new Fig. EV2 and discussed on page 9:

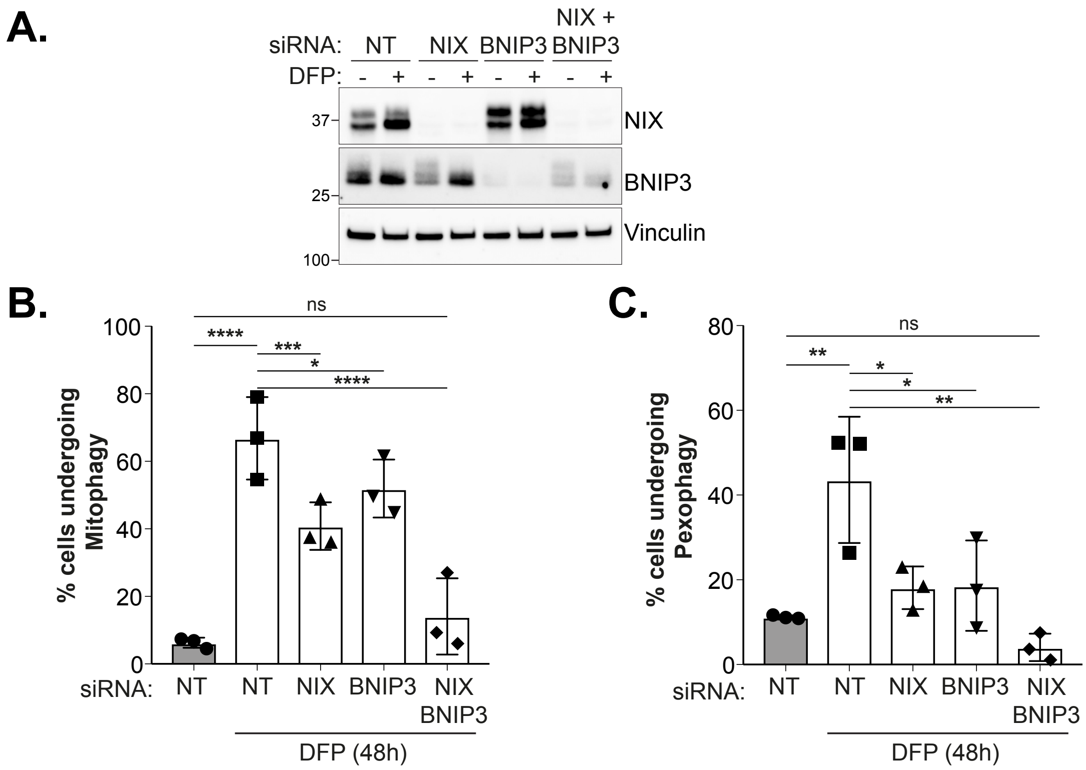

We also show that GFP-BNIP3 can localise to peroxisomes upon DFP treatment (similar to NIX). This is shown in new Fig. EV3D and mentioned on page 10:

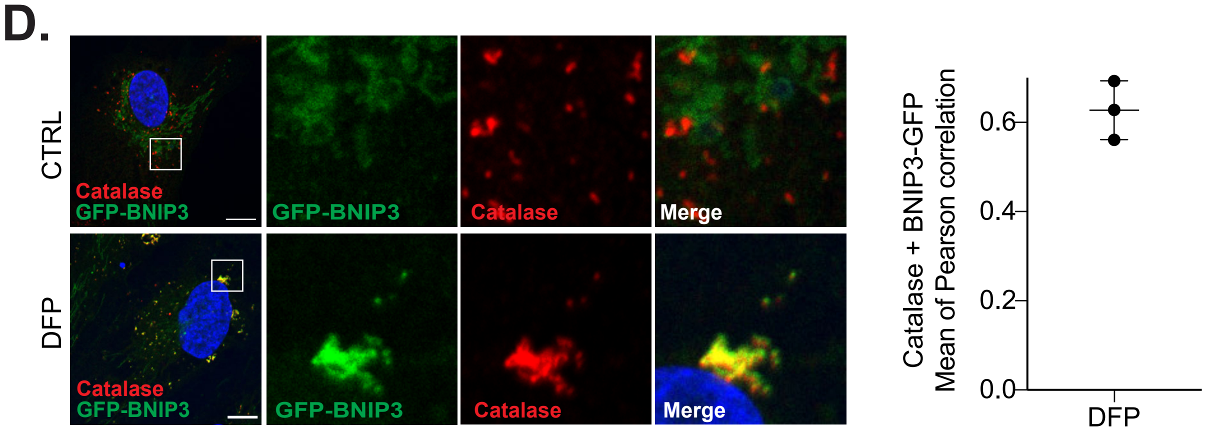

We do not yet know why NIX is preferred over BNIP3 in ARPE19 cells, given both are expressed (a similar situation is seen in the erythrocytes – see below). We have included the additional text from page 9:

"We did notice, especially in the more sensitive reporter-based cell assays, that a small degree of pexophagy still occurred in the absence of NIX. Given our previous data showing a degree of redundancy between NIX and BNIP3, a close homolog of NIX, for DFP-induced mitophagy (Zhao *et al.*, 2020), we reasoned that this residual pexophagy may be due to BNIP3 involvement. To test this, we employed siRNA of both NIX and BNIP3 (Figure EV2). In the *pexo*-QC reporter cells, NIX or BNIP3 were first silenced by siRNA and then cells were treated with DFP to induce pexophagy (Figure EV2A). We observed that the silencing of not only NIX, but also BNIP3, impaired DFP-induced mitoophagy (Figure EV2B). In addition, the silencing of these two proteins together completely abolished DFP-induced pexophagy suggesting that NIX and BNIP3 share similar functions in pexophagy pathway (Figure EV2C). Given the data showing a strong pexophagy block with the more complete loss of NIX in the CRISPR clones (as well as its rescue), we assume that in these cells NIX plays the prime role in mediating pexophagy. However, the data suggests that BNIP3 can also regulate pexophagy and this may be more relevant in other cell types or signalling conditions. More work is needed to confirm this."

2. The authors fail to explore how NIX translocates to peroxisomes after DFP treatment. This is a very important question and a couple of simply experiments will provide some clues. For instance, will artificially targeting NIX to peroxisomes by removal of the TM of NIX and replacing it with peroxisome targeting TM such as that of PEX26 used previously (https://doi.org/10.1083/jcb.201605002) induce pexophagy? Vice versa, please replace the TM of NIX with that of OMP25 to see if that prevents NIX translocation to peroxisomes and pexophagy.

The domain switch experiment was a great suggestion by the reviewer, and we attempted the experiment. However, we found that the peroxisomal targeted construct resulted in a dramatic change in peroxisomal localisation and affected protein import – many cells had cytosolic localisation of the pexo-QC reporter and also catalase. Thus, though interesting, we decided that this large-scale disruption to peroxisomes would make any pexophagy induced very difficult to interpret and we did not further characterise these mutants. We have included the data here for the reviewer:

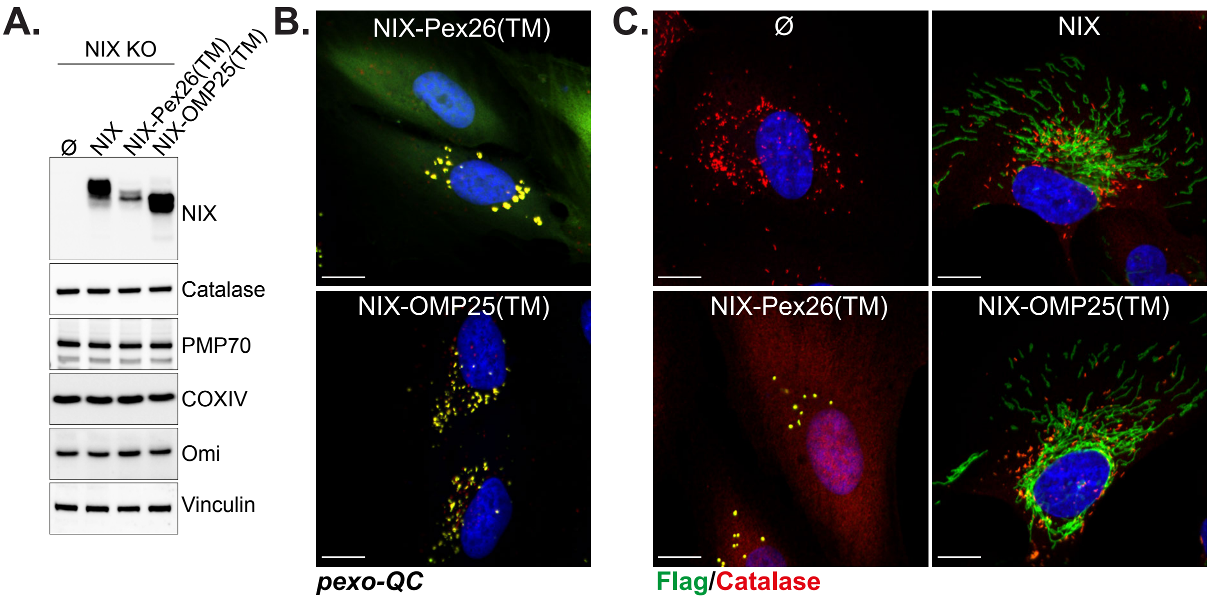

Reviwer Figure: Artificially targeting NIX to peroxisomes or mitochondria
A. Immunoblots of the indicated proteins in lysates of NIX KO ARPE19 cells (Cl 31) stably expressing a pBabe flag vector (Ø), flag-NIX, flag-NIX-Pex26(TM) or flag-NIX-OMP25(TM).
B. Confocal images of cells as in A, stably expressing the *pexo-QC* reporter.
C. Confocal images of cells as in A, immunostained with peroxisomal marker in red (catalase) and anti-flag antibody in green.
Nuclei were stained in blue (Hoechst). Scale bar: 10µm.

We did however characterise a NIX transmembrane-only construct and found that this was sufficient to localise to both mitochondria and peroxisomes – please see Referee 2, point 4.

3. Pexophagy and interfering of peroxisome biogenesis lead to the same phenotype: less of peroxisomes. One way to distinguish the two processes is to see if the peroxisomal loss can be reversed by blocking the general autophagy machinery. Therefore, while it is nice for the authors to show the block of pexophagy in NIX KO mice retina, do they see the same blocking phenotype in FIP200 KO mice? Unfortunately, most of the ATG KO mice are lethal. But the FIP200 KI/KI (knocking of a LQFL/AAAA mutant) is viable. It's a big to ask for such in a revision, but at least it should be considered for future work and discussed in the discussion.
This certainly would be a good experiment; however, it is my understanding that the LQFL KI/KI mice are still neonatal lethal and die not long after birth – unfortunately the eyes are still closed and underdeveloped at this stage. We do agree though, more work is needed to absolutely confirm this in vivo. We have added the following discussion on page 14:
"This suggests a coordination of mitophagy and pexophagy *in vivo*, though further work, for example with tissue-specific knockout of core autophagy genes, would help to confirm this as well as rule out peroxisomal biogenesis defects."

4. NIX translocation to peroxisomes seems not very robust based in the IF images and only a fraction of peroxisomes show NIX localization. To make it much more convincing, the authors should check NIX translocation in Drp1 KO or KD cells, where peroxisomes will become very elongated and filament-like. Will Bafilomycin treatment (blocking pexophagy) enhance NIX translocation? Will hypoxia also cause NIX translocation? Since HIF1 is required for NIX up-regulation, it might also be required for pexophagy. The authors should check this.

We apologise, but we were under the impression that translocation of NIX, both endogenous and GFP-tagged, upon DFP induction was quite striking (for example see image at the top of this report in response to the Editor). It is possible that in the image conversion during submission resulted in fainter images. We will try and increase intensity to hopefully improve the situation.
As suggested, we found that hypoxia induces pexophagy – new Fig.EV1D-E, discussed on page 5:

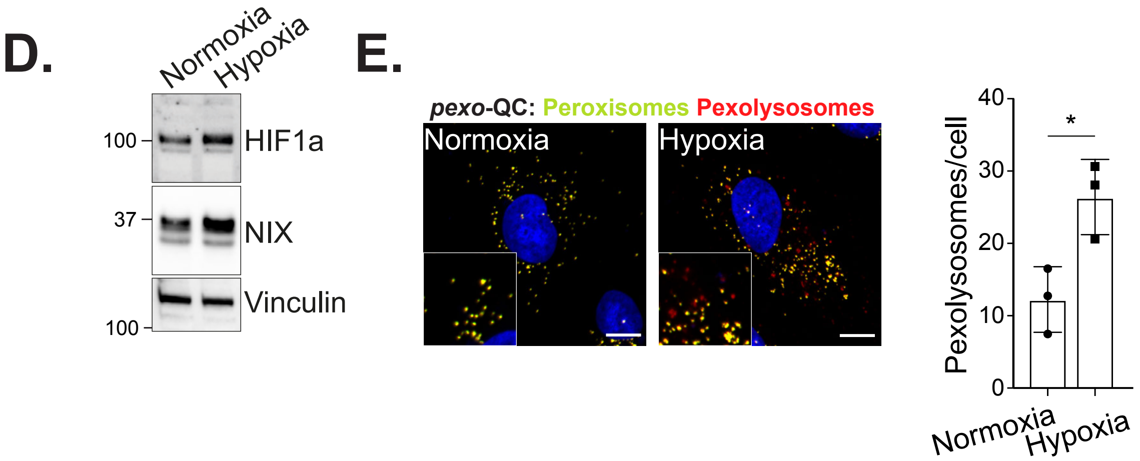

Additionally, hypoxia also stimulates GFP-NIX or endogenous NIX translocation to peroxisomes – new Fig. EV3C, discussed on page 10:

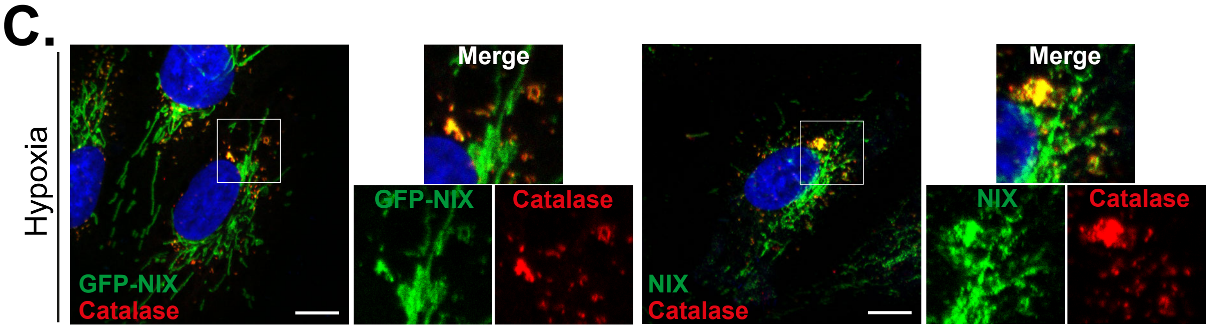

Finally, as expected HIF1a, is required for pexophagy – here we used a previously generated HIF1 KO cell line. These data are in the new Fig. 2A-B and discussed on page 7:

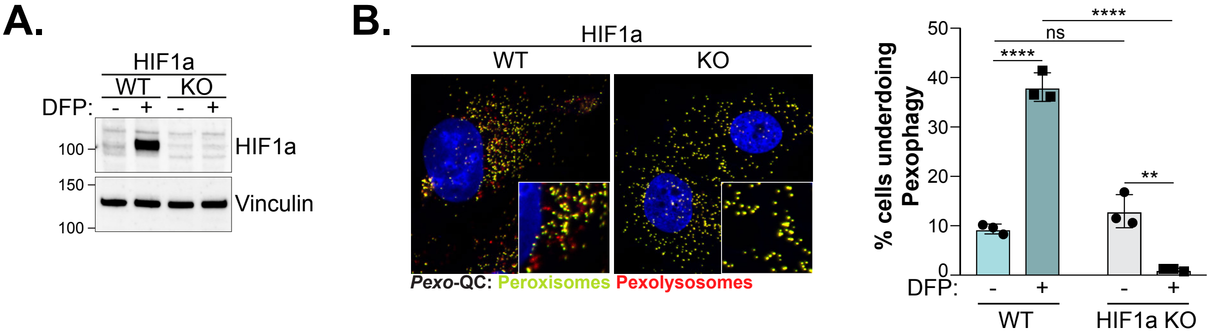

5. In all the WB they performed to show pexophagy, only PMP70 and catalase levels are examined. Some additional peroxisomal markers such as PEX14, PEX2, PEX10, PEX13 or other matrix proteins such as alcohol oxidase (AOx) and 3-ketoacyl-CoA thiolase (ACAA2) should be examined.

To look at additional markers of peroxisomes by western blot, we also probed for PEX5 and PEX19. Satisfyingly all showed a decrease upon DFP treatment, and we hope that this, combined with the exogenous *pexo*-QC reporter, more convincingly demonstrates pexophagy. The new western blot data is shown in new Fig. EV1A and discussed on page 5:

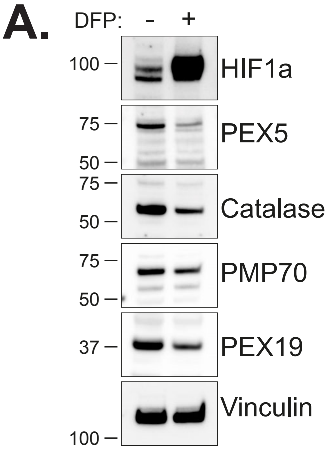

6. The title says "coordinates" which is not really supported by the data. They only show NIX is required for both mitophagy and pexophagy, but coordination of the two processes is not shown. They also state that mitophagy and pexophagy occur sequentially, which is not really clear. Once NIX is unregulated by HIF1, it may initiate the two processes simultaneously. The reason the authors saw slower clearance or degradation of peroxisomes than mitochondria may simply be because there is much less NIX translocated to peroxisomes.
Yes, we agree with the reviewer here that coordination, or that mitophagy-pexophagy are sequential, is not absolutely clear – to avoid overinterpretation we have therefore changed the title to "BNIP3L/NIX regulates both mitophagy and pexophagy" and removed references to sequential turnover.

Minor comments

Fig. 1B. In addition to the count of the number of pexolysosomes/cell, the number of peroxisomes/cell should be counted as well to determine if some of the peroxisomes are completely cleared.
Our semi-automated method of quantitation (mito-QC counter: see Methods) does indeed partially measure this and shows that the mean green signal intensity (peroxisomal) does indeed decrease from a value of 452.5 to 338.3 upon DFP treatment – suggesting clearance of peroxisomes. However, as we have already included similar data by FACS analysis of the reporter (which is a ratio of mCherry:GFP – see for example Fig. EV1I) as well as showing loss of 4 different peroxisomal proteins, we would rather not show an additional panel showing the same thing in an already crowded figure. Hope this is OK.

Fig. 1D. It's odd that LC3 expression is dramatically increased in both siATG13 and siULK1 even without DFP treatment. Also, LC3 lipidation seems to be completely blocked in siATG13 but not siULK1. Can the authors explain?
Yes, this is perhaps somewhat unexpected on one level, and we do not know the exact reason(s) for this. This could reflect the block in turnover of general autophagy, or an attempt by the cell to transcriptionally upregulate LC3 to try and overcome the loss of the ULK1 complex. The small degree of LC3 lipidation that is observed in the ULK1 siRNA cells

could be due to incomplete depletion of ULK1, a compensation by ULK2, or the alternate conjugation of LC3 to single membrane pathways that do not require the ULK1 complex for LC3 lipidation (Galluzzi & Green, 2019). Regardless, loss of ATG13 or ULK1 impairs the flux of mitochondrial and peroxisomal proteins, demonstrating that both DFP-induced mitophagy and pexophagy require the conventional autophagy machinery. We have added possible explanations to the text on page 7:
"Interestingly, the levels of LC3-I were strongly upregulated in both the ATG13 and ULK1 siRNA-treated cells, which is likely reflective of impaired autophagy in general (Figure 1D). Taken together, loss of ATG13 or ULK1 impairs the flux of mitochondrial and peroxisomal proteins, demonstrating that both DFP-induced mitophagy and pexophagy require the conventional autophagy machinery."

Fig. 2B. While pS65 Ub is not detected in DFP or Ivermectin treated cells, it can't be ruled out that other types of ubiquitination could be involved. Indeed, Sulkshane et al in a recent paper (Redox Biology, 2021, https://doi.org/10.1016/j.redox.2021.102047) showed that increased ubiquitin levels was detected on mitochondria after $CoCl_2$ treatment or under hypoxic conditions. So, ideally, subcellular fractionation should be checked to see if more ubiquitin can be detected on mitochondria after DFP treatment.
We agree with the Reviewer here on the role of ubiquitin in this pathway, but our original aim was just to say that ubiquitylation is not induced in the same way as for the known mitophagy stimulators such as ivermectin/CCCP etc. Ubiquitin is undoubtably involved at some level in here, but the aim of this current work is to focus on NIX. Given this, we have moved these data to Figure EV5A-B. See also next point.

Fig. 2C. It's odd that FK2 staining detected some puncta in the control but not in DFP samples. The IF should be repeated with co-staining of Tom20 to see if those puncta are on mitochondria.
DFP treatment does consistently lead to less cytosolic ubiquitin puncta, and this is certainly intriguing - but we currently do not know why. However, as mentioned in the above point, we are not focussed on ubiquitylation here (which would require a significant amount of extra work). However, this observation fits with our hypothesis that if ubiquitylation is occurring it is likely distinct from the CCCP/Ivermectin pathways. We have added the following statement on page 14 when discussing these data:
"Ubiquitin is likely involved in DFP-induced pexophagy at multiple stages, but further work is needed to determine the exact involvement and its precise roles."

Fig. 5C. It's surprising that NIX levels are similar between untreated and DFP treated cells. Isn't NIX supposed to be induced by DFP treatment (compare to Fig. EV3A)? Why is NIX not degraded like other mitochondrial proteins after 48h treatment? Is it because NIX is constantly induced? In this case, NIX should not be on mitochondria anymore, right?
We apologise for the confusion (as was also evident from point 5 raised by Referee 2). We think that NIX is indeed induced in this experiment and degraded along with the mitochondrial proteins. However, the conditions of this specific experiment differ from the others due to the much longer time course (24h -/+ CCCP, then 24h recovery, then 48h DFP) and the non-CCCP treated cells are much more confluent compared to the CCCP treated. We have found confluency influences NIX expression and under these conditions NIX is not transcriptionally induced as highly, hence its overall levels do not increase as much due to

autophagic turnover. However, as the non-CCCP treated cells are not used in our colocalization experiments or discussed in the text (and the best control is already present (WT vs Parkin overexpression)), we have cropped the blot showing the non-CCCP treated samples to avoid confusion (new Fig. 4B). Regardless, the main point of the experiment was to show that NIX could translocate to peroxisomes in the absence of mitochondria, which does appear to be the case.

Fig. 6A. While PMP70 staining shows the normal peroxisomal localization (spots), catalase staining does not show the pattern like PMP70, more like diffuse and cytosolic. Why? Mitochondrial marker should be also checked to verify the mitophagy is blocked in NIX KO retina.
Thanks for raising this point – we think this is simply due to the primary antibody "quality" and in this tissue it does not give a nice clear punctate staining pattern (though the images are purposefully at low resolution to show the characteristic cellular organisation of the retina). Regardless, the staining does decrease in-line with PMP70 staining, strongly suggesting we are looking at peroxisomes. As mentioned in point 3 above, disruption of a core autophagy gene in the retina would be a good control here but this is currently beyond our means for this work.
We had a limited supply of adult NIX KO eyes from our collaborator and further analyses of mitochondria are being carried out in a new inducible NIX KO mouse model (P. Boya Lab). Given the novelty of this model, these data will be presented in a separate manuscript.

Fig. EV1F. The FACS plots show that the GFP/mCherry ratio is already quite different in each cell samples without treatment, indicating the basal pexophagy level is different. It should be adjusted to 1% individually so that the effect of DFP over control can be properly measured.
We apologise for the lack of clarity here. The gating was set at 7% for mito-QC and 11% for pexo-QC experiments. These values were chosen (and kept constant) based on microscopy data that showed 7% of mito-QC cells had more red-only puncta than the mean value and 11% of pexo-QC cells had more red-only compared to the mean value. Hence we used this cut-off to indicate cells undergoing mitophagy/pexophagy. Loss of ULK1 or ATG13 does slightly reduce basal levels, however there is no significant induction with DFP. Importantly, the data obtained under these conditions matched the reporter independent western blot data in Fig. 1D. We have added to the Methods section on page 23 to explain this:
"The gate used for the non-treated condition or control cells, was applied to all the other conditions. The value used for this was based on quantitation of microscopy data from *mito*-QC/*pexo*-QC cells that showed 7% (*mito*-QC) or 10% (*pexo*-QC) of cells had red-only puncta above the value of the mean."

Fig. EV3A. Why is the exogenous NIX (from pBabe-NIX) significantly reduced in the rescued cells (KO + pBabe-NIX) but endogenous NIX is not degraded (WT)?
We apologise as perhaps this was not well explained in the original text. Transcription of endogenous NIX (via HIF1) is significantly increased over the level of autophagic turnover, hence protein increases. The exogenous NIX is not under the endogenous promoter, so it is not transcriptionally upregulated with DFP. In this scenario, its rate of degradation exceeds its rate of transcription and therefore total levels decrease. The old Fig. EV3A is now the new Fig. 2F and we have included the following text on page 8 to hopefully clarify this:

“It is also important to note that the endogenous and exogenous protein levels of NIX differ in response to DFP. This is likely because endogenous NIX has the HIF1-responsive elements within its promoter and is strongly induced upon DFP treatment, to a level that exceeds the rate of mitophagy/pexophagy, hence it accumulates. In contrast, exogenous NIX has no such promoter elements and is expressed constitutively at a rate that does not exceed that of its turnover via mitophagy/pexophagy, hence its levels decrease with DFP treatment.”

Fig. EV6D. It is understandable that GFP-NIX is degraded with DFP treatment. But why is GFP-NiXdeltaTM also degraded with DFP? This truncated NIX would be cytosolic and shouldn't be degraded by mtiophagy or pexophagy.
We think that this maybe due to another DFP-dependent cellular effect on exogenous NIX expression rather than its degradation. For example, nucleotide polymerases require iron-sulphur clusters that could be indirectly influenced by iron chelation. Regardless, the aim of the experiment was to show that the NIX constructs did or did not localise to peroxisomes and mitochondria, which we feel was clearly demonstrated. To avoid confusion we have removed this blot.

Fig. EV6C-6D. In 6C, PMP70 degradation is significant but not catalase. But in 6D, it's the opposite - catalase degradation is very obvious but not PMP70. Why? NIX levels seems to be very high from Day 0 of differentiation and don't change much on Day 4 and Day 8. Can the authors explain this? In both 6C-6D, HIF1 levels should be checked as well as BNIP3. It's mysterious that NIX but not BNIP3 is required for mitochondria clearance in reticulocytes. Therefore, it will be very informative to show if HIF1 induction of BNIP3 is not present in these situation.
We thank the reviewer for these comments. We probed these blots for both HIF1a and BNIP3, which are indeed present (see new Fig. EV6C). Much work has shown that NIX is important for mitophagy in these cells, but BNIP3 is also present and it is possible that it may be able to compensate to some degree in the absence of NIX. We do not know why NIX is preferred in reticulocyte development but it is – however, without knowing exact copy numbers of NIX vs BNIP3, it is possible that NIX is present in a much higher excess. We also know that NIX level is not sufficient on its own to drive significant mitophagy/pexophagy so other factors are obviously at play too. Interestingly, this situation is similar to the APRE-19 cells that express both NIX and BNIP3 (see point 1). More work is needed to determine the NIX vs BNIP preference.
The stability of catalase is an important control as it is not peroxisomal in erythrocytes. Perhaps this was not made clear in the text. We have now added to this on page 15:
“We also observed that the peroxisomal marker PMP70 was progressively lost upon differentiation. In contrast, the expression of catalase was not appreciably affected after 20 days of culture (Figure EV6C). However, catalase is a highly active enzyme in erythrocytes, being required for ROS protection and is found free in the cytosol or associated with the plasma membrane (Aviram & Shaklai, 1981; Rocha *et al*, 2015). Thus, uniquely, catalase level is not a marker for peroxisome content in erythroid cells and hence a good control for peroxisome specificity in this experiment.”
In H9C2 cells, mitochondria and peroxisomes are not completely cleared and here biogenesis (which is well characterised for mitochondria) is likely occurring making it difficult to conclude autophagic turnover from western blot alone – hence the need for the -QC reporter data. This was previously mentioned in the text:

"As expected, mitochondrial biogenesis was evident in *mito*-QC cells as observed by the enhanced levels of mitochondrial HSP60 and COXIV, and GFP. Interestingly, the same was true for peroxisomes in the *pexo*-QC cells: increased peroxisomal marker PMP70 as well as GFP (Figure EV6D)."

Page 12. Please explain Band3 and alpha-4-integrin a little bit so readers can understand why you checked these two markers.

We apologise for this omission and have now included the following text on page 15:

"Cells at different stages of maturation were collected and differentiation status was confirmed by staining and flow cytometry of Band3, an anion exchanger and marker of mature erythrocytes, and α-4-integrin, a cell surface adhesion molecule that is lost during differentiation (Figure EV6A) (details previously described (Hu *et al*, 2013)."

Dear Ian,

We have now received re-review reports from two of the three referees who originally saw your manuscript; I have included these at the bottom of this email. You have addressed the concerns of one of the referees satisfactorily. However, the other requests some additional material be included. This request is, I am convinced, in the spirit of tightening the conclusions of what will be an important study. I would be grateful if you could carefully consider the points raised. In addition, there are some remaining editorial points which need addressing.

Here, would you please:
limit the number of keywords to five.
Rename the "Conflict of Interests" statement as "Disclosure and competing interests statement".
Remove the author contributions section from the ms.

We encourage the publication of source data, particularly for electrophoretic gels and blots, with the aim of making primary data more accessible and transparent to the reader. It would be great if you could provide me with a PDF file per figure that contains the original, uncropped and unprocessed scans of all or key gels used in the figures. The PDF files should be labeled with the appropriate figure/panel number, and should have molecular weight markers; further annotation could be useful but is not essential. The PDF files will be published online with the article as supplementary "Source Data" files. Source Data can also include Excel tables to accompany your graphs. We anticipate that their inclusion will make your work more discoverable and useable to scientists in the future.

Regarding the figures, there are also some minor changes to be made. Would you please:
Remove references to Fig 5E&F, which don't exist.
In Figure 1B & C, Figure 2 B, D & E, Figure 5 C & D, Figure EV1 E and Figure EV5 F, please highlight the areas in the larger images from which the zoomed areas were taken.
Add scale bars to Figure 5A i - ii.
Include Figure EV4 and its legend. These are currently missing.

We include a synopsis of the paper (see http://emboj.embopress.org/). Please provide me with a general summary statement and 3-5 bullet points that capture the key findings of the paper.
We also need a summary figure for the synopsis. The size should be 550 wide by [200-400] high (pixels). You can also use something from the figures if that is easier.
EMBO Press is an editorially independent publishing platform for the development of EMBO scientific publications.

Best wishes,

William

William Teale, PhD
Editor
The EMBO Journal
w.teale@embojournal.org

Instructions for preparing your revised manuscript:

Please check that the title and abstract of the manuscript are brief, yet explicit, even to non-specialists.

When assembling figures, please refer to our figure preparation guideline in order to ensure proper formatting and readability in print as well as on screen:
https://bit.ly/EMBOPressFigurePreparationGuideline
See also figure legend guidelines: https://www.embopress.org/page/journal/14602075/authorguide#figureformat

IMPORTANT: When you send the revision we will require
- a point-by-point response to the referees' comments, with a detailed description of the changes made (as a word file).
- a word file of the manuscript text.
- individual production quality figure files (one file per figure)
- a complete author checklist, which you can download from our author guidelines

(https://www.embopress.org/page/journal/14602075/authorguide).
- Expanded View files (replacing Supplementary Information)
Please see out instructions to authors
https://www.embopress.org/page/journal/14602075/authorguide#expandedview

Please remember: Digital image enhancement is acceptable practice, as long as it accurately represents the original data and conforms to community standards. If a figure has been subjected to significant electronic manipulation, this must be noted in the figure legend or in the 'Materials and Methods' section. The editors reserve the right to request original versions of figures and the original images that were used to assemble the figure.

Further information is available in our Guide For Authors: https://www.embopress.org/page/journal/14602075/authorguide

We realize that it is difficult to revise to a specific deadline. In the interest of protecting the conceptual advance provided by the work, we recommend a revision within 3 months (1st Dec 2022). Please discuss the revision progress ahead of this time with the editor if you require more time to complete the revisions. Use the link below to submit your revision:

https://emboj.msubmit.net/cgi-bin/main.plex

Referee #2:

In this revised manuscript, Wilhelm et al. provided additional data and descriptions to address most of the issues pointed by the referees. In particular, the data that pexophagy was suppressed in cells expressing the LIR (W36A/ L39A) and dimerization motif (G204A) mutants further support the idea that NIX promotes both mitophagy and pexophagy in a similar manner. Additionally, a fraction of "GFP-NIX TM only" can localize to peroxisomes in DFP-treated cells, suggesting that the TM domain contains necessary and sufficient information for both mitochondrial and peroxisomal targeting. Moreover, the authors showed that NIX localizes to peroxisomes and promote pexophagy under hypoxic conditions, and that DFP-induced pexophagy requires HIF1a. These data sets have nicely been rearranged and described in the main text. There are, however, still several points that need to be clarified in order to substantiate their conclusions as indicated below.

1. In Figure 3C, the authors should add a series of fluorescent images under control conditions. These observations are critical and interesting particularly for "GFP-NIX TM only", as it remains possible that the NIX TM may act as a dual targeting signal for both mitochondria and peroxisomes in response to HIF1a signaling, or that the NIX cytosolic domain may contain a regulatory element to suppress its peroxisomal targeting under normoxic conditions.

2. The authors should confirm peroxisomal localization of the NIX (or GFP-NIX) W36A/L39A and G204A mutants, which can be shown in Figure EV5, and exclude the possibility that their pexophagy defects are caused by loss of peroxisomal targeting.

3. In Figure 5A, the authors should perform the same fluorescence imaging for DFP + BafA-treated cells lacking NIX (and/or expressing the W36A/L39A mutant) and verify that peroxisomal localization of LC3 depends on NIX (and/or the LIR motif).

4. In the discussion part (page 17, line 1), the authors mentioned that NIX peroxisomal targeting requires its LIR motif. There is no data indicating this point in the revised manuscript, and it is nevertheless unlikely to be the case. Thus, this description should be deleted in the main text.

Referee #3:

The authors have adequately addressed my comments and I support acceptance.

EMBOJ-2022-111115

Once again, we thank the Editor and Reviewers for all their help with our manuscript.

Response to Editor

limit the number of keywords to five.
Done, now: Autophagy, mitochondria, mitophagy, peroxisomes, pexophagy

Rename the "Conflict of Interests" statement as "Disclosure and competing interests statement".
Done

Remove the author contributions section from the ms.
Done

We encourage the publication of source data, particularly for electrophoretic gels and blots, with the aim of making primary data more accessible and transparent to the reader. It would be great if you could provide me with a PDF file per figure that contains the original, uncropped and unprocessed scans of all or key gels used in the figures. The PDF files should be labeled with the appropriate figure/panel number, and should have molecular weight markers; further annotation could be useful but is not essential. The PDF files will be published online with the article as supplementary "Source Data" files. Source Data can also include Excel tables to accompany your graphs. We anticipate that their inclusion will make your work more discoverable and useable to scientists in the future.
We have now included annotated western blot source data for each figure.

Regarding the figures, there are also some minor changes to be made. Would you please:
Remove references to Fig 5E&F, which don't exist.
Done

In Figure 1B & C, Figure 2 B, D & E, Figure 5 C & D, Figure EV1 E and Figure EV5 F, please highlight the areas in the larger images from which the zoomed areas were taken.
Done

Add scale bars to Figure 5A i - ii.
Done
Include Figure EV4 and its legend. These are currently missing.
We originally did not have an EV4 figure but in the revised version we have now included one with data from the previous EV5.

We include a synopsis of the paper (see http://emboj.embopress.org/). Please provide me with a general summary statement and 3-5 bullet points that capture the key findings of the paper.
We have written this:

BNIP3L/NIX is an autophagic receptor that dually localizes to mitochondria and peroxisomes to regulate selective autophagy of these organelles. Thus, NIX controls the degradation of the two major eukaryotic metabolic organelles.

- Iron chelation stimulates multiple selective autophagy pathways
- Mitophagy and pexophagy are both dependant on NIX
- NIX is targeted to peroxisomes via its transmembrane domain to regulate pexophagy
- NIX-dependent pexophagy occurs under physiological processes

We also need a summary figure for the synopsis. The size should be 550 wide by [200-400] high (pixels).

This is now included in the resubmission:

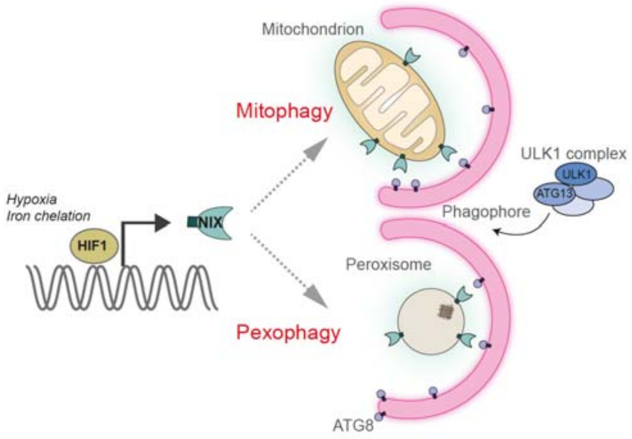

Response to Reviewer 2

1. In Figure 3C, the authors should add a series of fluorescent images under control conditions. These observations are critical and interesting particularly for "GFP-NIX TM only", as it remains possible that the NIX TM may act as a dual targeting signal for both mitochondria and peroxisomes in response to HIF1a signaling, or that the NIX cytosolic domain may contain a regulatory element to suppress its peroxisomal targeting under normoxic conditions.

We already have the control (non-DFP) samples shown in Fig. EV3E – this is with NIX and mitochondrial stain. Here we show that it is nearly all mitochondrial (as we had shown in Fig 3A and B) and the GFP-TM-only is the same (the NIX cytosolic domain is always cytosolic). Our conclusions are indeed that the NIX-TM domain is a dual targeting signal (as it behaves as WT and localises to peroxisomes upon DFP). We state at the bottom of page 10: "*These data indicate that NIX localization to mitochondria and peroxisomes relies on the same targeting signal, present within the transmembrane domain, and suggests that NIX becomes an integral peroxisomal protein.*"

2. The authors should confirm peroxisomal localization of the NIX (or GFP-NIX) W36A/L39A and G204A mutants, which can be shown in Figure EV5, and exclude the possibility that their pexophagy defects are caused by loss of peroxisomal targeting.

The Reviewer has a valid point here – in the rush to do all the extra experiments, this was overlooked and is now included in the new Fig. EV5:

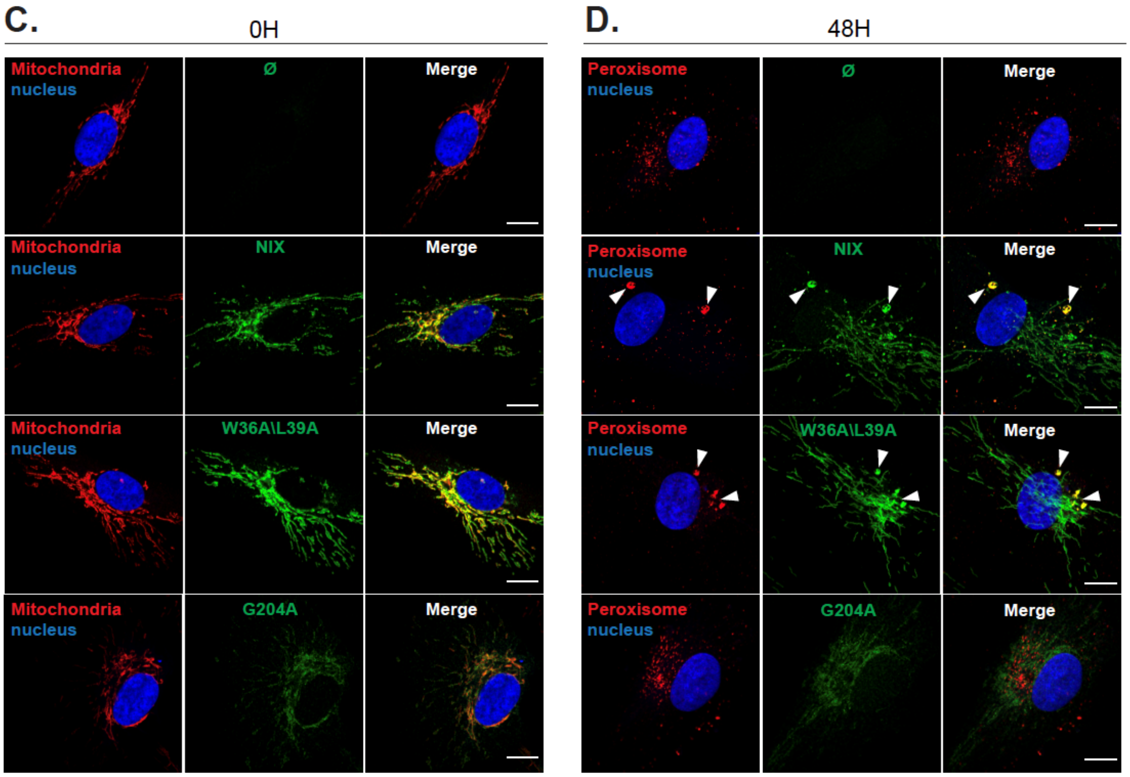

The LIR mutant is not mislocalised and is described on page 13: "*We noted that the W36A/L39A LIR mutant was localized to mitochondria under control conditions (Figure EV5C) and was enriched on peroxisomes upon DFP (Figure EV5D), excluding the possibility that pexophagy defects are caused by loss of peroxisomal targeting*."

The dimer mutant is slightly mislocalised to cystosol and nucleus and expressed to a lower level (as previously shown). Text has been added to highlight this on page 14: "*In addition, we also found that G204A mutation impaired NIX expression and localization. Indeed, G204A levels were lower compared to WT NIX (Figure EV5A) and the G204A dimer mutant was observed not only at the mitochondria but also diffuse in the cytoplasm and nucleus (Figure EV5C). Upon DFP, G204A dimer mutant was not dramatically enriched on peroxisomes (Figure EV5D), suggesting that the transmembrane region of NIX is required for NIX dimerization, efficient localization at the mitochondria and peroxisomes and likely NIX stability*."

3. In Figure 5A, the authors should perform the same fluorescence imaging for DFP + BafA-treated cells lacking NIX (and/or expressing the W36A/L39A mutant) and verify that peroxisomal localization of LC3 depends on NIX (and/or the LIR motif).
Following discussion with the editor, we feel that we have already demonstrated a role for LC3 binding during pexophagy. In the revision, the key mechanistic and functional experiments show that the NIX LIR mutant impairs both mitophagy and pexophagy, which is very good evidence that LC3 (and/or otherATG8s) are important for the process. We do not feel that this is controversial in any way and is a clear result.

4. In the discussion part (page 17, line 1), the authors mentioned that NIX peroxisomal targeting requires its LIR motif. There is no data indicating this point in the revised manuscript, and it is nevertheless unlikely to be the case. Thus, this description should be deleted in the main text.

This is certainly a valid point and a good spot by the Reviewer, and we have corrected the mistake to remove mention of the LIR. The sentence now reads: "…*we identified that NIX is also a peroxisomal protein, whose targeting occurs independently of mitochondria and requires the NIX transmembrane domain.*"

Dear Ian,

I am pleased to inform you that your manuscript has been accepted for publication in the EMBO Journal.

Congratulations on a really nice study! It was a pleasure to work on.

Please note that it is EMBO Journal policy for the transcript of the editorial process (containing referee reports and your response letter) to be published as an online supplement to each paper. If you do NOT want this, you will need to inform the Editorial Office via email immediately. More information is available here:
https://www.embopress.org/page/journal/14602075/authorguide#transparentprocess

Your manuscript will be processed for publication in the journal by EMBO Press. Manuscripts in the PDF and electronic editions of The EMBO Journal will be copy edited, and you will be provided with page proofs prior to publication. Please note that supplementary information is not included in the proofs.

You will be contacted by Wiley Author Services to complete licensing and payment information. The required 'Page Charges Authorization Form' is available here: https://www.embopress.org/pb-assets/embo-site/tej_apc.pdf - please download and complete the form and return to embopressproduction@wiley.com

EMBO Press participates in many Publish and Read agreements that allow authors to publish Open Access with reduced/no publication charges. Check your eligibility: https://authorservices.wiley.com/author-resources/Journal-Authors/open-access/affiliation-policies-payments/index.html

Should you be planning a Press Release on your article, please get in contact with embojournal@wiley.com as early as possible, in order to coordinate publication and release dates.

If you have any questions, please do not hesitate to call or email the Editorial Office. Thank you for your contribution to The EMBO Journal.

Yours sincerely,

William

William Teale, PhD
Editor
The EMBO Journal
w.teale@embojournal.org

** Click here to be directed to your login page: https://emboj.msubmit.net

EMBO Press Author Checklist

Corresponding Author Name: IAN GANLEY	
Journal Submitted to: EMBO JOURNAL	
Manuscript Number: EMBOJ-2022-111115	

USEFUL LINKS FOR COMPLETING THIS FORM
The EMBO Journal - Author Guidelines
EMBO Reports - Author Guidelines
Molecular Systems Biology - Author Guidelines
EMBO Molecular Medicine - Author Guidelines

Reporting Checklist for Life Science Articles (updated January
This checklist is adapted from Materials Design Analysis Reporting (MDAR) Checklist for Authors. MDAR establishes a minimum set of requirements in transparent reporting in the life sciences (see Statement of Task: 10.31222/osf.io/9sm4x). Please follow the journal's guidelines in preparing your
Please note that a copy of this checklist will be published alongside your article.

Abridged guidelines for figures
1. Data
The data shown in figures should satisfy the following conditions:
- the data were obtained and processed according to the field's best practice and are presented to reflect the results of the experiments in an accurate and unbiased manner.
- ideally, figure panels should include only measurements that are directly comparable to each other and obtained with the same assay.
- plots include clearly labeled error bars for independent experiments and sample sizes. Unless justified, error bars should not be shown for technical
- if n<5, the individual data points from each experiment should be plotted. Any statistical test employed should be justified.
- Source Data should be included to report the data underlying figures according to the guidelines set out in the authorship guidelines on Data

2. Captions
Each figure caption should contain the following information, for each panel where they are relevant:
- a specification of the experimental system investigated (eg cell line, species name).
- the assay(s) and method(s) used to carry out the reported observations and measurements.
- an explicit mention of the biological and chemical entity(ies) that are being measured.
- an explicit mention of the biological and chemical entity(ies) that are altered/varied/perturbed in a controlled manner.
- the exact sample size (n) for each experimental group/condition, given as a number, not a range;
- a description of the sample collection allowing the reader to understand whether the samples represent technical or biological replicates (including how many animals, litters, cultures, etc.).
- a statement of how many times the experiment shown was independently replicated in the laboratory.
- definitions of statistical methods and measures:
  - common tests, such as t-test (please specify whether paired vs. unpaired), simple χ2 tests, Wilcoxon and Mann-Whitney tests, can be unambiguously identified by name only, but more complex techniques should be described in the methods section;
  - are tests one-sided or two-sided?
  - are there adjustments for multiple comparisons?
  - exact statistical test results, e.g., P values = x but not P values < x;
  - definition of 'center values' as median or average;
  - definition of error bars as s.d. or s.e.m.

Please complete ALL of the questions below.
Select "Not Applicable" only when the requested information is not relevant for your study.

Materials

Newly Created Materials	Information included in the manuscript?	In which section is the information available? (Reagents and Tools Table, Materials and Methods, Figures, Data Availability Section)
New materials and reagents need to be available; do any restrictions apply?	Not Applicable	

Antibodies	Information included in the manuscript?	In which section is the information available? (Reagents and Tools Table, Materials and Methods, Figures, Data Availability Section)
For **antibodies** provide the following information:<br>- Commercial antibodies: RRID (if possible) or supplier name, catalogue number and or/clone number<br>- Non-commercial: RRID or citation	Yes	Materials and Methods

DNA and RNA sequences	Information included in the manuscript?	In which section is the information available? (Reagents and Tools Table, Materials and Methods, Figures, Data Availability Section)
**Short novel DNA or RNA including primers, probes**: provide the sequences.	Yes	Materials and Methods

Cell materials	Information included in the manuscript?	In which section is the information available? (Reagents and Tools Table, Materials and Methods, Figures, Data Availability Section)
**Cell lines:** Provide species information, strain. Provide accession number in repository **OR** supplier name, catalog number, clone number, and/**OR** RRID.	Yes	Materials and Methods
**Primary cultures:** Provide species, strain, sex of origin, genetic modification status.	Yes	Materials and Methods
Report if the cell lines were recently **authenticated** (e.g., by STR profiling) and tested for mycoplasma contamination.	Yes	Materials and Methods

Experimental animals	Information included in the manuscript?	In which section is the information available? (Reagents and Tools Table, Materials and Methods, Figures, Data Availability Section)
**Laboratory animals or Model organisms:** Provide species, strain, sex, age, genetic modification status. Provide accession number in repository **OR** supplier name, catalog number, clone number, **OR** RRID.	Not Applicable	
**Animal observed in or captured from the field:** Provide species, sex, and age where possible.	Not Applicable	
Please detail **housing and husbandry conditions**.	Not Applicable	

Plants and microbes	Information included in the manuscript?	In which section is the information available? (Reagents and Tools Table, Materials and Methods, Figures, Data Availability Section)
**Plants:** provide species and strain, ecotype and cultivar where relevant, unique accession number if available, and source (including location for collected wild specimens).	Not Applicable	
**Microbes:** provide species and strain, unique accession number if available, and source.	Not Applicable	

Human research participants	Information included in the manuscript?	In which section is the information available? (Reagents and Tools Table, Materials and Methods, Figures, Data Availability Section)
If collected and within the bounds of privacy constraints report on age, sex and gender or ethnicity for all study participants.	Not Applicable	

Core facilities	Information included in the manuscript?	In which section is the information available? (Reagents and Tools Table, Materials and Methods, Figures, Data Availability Section)
If your work benefited from core facilities, was their service mentioned in the acknowledgments section?	Not Applicable	

Design

Study protocol	Information included in the manuscript?	In which section is the information available? (Reagents and Tools Table, Materials and Methods, Figures, Data Availability Section)
If study protocol has been **pre-registered, provide DOI in the manuscript**. For clinical trials, provide the trial registration number **OR** cite DOI.	Not Applicable	
Report the **clinical trial registration number** (at ClinicalTrials.gov or equivalent), where applicable.	Not Applicable	

Laboratory protocol	Information included in the manuscript?	In which section is the information available? (Reagents and Tools Table, Materials and Methods, Figures, Data Availability Section)
Provide DOI OR other citation details if **external detailed step-by-step protocols** are available.	Not Applicable	

Experimental study design and statistics	Information included in the manuscript?	In which section is the information available? (Reagents and Tools Table, Materials and Methods, Figures, Data Availability Section)
Include a statement about **sample size** estimate even if no statistical methods were used.	Not Applicable	
Were any steps taken to minimize the effects of subjective bias when allocating animals/samples to treatment (e.g. **randomization procedure**)? If yes, have they been described?	Not Applicable	
Include a statement about **blinding** even if no blinding was done.	Yes	Materials and Methods
Describe **inclusion/exclusion criteria** if samples or animals were excluded from the analysis. Were the criteria pre-established?<br><br>If sample or data points were omitted from analysis, report if this was due to attrition or intentional exclusion and provide justification.	Not Applicable	
For every figure, are **statistical tests** justified as appropriate? Do the data meet the assumptions of the tests (e.g., normal distribution)? Describe any methods used to assess it. Is there an estimate of variation within each group of data? Is the variance similar between the groups that are being statistically compared?	Yes	Materials and Methods and Figure Legends

Sample definition and in-laboratory replication	Information included in the manuscript?	In which section is the information available? (Reagents and Tools Table, Materials and Methods, Figures, Data Availability Section)
In the figure legends: state number of times the experiment was **replicated** in laboratory.	Yes	Figure Legends
In the figure legends: define whether data describe **technical or biological replicates**.	Yes	Figure Legends

Ethics

Ethics	Information included in the manuscript?	In which section is the information available? (Reagents and Tools Table, Materials and Methods, Figures, Data Availability Section)
Studies involving **human participants**: State details of **authority granting ethics approval** (IRB or equivalent committee(s), provide reference number for approval.	Yes	Materials and Methods
Studies involving **human participants**: Include a statement confirming that **informed consent** was obtained from all subjects and that the experiments conformed to the principles set out in the WMA Declaration of Helsinki and the Department of Health and Human Services Belmont Report.	Yes	Materials and Methods
Studies involving **human participants:** For publication of **patient photos**, include a statement confirming that consent to publish was obtained.	Not Applicable	
Studies involving experimental **animals**: State details of **authority granting ethics approva**l (IRB or equivalent committee(s), provide reference number for approval. Include a statement of compliance with ethical regulations.	Not Applicable	
Studies involving **specimen and field samples:** State if relevant **permits** obtained, provide details of authority approving study; if none were required, explain why.	Not Applicable	

Dual Use Research of Concern (DURC)	Information included in the manuscript?	In which section is the information available? (Reagents and Tools Table, Materials and Methods, Figures, Data Availability Section)
Could your study fall under dual use research restrictions? Please check biosecurity documents and list of **select agents and toxins** (CDC): https://www.selectagents.gov/sat/list.htm	Not Applicable	
If you used a select agent, is the security level of the lab appropriate and reported in the manuscript?	Not Applicable	
If a study is subject to dual use research of concern regulations, is the name of the **authority granting approval and reference number** for the regulatory approval provided in the manuscript?	Not Applicable	

Reporting

The MDAR framework recommends adoption of discipline-specific guidelines, established and endorsed through community initiatives. Journals have their own policy about requiring specific guidelines and recommendations to complement MDAR.

Adherence to community standards	Information included in the manuscript?	In which section is the information available? (Reagents and Tools Table, Materials and Methods, Figures, Data Availability Section)
State if relevant guidelines or checklists (e.g., **ICMJE, MIBBI, ARRIVE, PRISMA**) have been followed or provided.	Not Applicable	
For **tumor marker prognostic studies,** we recommend that you follow the **REMARK** reporting guidelines (see link list at top right). See author guidelines, under 'Reporting Guidelines'. Please confirm you have followed these guidelines.	Not Applicable	
For **phase II and III randomized controlled trials**, please refer to the **CONSORT** flow diagram (see link list at top right) and submit the CONSORT checklist (see link list at top right) with your submission. See author guidelines, under 'Reporting Guidelines'. Please confirm you have submitted this list.	Not Applicable	

Data Availability

Data availability	Information included in the manuscript?	In which section is the information available? (Reagents and Tools Table, Materials and Methods, Figures, Data Availability Section)
Have **primary datasets** been deposited according to the journal's guidelines (see 'Data Deposition' section) and the respective accession numbers provided in the Data Availability Section?	Not Applicable	
Were **human clinical and genomic datasets** deposited in a public access-controlled repository in accordance to ethical obligations to the patients and to the applicable consent agreement?	Not Applicable	
Are **computational models** that are central and integral to a study available without restrictions in a machine-readable form? Were the relevant accession numbers or links provided?	Not Applicable	
If publicly available data were reused, provide the respective **data citations in the reference list**.	Not Applicable	
